# Gut microbiota-driven remodeling of fresh and oxidized edible oils revealed by integrated GC-MS and UPLC-HRMS/MS metabolomics

**DOI:** 10.1038/s41538-026-00861-0

**Published:** 2026-05-05

**Authors:** Sherine El-Shamy, Sherien M. Bakry, Ahmed Zayed, Mohamed A. Farag

**Affiliations:** 1 Pharmacognosy Department, Faculty of Pharmacy, Modern University for Technology and Information, Cairo, Egypt; 2 Plant Systematic Department, National Research Centre (NRC), Giza, Egypt; 3 Pharmacognosy Department, College of Pharmacy, Tanta University, Elguish Street (Medical Campus), Tanta, Egypt; 4 Pharmacognosy Department, College of Pharmacy, Cairo University, Cairo, Egypt

**Keywords:** Biochemistry, Biological techniques, Biotechnology, Chemistry, Microbiology, Plant sciences

## Abstract

Thermal processing and storage of edible oils promote lipid oxidation, generating compounds that may affect nutritional quality and consumer safety. After ingestion, these compounds are further transformed by gut microbiota, altering their chemical fate and biological impact. The current study applied an integrated gas chromatography-mass spectrometry (GC-MS) and ultra-high-performance liquid chromatography–high-resolution mass spectrometry–tandem mass spectrometry (UPLC-HRMS/MS) workflow coupled with feature-based molecular networking (FBMN) and chemometric analysis to simultaneously track primary and secondary metabolites formed during oil oxidation and subsequent gut microbial metabolism in corn, sesame, and sunflower oils. Metabolite profiling enabled the annotation of 89 primary metabolites by GC–MS and 55 secondary metabolites by UPLC-HRMS/MS-FBMN. Gut microbiota incubation markedly reduced several oxidation-related compounds, including 2,4-decadienal (0.05–0.11%), 2,4-nonadienal (0.01–0.25%), *N*-nitrosodiethanolamine (0.03–0.05%), 3,5-diethyl-2-methylpyrazine (0.02–0.24%), oxalic acid (1.2–1.8%), and diethylene glycol (0.2–0.4%), compared with uninoculated controls. In contrast, microbial incubation increased phenol (39–46%) and indole (18.2–22.6%), indicating active microbial metabolism of aromatic amino acids. These findings demonstrate how oxidation-derived oil metabolites are dynamically reshaped by human gut microbiota using a unified multi-platform metabolomics strategy, providing insight into the post-ingestion chemical fate of thermally processed edible oils.

## Introduction

Edible oils are indispensable components of the human diet and are widely used in domestic cooking and the food industry. Among plant-derived oils, corn, sesame, and sunflower oils, obtained from *Zea mays* L., *Sesamum indicum* L., and *Helianthus annuus* L., respectively, are particularly popular because of their high content of unsaturated fatty acids and favorable nutritional properties^1–3^. International dietary guidelines recommend replacing saturated fatty acids with polyunsaturated fatty acids (PUFAs) owing to their beneficial effects on cardiovascular health and lipid metabolism^4,5^. In this context, sunflower oil contains *ca*. 69% PUFAs and 11% saturated fatty acids (SFAs), followed by corn oil (59% PUFAs, 13% SFAs) and sesame oil (41% PUFAs, 15.5% SFAs)^4,6,7^.

Despite their nutritional value, oils rich in unsaturated fatty acids are highly susceptible to oxidation during thermal processing (e.g., frying) and storage, leading to quality deterioration and the formation of numerous oxidation products. Lipid oxidation proceeds through the initial formation of hydroperoxides, i.e., primary oxidation products, which subsequently decompose into aldehydes, ketones, and alkenals, i.e., secondary oxidation products, and finally into smaller carboxylic acids and other low-molecular-weight compounds^5^. The type and abundance of these products depend on multiple factors, including fatty acid composition, temperature, light exposure, presence of pro-oxidant metals or antioxidants, and processing and storage conditions^8^. Many of these oxidation products are responsible not only for undesirable sensory changes but also for potential adverse health effects, which has increased the need for comprehensive chemical characterization of oxidized oils.

After ingestion, dietary lipids and their oxidation products encounter the gut microbiota, a dense and metabolically active microbial ecosystem consisting of more than 10^14^ microorganisms. This microbial community plays a central role in host metabolism, immunity, and nutritional status and is capable of transforming a wide range of dietary and xenobiotic compounds through diverse enzymatic reactions^9^. Increasing evidence shows that dietary lipids and thermally processed oils can modulate gut microbiota composition and activity, and conversely, that gut microbiota can metabolize lipid-derived compounds, potentially altering their biological effects^10–13^. However, only a limited number of omics-based studies have directly addressed how gut microbiota reshapes the chemical composition of foods and their processing-derived products, with most work focusing on digestion processes rather than microbial biotransformation^9,14,15^.

Metabolomics, particularly gas chromatography–mass spectrometry (GC-MS) and liquid chromatography–mass spectrometry (LC-MS)-based platforms, has become a powerful approach for characterizing complex food matrices and microbiome-driven transformations. GC-MS is well established for profiling fatty acids, volatiles, and other primary metabolites in edible oils, whereas ultra-high-performance liquid chromatography–high-resolution mass spectrometry (UHPLC-HRMS) enables the detection of less volatile and more structurally diverse secondary metabolites, including microbial biotransformation products^9,16,17^. Feature-based molecular networking (FBMN) further facilitates the organization and annotation of complex tandem mass spectrometry (MS/MS) datasets, providing a systems-level view of chemical diversity^18^.

The present work is based on previous studies addressing both the prevention of lipid oxidation and the biological consequences of consuming oxidized oils. The effectiveness of natural antioxidants, including nanoencapsulated *Jania rubens* extract, in retarding vegetable oil rancidity has been previously demonstrated^19^, and more recently, thermally oxidized oils were shown to induce significant hepatic injury in vivo, which could be mitigated by dietary intervention using a lemon–ginger blend^17^. While these studies established both the chemical deterioration of oils during thermal processing and their adverse biological effects, the metabolic fate of oxidized oils after ingestion, particularly their transformation by the gut microbiota, has remained largely unexplored. In this context, corn, sesame, and sunflower oils represent an excellent case study due to their widespread consumption and frequent exposure to thermal processing in everyday diets. Despite their importance, the impact of gut microbiota on the chemical fate of fresh and oxidized edible oils is still poorly understood. Therefore, the present study was designed to characterize the metabolite changes induced by thermal oxidation of these oils and to investigate how human gut microbiota, using ex vivo cultures, further modulates the metabolite profiles of both fresh and oxidized samples. To address these objectives, an integrated analytical strategy combining GC-MS and UPLC-HRMS/MS-based FBMN with chemometric analysis was employed, providing a comprehensive and system-level view of oil-microbiota chemical interactions.

## Results

### Oils’ rancidity determination *via* peroxide value (PV)

The PVs of all fresh and oxidized corn, sesame, and sunflower oil samples are presented in Supplementary Table S1. Oxidized samples exhibited markedly higher PVs compared with their corresponding fresh counterparts, confirming the successful induction of lipid oxidation and validating their use for subsequent metabolomic analyses.

### Metabolite profiling of fresh and oxidized oil samples *via* GC-MS

Visual inspection of the chromatograms (Supplementary Fig. S1) revealed clear qualitative and quantitative differences between fresh and oxidized samples, with the most pronounced changes observed in sunflower oil.

In total, 79 metabolites were detected across all samples (Table 1), belonging to several chemical classes, including organic acids, alcohols, aldehydes, esters, fatty acids and fatty acid esters, nitrogen-containing compounds, phenols, sterols, and tocopherols. The relative contributions of the different metabolite classes are summarized in Table 1 and Supplemenary Fig. S2, and a representative chromatogram highlighting their elution regions is shown in Supplementary Fig. S1. The main compositional changes induced by oxidation are demonstrated below by metabolite class.

**Table 1:** Levels of silylated primary metabolites in COO, COF, SOO, SOF, SFO, and SFF. Oils were analyzed *via* GC-MS. Results are expressed as relative percentages of the total peak areas

Peak no.	Average Rt (min)	Name	Class	COO	COF	Fold ratio	SOO	SOF	Fold ratio	SFO	SFF	Fold ratio
2	4.749	4-Methylvaleric acid	Acid	0.35	0.66	0.53	0.47	0.28	1.68	0.65	0.61	1.08
6	5.128	Lactic acid		1.83	1.79	1.03	1.49	2.22	0.67	4.11	1.06	3.89
7	5.143	*α*-Ketovaleric acid		2.17	3.82	0.57	2.53	0.93	2.73	3.35	3.06	1.10
8	5.248	Caproic acid		0.46	0.16	2.87	0.23	0.19	1.20	0.22	0.16	1.37
9	5.335	Glycolic acid		0.38	0.44	0.85	0.55	1.04	0.53	0.61	0.67	0.91
10	5.477	2-Ketoisocaproic acid		0.01	0.10	0.07	0.15	0.12	1.24	0.01	0.08	0.10
11	5.523	Pyruvic acid, enol		0.21	0.11	1.90	0.18	0.10	1.91	0.04	0.04	0.90
12	5.644	2-Ethyl hexanoic acid		0.23	0.41	0.56	0.37	1.22	0.30	0.66	0.99	0.66
13	5.757	Oxalic acid		8.22	15.32	0.54	10.33	5.55	1.86	12.61	13.02	0.97
14	6.164	Heptanoic acid		0.12	0.31	0.38	0.14	0.15	0.94	0.10	0.13	0.75
16	6.349	Beta-Lactic acid		0.39	0.69	0.57	0.32	0.04	7.03	0.63	0.09	6.71
17	6.364	Beta-Lactic acid		0.35	0.51	0.67	0.33	0.58	0.57	0.78	0.44	1.78
18	6.45	Beta-amino isobutyric acid		0.00	0.00	0.00	0.00	0.01	0.00	0.01	0.00	0.00
21	7.074	3-Hydroxyisovaleric acid		0.03	0.07	0.40	0.04	0.03	1.59	0.13	0.06	2.01
25	7.744	2-Hydroxyisocaproic acid		0.00	0.00	0.00	0.00	0.00	0.00	0.00	0.00	0.00
26	7.812	2-Hydroxy-3-methylvaleric acid		0.00	0.00	0.00	0.00	0.00	0.00	0.00	0.00	0.00
28	7.859	Benzoic acid		0.05	0.04	1.07	0.01	0.05	0.16	0.02	0.01	1.67
29	8.057	Octanoic acid		2.89	4.23	0.68	2.77	0.88	3.15	3.95	2.95	1.34
33	8.362	Phosphoric acid		0.00	0.00	0.00	0.00	0.24	0.00	0.00	0.09	0.00
37	8.573	Phenylacetic acid		0.14	0.32	0.43	0.33	0.08	4.17	0.17	0.39	0.44
39	8.806	Succinic acid		0.44	0.73	0.61	0.81	2.47	0.33	0.71	1.58	0.45
43	9.13	Glyceric acid		0.60	0.72	0.83	2.22	2.76	0.80	0.70	2.50	0.28
45	9.388	Nonanoic acid		0.13	0.23	0.55	0.76	13.17	0.06	0.46	8.03	0.06
48	10.038	Glutaric acid		0.01	0.00	0.00	0.00	0.005	0.00	0.00	0.02	0.00
50	10.183	Hydrocinnamic acid		0.00	0.00	0.00	0.01	0.01	1.15	0.00	0.01	0.00
52	10.67	Decanoic acid		0.00	0.00	0.00	0.00	0.00	0.00	0.00	0.00	0.00
61	12.985	3-Hydroxyphenylacetic acid		0.40	0.44	0.92	0.71	0.56	1.27	0.76	0.87	0.88
64	14.279	4-Hydroxyphenyl propionic acid		0.00	0.03	0.00	0.00	0.00	0.00	0.00	0.00	0.00
65	14.574	Vanillic acid		0.01	0.00	0.00	0.01	0.00	0.00	0.00	0.00	0.00
66	14.636	Azelaic acid/ Nonanedioic acid		0.61	1.03	0.59	0.36	0.18	2.07	0.38	0.27	1.42
**Total Acids**				**20.00**	**32.17**	**0.62**	**25.12**	**32.84**	**0.76**	**31.06**	**37.14**	**0.84**
1	4.247	Propylene glycol	Alcohol	0.13	1.38	0.09	0.22	0.39	0.56	0.26	1.20	0.21
3	4.855	Propylene glycol		0.05	0.06	0.78	0.03	0.03	1.21	0.05	0.07	0.81
5	5.043	1,3-Propanediol		3.04	5.58	0.54	3.69	1.21	3.04	4.55	4.64	0.98
15	6.218	Nonanol		0.00	0.00	0.00	0.22	1.85	0.12	0.06	1.06	0.05
19	6.491	Benzyl alcohol		0.00	0.00	0.00	0.00	0.06	0.00	0.00	0.00	0.00
20	6.632	1,4-Butanediol		0.38	0.61	0.63	0.46	0.28	1.67	0.65	0.61	1.06
23	7.565	2-Phenylethanol		0.00	0.00	0.00	0.00	0.03	0.00	0.00	0.00	0.00
27	7.835	Diethylene glycol		0.03	0.00	0.00	0.11	1.12	0.10	0.02	0.72	0.03
32	8.338	Glycerol		0.24	0.79	0.30	1.08	0.62	1.73	0.33	1.26	0.26
60	12.951	3-Hydroxy-4-methoxybenzyl alcohol		0.00	0.00	0.00	0.00	0.00	0.00	0.00	0.00	0.00
74	16.584	1-Hexadecanol		0.00	0.00	0.00	0.00	0.00	0.00	0.00	0.00	0.00
78	18.106	1-Octadecanol		0.00	0.00	0.00	0.00	0.00	0.00	0.00	0.00	0.00
**Total Alcohols**				**3.86**	**8.42**	**0.46**	**5.81**	**5.60**	**1.04**	**5.92**	**9.56**	**0.62**
35	8.493	2,4-Nonadienal	Aldehyde	0.45	0.71	0.64	0.75	0.37	2.01	0.66	0.27	2.43
40	8.812	2,4-Decadienal		0.88	1.28	0.69	1.52	0.85	1.80	1.27	0.72	1.76
57	11.705	3-Hydroxy-4-methoxybenzaldehyde		0.00	0.00	0.00	0.00	0.00	0.00	0.00	0.00	0.00
69	15.607	7,10-Hexadecadienal		0.96	0.55	1.75	0.88	0.42	2.11	1.21	0.97	1.25
70	15.663	Octadecenal		3.86	1.14	3.39	2.46	0.78	3.14	2.97	1.26	2.37
**Total Aldehydes**				**6.16**	**3.67**	**1.68**	**5.61**	**2.42**	**2.32**	**6.12**	**3.22**	**1.90**
34	8.47	Butyric acid, benzyl ester	Ester	0.99	0.81	1.22	0.81	0.43	1.90	1.02	0.66	1.53
46	9.483	3-Methyl benzoate		0.00	0.00	0.00	0.00	0.00	0.00	0.00	0.01	0.00
55	11.497	4-Methoxybenzoate		0.01	0.03	0.40	0.08	0.21	0.37	0.06	0.14	0.47
**Total Esters**				**1.00**	**0.84**	**1.19**	**0.89**	**0.63**	**1.40**	**1.08**	**0.81**	**1.33**
62	13.004	Lauric acid	Fatty acid/ Ester	0.16	0.10	1.56	0.22	0.20	1.07	0.18	0.18	0.99
67	15.13	Myristic acid		0.06	0.14	0.39	0.12	0.38	0.32	0.11	0.29	0.37
72	15.841	n-Pentadecanoic acid		0.00	0.00	0.00	0.00	0.00	0.00	0.00	0.01	0.00
75	17.065	Palmitic acid		12.73	6.71	1.90	8.29	8.38	0.99	5.42	5.49	0.99
77	17.973	Margaric acid		0.10	0.16	0.63	0.19	0.37	0.52	0.11	0.17	0.68
79	18.603	Linoleic acid		15.34	11.79	1.30	14.33	12.15	1.18	12.87	11.78	1.09
80	18.633	Oleic acid		26.86	15.26	1.76	18.47	14.96	1.23	17.52	11.67	1.50
81	18.844	Stearic acid		2.47	2.43	1.01	4.35	8.99	0.48	2.90	5.60	0.52
82	19.371	Linoleic acid		1.00	1.61	0.62	1.12	0.33	3.39	1.47	0.75	1.97
83	20.362	Glyceryl linoleate		0.81	0.78	1.04	1.17	0.78	1.51	1.21	1.16	1.05
84	20.492	Arachidic acid		0.47	0.66	0.71	0.78	0.43	1.79	1.01	0.60	1.69
85	20.752	9, 12-Octadecadienoic acid, methyl ester/Linoleic acid		3.03	1.62	1.88	2.75	0.74	3.71	3.19	1.70	1.88
86	21.713	1-Monopalmitin/ Glyceryl-1-palmitate		0.34	0.23	1.50	1.12	1.23	0.91	0.45	1.42	0.31
87	22.014	Docosanoic acid		0.12	0.17	0.67	0.30	0.10	3.14	0.24	0.16	1.49
89	23.235	Sebacic acid, di-(2-ethylhexyl) ester/ Decanedioic acid, di-(2-ethylhexyl) ester		0.05	0.00	0.00	0.08	1.09	0.07	0.09	0.51	0.17
**Total Fatty acids/Esters**				**63.53**	**41.69**	**1.52**	**53.31**	**50.15**	**1.06**	**46.78**	**41.48**	**1.13**
22	7.463	Valine	Nitrogenous/Amino acid	0.01	0.02	0.36	0.23	0.00	0.00	0.23	0.04	5.25
24	7.705	Ethanolamine		3.11	5.47	0.57	3.61	0.95	3.82	4.84	3.91	1.24
30	8.178	Nicotinic acid/ Vitamin B3		0.02	0.12	0.18	0.07	0.34	0.21	0.05	0.28	0.19
31	8.274	L-Leucine		0.00	0.03	0.00	0.13	0.00	0.00	0.00	0.00	0.00
36	8.493	Nicotinic acid/ Vitamin B3		0.01	0.08	0.18	0.00	0.02	0.00	0.00	0.00	0.00
38	8.585	Isoleucine		0.02	0.01	2.06	0.08	0.005	18.03	0.00	0.00	0.00
42	8.917	Nicotinic acid/ Vitamin B3		0.00	0.01	0.00	0.02	0.10	0.18	0.00	0.03	0.00
44	9.197	Uracil		0.00	0.00	0.00	0.00	0.00	0.00	0.00	0.00	0.00
49	10.055	Thymine		0.00	0.00	0.00	0.00	0.00	0.00	0.00	0.00	0.00
51	10.519	Indole		0.00	0.00	0.00	0.00	0.00	0.00	0.02	0.00	0.00
53	10.793	*N*-Nitroso-diethanolamine		0.02	0.01	2.06	0.01	0.00	0.00	0.00	0.00	0.00
54	11.011	L-Aspartic acid		0.01	0.00	0.00	0.00	0.00	0.00	0.00	0.00	0.00
56	11.58	Pyroglutamic acid		0.04	0.14	0.29	0.33	0.06	5.05	0.04	0.11	0.35
58	12.762	Glutamic acid		0.00	0.16	0.00	0.00	0.00	0.00	0.00	0.00	0.00
59	12.842	Phenylalanine		0.00	0.00	0.00	0.00	0.00	0.00	0.00	0.00	0.00
63	13.778	Indole, 5-hydroxy		0.00	0.00	0.00	0.00	0.00	0.00	0.00	0.00	0.00
68	15.445	Adenine		0.00	0.00	0.00	0.00	0.00	0.00	0.00	0.00	0.00
71	15.806	Vitamin B6/ Pyridoxine		0.08	0.09	0.88	0.06	0.04	1.55	0.16	0.04	4.53
73	16.424	Indole-3-acetic acid		0.00	0.00	0.00	0.01	0.00	0.00	0.00	0.00	0.00
76	17.545	Indole-3-propionic acid		0.00	0.00	0.00	0.00	0.00	0.00	0.00	0.00	0.00
88	22.22	Adenosine		0.00	0.00	0.00	0.01	0.00	0.00	0.01	0.01	0.60
92	26.065	3,5-Diethyl-2-methylpyrazine		0.31	0.70	0.44	1.74	1.77	0.98	0.33	0.35	0.96
**Total Nitrogenous/ Amino acids**				**3.63**	**6.84**	**0.53**	**6.29**	**3.28**	**1.92**	**5.69**	**4.77**	**1.19**
4	5.002	Phenol	Phenols	0.31	0.59	0.52	0.39	0.24	1.63	0.33	0.53	0.62
41	8.899	Pyrocatechol		0.09	0.14	0.66	0.07	0.08	0.93	0.18	0.09	1.96
**Total Phenols**				**0.40**	**0.73**	**0.55**	**0.46**	**0.31**	**1.46**	**0.51**	**0.62**	**0.82**
47	10.005	Hydroquinone	Quinones	0.00	0.00	0.00	0.00	0.00	0.00	0.00	0.00	0.00
**Total Quinones**				**0.00**	**0.00**	**0.00**	**0.00**	**0.00**	**0.00**	**0.00**	**0.00**	**0.00**
93	27.184	Campesterol	Sterols	0.42	0.82	0.52	0.64	0.58	1.11	0.58	0.64	0.91
94	28.206	*β*-Sitosterol		0.99	3.03	0.33	1.32	1.83	0.72	2.26	1.69	1.34
**Total Sterols**				**1.41**	**3.84**	**0.37**	**1.96**	**2.41**	**0.81**	**2.84**	**2.33**	**1.22**
90	23.977	*δ*-Tocopherol	Vitamins	0.00	0.00	0.00	0.18	0.34	0.52	0.00	0.00	0.00
91	24.69	Tocopherol		0.00	1.79	0.00	0.38	2.02	0.19	0.00	0.06	0.00
**Total Vitamins**				**0.00**	**1.79**	**0.00**	**0.56**	**2.37**	**0.24**	**0.00**	**0.06**	**0.00**

For codes explanation, refer to Table 2. Data represents relative semi-quantitative metabolite abundances obtained from a single experimental run; therefore, values are descriptive only and no statistical analysis or significance testing was performed. The table is intended to illustrate comparative trends in metabolite distribution among samples rather than infer population-level differences.

Fatty acids and fatty acid esters constituted the dominant metabolite class in both fresh and oxidized samples of all three oils (Table 1). The relative abundance of this class was higher in oxidized corn (COO), sesame (SOO), and sunflower (SFO) oils (64%, 53%, and 47%, respectively) compared with their corresponding fresh samples (COF, SOF, and SFF; 42%, 50%, and 42%, respectively).

Oleic acid (ω-9) and linoleic acid (ω-6) represented the major unsaturated fatty acids in both fresh and oxidized oils, followed by the saturated fatty acids palmitic and stearic acids. Interestingly, oxidized oils showed higher relative levels of free oleic and linoleic acids compared with fresh samples. In addition, minor levels of glycidyl fatty acid esters, including glyceryl linoleate and 1-monopalmitin, were detected in both fresh and oxidized samples (Table 1).

Organic acids represented the second most abundant metabolite class in all oil samples (Table 1). In contrast to fatty acids, their overall relative abundance was lower in oxidized oils compared with fresh samples. Fresh corn, sesame, and sunflower oils contained higher relative levels of organic acids (32%, 33%, and 37%, respectively) than their oxidized counterparts (20%, 25%, and 31%, respectively).

Several organic acids were consistently detected across samples, including nonanoic acid, oxalic acid, lactic acid, octanoic acid, glyceric acid, and succinic acid. Interestingly, fresh sesame and sunflower oils showed relatively higher levels of nonanoic acid compared with their oxidized counterparts. Conversely, several short- and medium-chain fatty acids, including 4-methylvaleric acid, caproic acid, and octanoic acid, were relatively more abundant in oxidized oils. In addition, oxalic acid was detected at relatively high levels in both fresh and oxidized oils, while phosphoric acid was observed at trace levels in some samples.

In the present dataset, nitrogen-containing and amino acid–related metabolites collectively accounted for 3.3–6.8% of the total detected metabolites across fresh and oxidized corn, sesame, and sunflower oils (Table 1), indicating their presence mainly as trace-level components or processing-related residues rather than intrinsic oil constituents.

Among these compounds, ethanolamine was detected as a major representative in several samples, particularly in oxidized sesame (SOO) and sunflower (SFO) oils, where it reached 3.6% and 4.8%, respectively, compared with lower levels in the corresponding fresh oils. In contrast, oxidized corn oil (COO) showed slightly lower ethanolamine levels than its fresh counterpart.

The potentially harmful contaminant *N*-nitrosodiethanolamine was detected at trace levels in both fresh and oxidized samples (Table 1). In addition, 3,5-diethyl-2-methylpyrazine was detected at comparable but slightly higher levels in oxidized oils compared with fresh ones.

Moreover, indole derivatives were detected only at trace levels, primarily in oxidized sunflower oil, in agreement with previous reports describing their sporadic presence in corn and sesame oils^20^. Finally, nicotinic acid (vitamin B3) was detected at low levels in fresh oils and showed a marked decrease in oxidized samples.

Aldehydes comprise a chemically diverse group, including alkadienals (e.g., 2,4-decadienal, 2,4-nonadienal), alkenals, and aromatic aldehydes. Oxidized oils (COO, SOO, and SFO) exhibited higher relative levels of aldehydes (6.0%, 5.6%, and 6.1%, respectively) compared with their fresh counterparts (3.7%, 2.4%, and 3.2%, respectively), corresponding to approximately twofold increases (Table 1). This trend is consistent with previous reports on thermally oxidized sunflower and other vegetable oils^14^.

Among the detected compounds, 2,4-decadienal and 2,4-nonadienal showed the most pronounced increases in oxidized sesame and sunflower oils, whereas corn oil displayed a less consistent pattern. These alkadienals are well-established degradation products of linoleic acid and are among the most characteristic volatile markers of lipid oxidation in vegetable oils^21^. The generally higher abundance of 2,4-decadienal relative to 2,4-nonadienal agrees with previous studies on stored and heated sunflower oils, where alkadienals were reported as dominant oxidation products^22–24^. 2,4-Decadienal is a potentially cytotoxic and harmful mutation biomarker^25,26^

Furthermore, octadecenal was also consistently elevated in oxidized samples, showing about three- to four-fold higher levels compared with fresh oils, further supporting the occurrence of advanced lipid oxidation processes.

In the present study, only trace levels of simple phenolics, mainly phenol (peak 4) and pyrocatechol (peak 41), were detected in both fresh and oxidized oils (0.31–0.73%).

Alcohols are also commonly classified as secondary lipid oxidation products formed through the degradation of hydroperoxides and other primary oxidation intermediates^1,27^. In the present dataset, alcohols were detected in both fresh and oxidized oils at variable levels (3.9–9.6%), without a uniform increase upon oxidation. 1,3-Propanediol (peak 5) was the most abundant alcohol detected in all samples. In addition, glycerol (peak 32) was also consistently detected at low levels.

Moreover, nonanol (peak 15) was more abundant in fresh sesame and sunflower oils and decreased upon oxidation, consistent with its further degradation or volatilization during prolonged heating. Interestingly, the toxic contaminant diethylene glycol (peak 27) was detected at relatively high levels in fresh sesame oil and showed a decrease after oxidation.

Phytosterols, represented mainly by campesterol (peak 93) and *β*-sitosterol (peak 94), were detected at low but consistent levels (1.4–3.8%) in both fresh and oxidized oils, with corn and sesame oils showing relatively higher abundances, in agreement with previous reports^1,28^.

Esters were detected at low and relatively constant levels across all samples (0.63–1.0%), mainly represented by butyric acid benzyl ester (peak 34). Finally, Tocopherols were clearly more abundant in fresh corn and sesame oils (1.8% and 2.4%, respectively) and were detected only at trace levels in their corresponding oxidized samples.

### Metabolite profiling of fresh and oxidized oil (inoculated and uninoculated with human gut microbiota ex vivo culture) *via* GC-MS

GC-MS analysis was further applied in fresh and oxidized corn, sesame, and sunflower oils following incubation with human gut microbiota using an ex vivo culture model (Supplementary Table S2).

Across all inoculated and uninoculated samples, a total of 89 metabolites were annotated (Supplementary Table S2), belonging to several chemical classes, including 30 acids, 12 alcohols, 5 aldehydes, 3 esters, 15 fatty acids/esters, 22 nitrogen-containing compounds/amino acids, 2 phenols, 1 quinone, and 2 sterols. Distinctly, approx. 25 metabolites were detected exclusively in microbiota incubation experiments and were absent from the direct analysis of fresh and oxidized oils. These included, among others, 2-hydroxyisocaproic acid (peak 25), 2-hydroxy-3-methylvaleric acid (peak 26), decanoic acid (peak 52), vanillic acid (peak 65), 3-hydroxy-4-methoxybenzyl alcohol (peak 60), 1-hexadecanol (peak 74), 1-octadecanol (peak 78), 3-hydroxy-4-methoxybenzaldehyde (peak 57), uracil (peak 44), thymine (peak 49), phenylalanine (peak 59), 5-hydroxyindole (peak 63), adenine (peak 68), indole-3-propionic acid (peak 76), and hydroquinone (peak 47). The relative distributions of all metabolite classes are summarized in Supplementary Fig. S3. The major class-specific changes observed upon microbial incubation are discussed below.

Organic acids represented the most abundant metabolite class in both inoculated and uninoculated oil samples (Supplementary Table S2). However, microbial incubation was associated with a pronounced decrease in their overall relative abundance, dropping to 22.7–25.6% compared with 44.7–48.9% in uninoculated controls.

Several short-chain and medium-chain fatty acids showed marked decreases upon incubation, including lactic acid (0.46–0.78% in inoculated *vs*. 6.74–11.4% in uninoculated samples) and caprylic acid (0.22–0.55% *vs*. 1.19–1.32%, respectively). In contrast, 4-methylvaleric acid showed a pronounced increase in inoculated samples (11.8–14.4%) compared with trace levels in uninoculated controls (0.5–1.4%). Importantly, oxalic acid (peak 13) showed a marked decrease following microbial incubation (1.2–1.8%) compared with uninoculated samples (5.4–6.8%).

Fatty acids and fatty acid esters represented the second most abundant metabolite class in both inoculated and uninoculated samples (Supplementary Table S2). Interestingly, microbial incubation was associated with a potential reduction in their overall relative abundance, decreasing to 4.0–6.4% compared with 25–34% in uninoculated controls.

Additionally, microbial incubation led to a substantial decrease in glycidyl fatty acid esters, including glyceryl linoleate (0.03–0.1% in inoculated *vs*. 0.17–0.49% in uninoculated samples) and glyceryl-1-palmitate (0.09–0.21% *vs*. 0.44–0.51%, respectively).

Nitrogen-containing metabolites and amino acid–related compounds accounted for a substantially higher proportion of the total metabolite pool in inoculated samples (22–28%) compared with uninoculated controls (9–15%). In addition, branched-chain amino acids, including valine, leucine, and isoleucine, were consistently detected at higher relative levels in inoculated samples (0.01–0.20%) compared with uninoculated oils (0.16–0.66%) (Supplementary Table S2).

Microbial incubation was associated with a pronounced decrease in *N*-nitrosodiethanolamine (peak 53), which declined to 0.03–0.05% compared with 0.08–0.19% in uninoculated samples. Similarly, a marked reduction was observed for 3,5-diethyl-2-methylpyrazine (0.02–0.24%) and ethanolamine (0.15–0.34%) relative to uninoculated samples. In contrast, indole (peak 51) accumulated to high relative levels in inoculated samples (18.2–22.6%) but remained at only trace levels in uninoculated controls (0.01–1.21%). In addition to indole itself, related metabolites such as indole-3-acetic acid (peak 73) and indole-3-propionic acid (peak 76) were also detected, further supporting active microbial tryptophan metabolism during incubation.

Microbial incubation resulted in a pronounced reduction in aldehydes, which are hallmark secondary lipid oxidation products. In inoculated samples, total aldehydes decreased to 0.09–0.39% compared with 0.60–1.99% in uninoculated oils. This decrease included key lipid oxidation markers such as 2,4-decadienal (peak 40) and 2,4-nonadienal (peak 35).

This observation indicates that these reactive carbonyl compounds undergo microbial transformation or degradation under the applied ex vivo conditions. The strongest reduction was observed in corn oil fresh incubated samples (COFI), where aldehydes dropped to about 0.09%.

Phenolic compounds were dominated by phenol (peak 4), which accumulated to very high relative levels in all inoculated samples (38.6–45.9%) but was present only at trace levels in uninoculated controls (0.19–1.45%).

Alcohols were more abundant in uninoculated samples (7.22–10.09%) than in inoculated samples (2.45–4.23%), indicating net microbial consumption or transformation of several alcohol species.

Glycerol (peak 32) showed a particularly strong decrease, dropping from 1.03–1.42% in uninoculated samples to 0.07–0.11% after incubation. Concomitantly, 1,3-propanediol (peak 5) accumulated in inoculated samples. This compound is a known microbial metabolite formed from glycerol *via* 3-hydroxypropionaldehyde as an intermediate in species such as *Lactobacillus* and *Klebsiella*^29^, providing a coherent biochemical explanation for these reciprocal trends.

A pronounced decrease was also observed for diethylene glycol (peak 27), which declined from 1.15–1.34% in uninoculated samples to 0.21–0.40% after incubation.

Phytosterols, represented mainly by *β*-sitosterol (peak 94) and campesterol (peak 93), showed a moderate decrease upon microbial incubation, declining from 0.61–1.18% in uninoculated samples to 0.14–0.75% in inoculated samples.

### Multivariate data analyses of GC–MS dataset

**Fig. 1:**
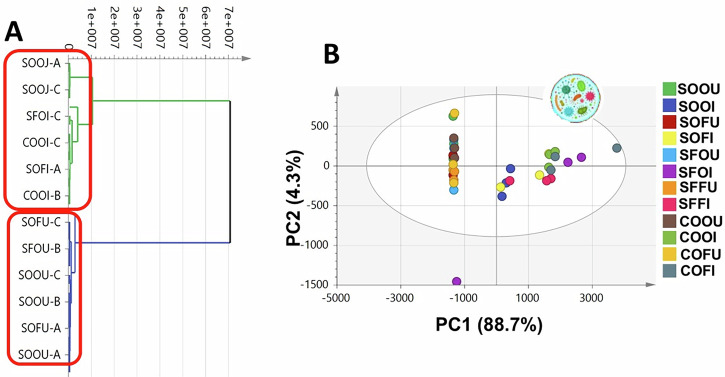
GC-MS based HCA and PCA of all oils’ samples. **A** HCA plot. **B** Score plot of PC1 *vs*. PC2 scores. No pattern of clustering in two-dimensional space described by two vectors of PC1 = 88.7% and PC2 = 4.3%. (Oxidized uninoculated sesame oil (SOOU), oxidized inoculated sesame oil (SOOI), fresh uninoculated sesame oil (SOFU), fresh inoculated sesame oil (SOFI), oxidized uninoculated sunflower oil (SFOU), oxidized inoculated sunflower oil (SFOI), fresh uninoculated sunflower oil (SFFU), fresh inoculated sunflower oil (SFFI), oxidized uninoculated corn oil (COOU), oxidized inoculated corn oil (COOI), fresh uninoculated corn oil (COFU), and fresh inoculated corn oil (COFI) samples. For codes explanation, refer to Table 2).

Hierarchical clustering analysis (HCA) (Fig. 1A) revealed a primary separation between inoculated and uninoculated samples. However, this model did not clearly resolve differences among oil types or between fresh and oxidized samples. Similarly, principal component analysis (PCA) of the full dataset (Fig. 1B), explaining 93% of the total variance, did not yield a clear separation pattern among all experimental groups. Therefore, subsequent unsupervised analyses were performed separately for each oil type to resolve treatment effects.

For corn oil samples, PCA (Supplementary Fig. S4A) explained 94.7% of the total variance (PC1 = 92.3%). Uninoculated fresh and oxidized samples (COFU and COOU) clustered on the left side of the score plot, whereas inoculated samples (COFI and COOI) clustered together on the right side, with substantial overlap between fresh and oxidized inoculated samples. The corresponding loading plot (Supplementary Fig. S4B) indicated that phenylacetic acid, 4-methylvaleric acid, propylene glycol, indole, and phenol contributed most strongly to the separation of inoculated from uninoculated samples.

A similar pattern was observed for sesame oil samples. PCA (Supplementary Fig. S5A) explained 93.7% of the total variance, with inoculated samples (SOFI and SOOI) clustering on the right side of the score plot and uninoculated samples (SOFU and SOOU) on the left. Notably, sesame oil was the only case in which fresh and oxidized uninoculated samples showed partial separation. The loading plot (Supplementary Fig. S5B) revealed that 4-methylvaleric acid, 1,3-propanediol, propylene glycol, indole, uracil, and phenol were the main contributors to the discrimination of inoculated samples.

For sunflower oil, PCA (Supplementary Fig. S6A) showed separation of uninoculated fresh and oxidized samples (SFFU and SFOU) on the left side, while inoculated samples (SFFI and most SFOI replicates) clustered on the right side. The loading plot (Supplementary Fig. S6B) again indicated that 4-methylvaleric acid, propylene glycol, indole, and phenol were major contributors to the positioning of inoculated samples.

For uninoculated corn oil samples, the supervised orthogonal partial least squares discriminant analysis (OPLS-DA) model (Supplementary Fig. S7A) showed excellent model performance (R^2^ = 0.99, Q^2^ = 0.94) and achieved clear separation between fresh (COFU) and oxidized (COOU) samples. The corresponding S-loading plot (Supplementary Fig. S7B) indicated that benzoic acid, linoleic acid, palmitic acid, and indole were associated with oxidized samples, whereas lactic acid and nicotinic acid were more characteristic of fresh samples.

For inoculated corn oil samples, the OPLS-DA model (Supplementary Fig. S8A) also showed clear discrimination between COFI and COOI. The S-loading plot (Supplementary Fig. S8B) revealed that 4-methylvaleric acid, phenol, and indole were the main metabolites driving the separation, reflecting dominant microbial fermentation signatures.

In the case of uninoculated sesame oil, OPLS-DA (Supplementary Fig. S9A) yielded a robust model (R^2^ = 98.3%, Q^2^ = 92.4%) with clear separation between SOFU and SOOU samples. The loading plot (Supplementary Fig. S9B) indicated that nonanoic, palmitic, stearic, and oleic acids were more abundant in oxidized samples.

Similarly, OPLS-DA of inoculated sesame oil samples (Supplementary Fig. S10A) showed excellent model performance (R^2^ = 99.9%, Q^2^ = 99.8%) and clear discrimination between SOFI and SOOI. According to the loading plot (Supplementary Fig. S10B), 4-methylvaleric acid, indole, uracil, and phenol were more abundant in fresh inoculated samples and were the main contributors to group separation.

For sunflower oil, no statistically valid OPLS-DA model could be obtained due to negative predictive power, indicating insufficient systematic variation between fresh and oxidized samples under the studied conditions.

### Metabolites profiling of fresh and oxidized oil samples *via* UPLC‑HRMS/MS

**Fig. 2:**
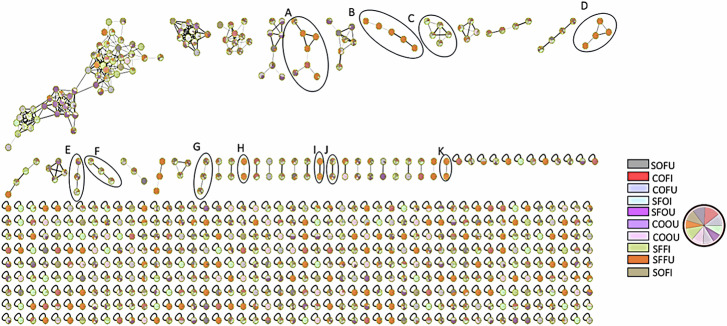
FBMN was created with MS/MS data in positive ionization mode. The network shows nodes as pie charts, reflecting the relative abundance of each ion in each oil sample.

Using an acetonitrile–acidified water gradient, chromatographic separation was achieved within 17 min (Supplementary Fig. S11A–C). The resulting network comprised 803 features, of which 192 were organized into 39 molecular clusters, while 611 features appeared as self-loop nodes (Fig. 2).

A total of 55 secondary metabolites were annotated, belonging to diverse chemical classes, including 33 peptides, 10 indole alkaloids, 3 sphingolipids, 6 fatty acids, 1 alanine derivative, 1 monoacylglycerol, and 1 nucleoside. Interestingly, most peptide and indole-derived metabolites were detected almost exclusively in sunflower oil samples, particularly in fresh and gut-treated conditions.

Peptides constituted the largest annotated chemical family (33 compounds) in the FBMN. They included dipeptides, tripeptides, polypeptides, and cyclopeptides. Their confident annotation relied on well-established peptide MS/MS fragmentation behavior, which involves characteristic cleavages at both *N*- and *C*-termini, neutral losses of H_2_O + CO (−46 Da), NH_3_ (−17 Da), or combinations thereof, as well as formation of diagnostic immonium ions corresponding to constituent amino acids^30^. The observed immonium ions and proposed peptide sequences are summarized in Supplementary Table S3. None of these peptides was detected by GC–MS (Supplementary Table S3).

Fourteen dipeptides were annotated, most of which were detected predominantly in fresh sunflower oil samples, indicating either endogenous seed peptides or mild proteolysis products released during oil processing. Peaks 2, 10, 12, and 13 clustered together (cluster B) and were specific to fresh sunflower oil. Serinyl-phenylalanine (11, *m/z* 253.1180) (Supplementary Fig. S12), glycyl-phenylalanine (12, *m/z* 223.1070), and alanyl-phenylalanine (13, *m/z* 237.1230) shared characteristic fragment ions at *m/z* 166 and 120, consistent with phenylalanine-containing dipeptides^31^. Similarly, phenylalanyl-proline (22, *m/z* 263.1395) and phenylalanyl-leucine (26, *m/z* 279.1699) were connected in cluster A, sharing the *m/z* 120 phenylalanine immonium ion. Additional self-loop dipeptides included Val-Val (1, [M + H]^+^ at *m/z* 217.1544), Asn-Gln (3, [M + H]^+^ at *m/z* 261.1189), Leu-Glu (4, [M + H]^+^ at *m/z* 260.1600), Ala-Leu (7, [M + H]^+^ at *m/z* 203.1388), Glu-Leu (8, [M + H]+ at *m/z* 261.1447), Pro-Pro (9, [M + H]+ at *m/z* 213.1228), Val-Leu (14, [M + H]+ at *m/z* 231.1700), and Leu-Leu (20, [M + H]+ at *m/z* 245.1854) **(**Supplementary Fig. S13).

Twelve tripeptides and polypeptides were annotated, all detected exclusively in fresh sunflower oil samples, represented in clusters D, H, K, and four self-loop nodes (Supplementary Fig. S14). In cluster D, peak 15, annotated as tyrosyl-valyl-proline ([M + H]^+^ at *m/z* 378.2017), and peak 29, annotated as tyrosyl-isoleucyl-prolyl-valine ([M + H]^+^ at *m/z* 491.2858), were connected based on their shared fragment at *m/z* 136 corresponding to the tyrosine immonium ion (Supplementary Fig. S15). Peak 29 was linked to peak 30, annotated as leucyl-prolyl-phenylalanine ([M + H]^+^ at *m/z* 376.2258), and peak 33, annotated as glycyl-prolyl-phenylalanyl-prolyl-isoleucine ([M + H]^+^ at *m/z* 530.2955), with all three compounds sharing characteristic fragments at *m/z* 229 and 120, indicative of a Pro–Phe backbone and phenylalanine immonium ion^32^. In cluster H, peak 5, annotated as glutamyl-valyl-proline ([M + H]^+^ at *m/z* 344.1820; Supplementary Fig. S16), and peak 16, annotated as valyl-leucyl-proline ([M + H]^+^ at *m/z* 328.2240; Supplementary Fig. S17), shared fragment ions at *m/z* 229 and 116. Cluster K comprised peak 19, annotated as leucyl-prolyl-valyl-prolyl-glutamine ([M + H]^+^ at *m/z* 553.3341), and peak 25, annotated as valyl-leucyl-prolyl-valyl-prolyl-glutamine ([M + H]^+^ at *m/z* 652.4017), sharing a conserved C-terminal Pro–Val–Pro–Gln motif with common y-ion series at *m/z* 440, 310, 244, 197, and 169.

In the present study, cyclopeptides were represented in the molecular network mainly within clusters A and G, while several others appeared as self-looped nodes (Supplementary Fig. S18). Seven cyclic dipeptides were annotated. Cyclo-(Tyr–Tyr) (17, [M + H]^+^ at *m/z* 327.1346) was detected exclusively in COFI and clustered with cyclo-(Tyr–Phe) (31, [M + H]^+^ at *m/z* 311.1365), annotated from COFU, COFI, SFOU, and SOFU. Cyclo-(Pro–Leu) (24, [M + H]^+^ at *m/z* 211.1438; Supplementary Fig. S19) and cyclo-(Leu–Leu) (34, [M + H]^+^ at *m/z* 227.1755) were present in all tested samples and grouped in cluster G. Additional self-loop cyclopeptides included cyclo-(Phe–Pro) (32, [M + H]^+^ at *m/z* 245.1291), cyclo-(Leu–Phe) (35, [M + H]^+^ at *m/z* 261.1579), and cyclo-(Phe–Trp) ([M + H]+ at *m/z* 334.1544; Supplementary Fig. S20).

Several *β*-carboline derivatives were annotated exclusively in fresh and oxidized oils incubated with gut microbiota, organized in FBMN clusters C, F, and I 72 (Supplementary Fig. S21). In cluster C, 3-phenyl-9H-pyrido[3,4-b]indole (40, [M + H]^+^ at *m/z* 245.1072) clustered with 1-(4-methylphenyl)-9H-pyrido[3,4-b]indole (44, 45, [M + H]^+^ at *m/z* 259.1227) (Supplementary Fig. S22, S23). In cluster F, 2-phenyl-1-(9H-pyrido[3,4-b]indol-1-yl)ethenone (42, 43, [M + H]^+^ at *m/z* 287.1181) was annotated (Supplementary Fig. S24). The self-loop node 1-methyl-*N*-phenyl-9H-pyrido[3,4-b]indole-3-carboxamide (37, [M + H]^+^ at *m/z* 302.1286) appeared only in inoculated sunflower oil (Supplementary Fig. S25). Tetrahydroharman-3-carboxylic acid (21, 23, [M + H]+ at m/z 231.1127) was detected in fresh sunflower oil and grouped in cluster I (Supplementary Fig. S26).

Several fatty acids were detected mainly in the late-elution region, including linoleic acid (49, [M + H]^+^ at *m/z* 281.2467) and oleic acid (51, [M + H]^+^ at *m/z* 283.2626). Fatty acid amides included palmitamide (47, [M + H]^+^ at *m/z* 256.2644), eicosenamide (53, [M + H]+ at *m/z* 310.3102), and erucamide (54, [M + H]^+^ at *m/z* 338.3418) 75. Sphingolipids included C17-sphinganine (41, [M + H]^+^ at *m/z* 288.2891) and diacetylsphingosine (50, 52, [M+Na]+ at m/z 408.3085) (Supplementary Fig. S27, S28). Dehydro-*α*-tocopherol (55, [M + H]^+^ at *m/z* 429.3721) was identified in inoculated fresh oils (Supplementary Fig. S29).

UPLC–HRMS/MS coupled with FBMN substantially expanded the detectable chemical space of edible oils and their gut microbiota-transformed products beyond GC–MS alone, particularly for peptides, cyclopeptides, indole alkaloids, sphingolipids, and tocopherol derivatives.

### Multivariate data analyses of UPLC–HRMS/MS dataset

Multivariate data analysis was performed on the UPLC–HRMS/MS dataset to evaluate the impact of oxidation and gut microbiota interaction on the secondary metabolite composition of edible oils and to facilitate marker discovery. Both unsupervised and supervised models were constructed separately for each oil type using biological triplicates for each experimental condition.

For corn oil, unsupervised HCA and PCA analyses successfully discriminated inoculated samples from their corresponding uninoculated counterparts (Supplementary Fig. S30A). The PCA score plot showed clear separation of uninoculated fresh and oxidized samples (COFU and COOU) from inoculated fresh and oxidized samples (COFI and COOI) along PC1, which explained 40% of the variance, while the first two components together accounted for 54% of the total variance (Supplementary Fig. S30B). Inspection of the loading plot indicated that cyclo-(Pro–Leu) and 3-[(1-methyl-1H-indol-3-yl)methyl]-2,3-dihydro-1H-indol-2-one were enriched in inoculated samples, whereas 1,2-diacetylsphingosine was more abundant in uninoculated samples (Supplementary Fig. S30C).

Similarly, PCA modelling of sunflower oil samples (Supplementary Fig. S31A) revealed a clear separation between uninoculated samples (SFFU and SFOU) and most inoculated samples (SFFI and SFOI), with PC1 explaining 48% of the variance and the first two components together explaining 59% of the total variance (Supplementary Fig. S31B). The corresponding loading plot showed that N(3)-fumaramoyl-2,3-diaminopropanoic acid was enriched in inoculated samples, whereas cyclo-(Pro–Leu) and 1,2-diacetylsphingosine were more abundant in uninoculated samples (Supplementary Fig. S31C).

**Fig. 3:**
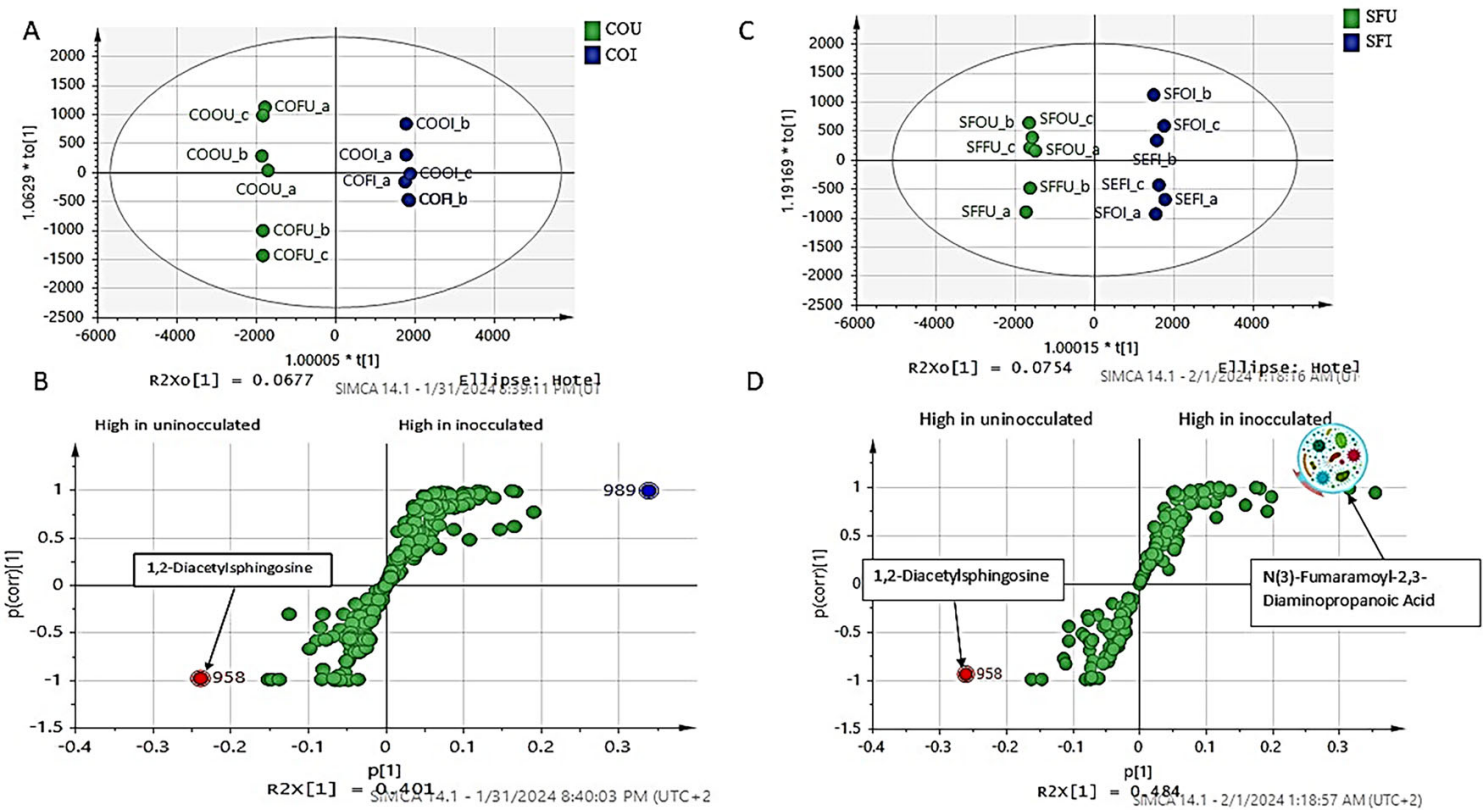
UPLC–HRMS/MS based OPLS-DA modelling of corn and sunflower oils samples. **A** OPLS-DA score of corn oil, **B** S-loading plot of OPLS model of corn oil, **C** OPLS-DA score of sunflower oil, **D** S-loading Plot of OPLS model of sunflower oil.

To confirm the PCA-derived patterns and to identify discriminant markers more robustly, supervised OPLS-DA models were constructed. In corn oil, OPLS-DA clearly separated uninoculated samples (COFU and COOU) from inoculated samples (COFI and COOI) (Fig. 3A). The corresponding S-loading plot revealed 1,2-diacetylsphingosine as a marker of uninoculated samples, whereas *N*(3)-fumaramoyl-2,3-diaminopropanoic acid was strongly associated with inoculated samples (Fig. 3B).

Similarly, in sunflower oil, OPLS-DA achieved clear discrimination between uninoculated samples (SFFU and SFOU) and inoculated samples (SFFI and most SFOI replicates) (Fig. 3C). *N*(3)-fumaramoyl-2,3-diaminopropanoic acid emerged as a key marker associated with gut microbiota-treated samples (Fig. 3D).

Multivariate clustering indicated that gut microbiota incubation constituted the major source of variance across samples, while thermal oxidation effects were evident at the metabolite level. Similar patterns have been reported in ex vivo gut microbiota metabolomics studies^33,34^.

## Discussion

The present study provides a comprehensive system-level evaluation of how thermal oxidation and gut microbiota fermentation jointly reshape the chemical landscape of edible oils. By integrating GC-MS and UPLC-HRMS/MS metabolomics combined with FBMN and multivariate statistical analysis, the work demonstrates that edible oils behave as chemically dynamic matrices undergoing sequential transformations driven initially by thermal oxidation and subsequently by microbial metabolism. The complementary analytical platforms enabled simultaneous characterization of volatile primary metabolites, structurally complex secondary metabolites, and nitrogen-containing compounds that cannot be captured using a single analytical workflow, thereby offering a holistic understanding of oil compositional evolution under gastrointestinal-like conditions.

Peroxide value measurements confirmed successful induction of lipid oxidation, validating the experimental design and ensuring that subsequent metabolomic differences reflected controlled oxidative processes rather than analytical variability. The increased PV values observed in oxidized corn, sesame, and sunflower oils agree with established lipid autoxidation mechanisms involving hydroperoxide formation as primary oxidation products. These chemical changes provided the foundation for interpreting downstream metabolomic alterations detected by GC-MS and UPLC-HRMS/MS analyses.

GC–MS profiling revealed that oxidation primarily influenced lipid-derived metabolites, particularly fatty acids, aldehydes, tocopherols, and low-molecular-weight organic acids. Increased relative levels of free oleic and linoleic acids in oxidized oils are consistent with partial hydrolysis and oxidative cleavage of triacylglycerols during heating rather than an absolute increase in fatty acid content^5^. Concurrent formation of short- and medium-chain acids such as caproic, octanoic, and 4-methylvaleric acids supports oxidative fragmentation of unsaturated fatty acids, processes known to contribute to rancidity development^22,35^. In contrast, depletion of several organic acids likely reflects volatilization or further degradation during prolonged thermal exposure.

Aldehydes emerged as prominent markers of oxidation, with notable increases in 2,4-decadienal and 2,4-nonadienal, well-established degradation products of linoleic acid oxidation^14,21^. Their accumulation confirms progression toward secondary oxidation stages and aligns with earlier studies on heated vegetable oils^23,24^. The toxicological relevance of these compounds is noteworthy, as 2,4-decadienal has been associated with cytotoxic and mutagenic effects^25,26^. Simultaneously, tocopherol depletion observed after oxidation reflects their consumption as radical scavengers during lipid peroxidation^16,36^, further confirming oxidative stress progression.

Beyond oxidation alone, ex vivo incubation with human gut microbiota induced profound remodeling of oil metabolomes. GC-MS analysis revealed the emergence of approximately 25 metabolites exclusively after microbial incubation, including amino acids, nucleobases, indole derivatives, and phenolic compounds, demonstrating extensive microbial biotransformation. The pronounced reduction of organic acids, fatty acids, aldehydes, and several processing-related contaminants indicates active microbial utilization and metabolic conversion. For example, depletion of oxalic acid agrees with microbial oxalate metabolism reported for *Oxalobacter* and *Lactobacillus* species^37^, while reductions in glycidyl fatty acid esters suggest microbial degradation of potentially harmful contaminants capable of releasing glycidol in the gastrointestinal tract^38^.

Nitrogen-containing metabolites exhibited the most prominent microbiota-driven changes. Accumulation of indole and related metabolites, including indole-3-acetic acid and indole-3-propionic acid, reflects active microbial tryptophan metabolism consistent with known activities of gut bacterial genera such as *Escherichia*, *Clostridium*, *Proteus*, and *Bacteroides*^39^. Likewise, strong enrichment of phenol indicates microbial fermentation of aromatic amino acids under anaerobic gut conditions^40^. These observations highlight the dualistic nature of microbial metabolism, involving simultaneous depletion of certain harmful compounds alongside formation of new bioactive or potentially harmful metabolites.

Multivariate analyses of the GC–MS dataset reinforced these findings by demonstrating that microbiota incubation constituted the dominant source of variance across samples. PCA and HCA consistently separated inoculated from uninoculated oils (Fig. 1 and Supplementary Figs. S4, S6), while OPLS-DA models identified discriminant metabolites such as indole, phenol, propylene glycol, and 4-methylvaleric acid as key contributors to microbial signatures. The inability to construct a valid oxidation-only OPLS-DA model for sunflower oil further indicates that microbial metabolism exerted a stronger compositional influence than oxidation under the applied experimental conditions.

UPLC–HRMS/MS analysis substantially expanded metabolome coverage toward medium- and high-polarity secondary metabolites. FBMN visualization (Fig. 2) revealed complex molecular relationships among peptides, cyclopeptides, indole alkaloids, fatty acid amides, and sphingolipids. Peptides represented the largest annotated chemical family, detected predominantly in sunflower oil, suggesting matrix-dependent release or formation of peptide-like constituents. Characteristic MS/MS fragmentation patterns and immonium ions supported confident peptide annotation consistent with established peptide fragmentation behavior^30^.

Cyclopeptides, including diketopiperazines such as cyclo-(Pro–Leu) and cyclo-(Leu–Leu), expand the known chemical composition of edible oils beyond traditional lipid constituents. Their occurrence supports growing evidence that lipid-rich foods contain overlooked nitrogenous metabolites detectable only through high-resolution LC-MS approaches^41^. Detection of *β*-carboline and other indole alkaloids exclusively after microbial incubation further confirms active microbiota-mediated tryptophan transformation pathways^42–44^.

Sphingolipids identified for the first time in these oils, including C17-sphinganine and diacetylsphingosine, represent another important discovery. Multivariate analysis of the LC–MS dataset demonstrated clear separation between inoculated and uninoculated samples for both corn and sunflower oils (Supplementary Figs. S30 and S31), confirming that microbial activity dominates secondary metabolite variation. In sunflower oil, enrichment of *N*(3)-fumaramoyl-2,3-diaminopropanoic acid in inoculated samples contrasted with higher levels of cyclo-(Pro–Leu) and diacetylsphingosine in uninoculated oils (Supplementary Fig. S31C). Proposed microbial transformation pathways illustrated in Supplementary Fig. S32 suggest hydrolysis of cyclic peptides and stepwise deacetylation of sphingolipids, indicating active remodeling of peptide-like and sphingolipid-related constituents during gut microbiota incubation.

Supervised OPLS-DA models confirmed these patterns (Fig. 3A–D), identifying 1,2-diacetylsphingosine as a marker of uninoculated samples and *N*(3)-fumaramoyl-2,3-diaminopropanoic acid as a microbiota-associated metabolite. Collectively, these results demonstrate that oxidation establishes the initial chemical substrate pool, whereas gut microbiota metabolism acts as the principal driver reshaping metabolite composition, consistent with previous ex vivo gut metabolomics investigations^33,34^.

**Fig. 4:**
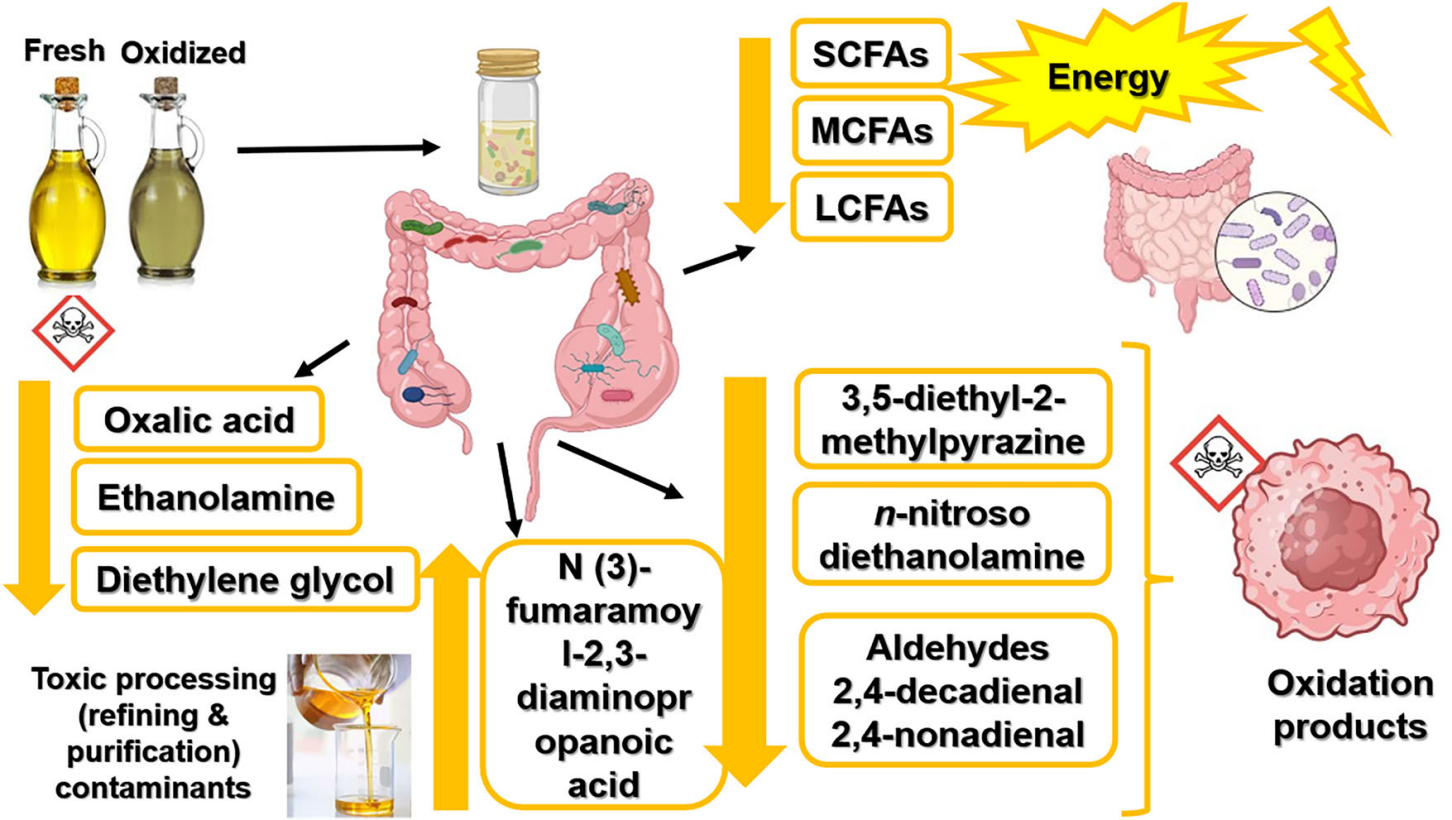
Summary of gut microbiota interaction with fresh and oxidized oils.

The overall putative oxidative and microbial transformation pathways, detoxification routes, and formation of new bioactive or potentially harmful metabolites are summarized schematically in Fig. 4. Thermal oxidation generates reactive lipid-derived intermediates and degradation products, some representing potentially hazardous compounds. Subsequent microbial metabolism transforms, consumes, or converts many of these molecules while simultaneously producing new metabolites derived from amino acid fermentation and nitrogen metabolism. Thus, edible oils entering the gastrointestinal tract should be considered chemically evolving substrates rather than static dietary lipids.

Several limitations should be considered when interpreting these findings. The ex vivo fermentation model cannot fully reproduce host physiological conditions, intestinal absorption, or immune interactions, and the use of microbiota derived from a single donor limits extrapolation to broader population variability. Consequently, the results reflect metabolic potential rather than universal human responses. In addition, metabolite identification relied primarily on annotation-level confidence without absolute quantification or full structural confirmation for all compounds, which will be necessary to establish biological relevance.

Despite these limitations, the study demonstrates that combining complementary analytical platforms with network-based metabolomics provides an unprecedented view of edible oil chemistry during oxidation and gut microbial fermentation. The findings reveal simultaneous depletion of several oxidation products and processing contaminants alongside formation of new microbial metabolites, highlighting the complex and bidirectional nature of oil–microbiota interactions. Future research should extend toward multi-donor microbiota models, targeted quantitative validation, microbial strain attribution, and in vivo investigations to determine the physiological consequences of the identified transformations. The integrative workflow established here provides a robust framework for investigating food quality, safety, and host–microbiota chemical interactions and advances understanding of how dietary lipids are chemically reshaped within the gut environment.

## Methods

### Chemicals and oil samples

Fresh corn, sesame, and sunflower oils were obtained from the Oil Center, National Research Centre (Cairo, Egypt). Oxidized oil samples were prepared in-house by accelerated thermal oxidation as described below. Sample codes are listed in Table 2. All solvents and chemicals were of analytical grade and purchased from Sigma-Aldrich (St. Louis, MO, USA). All experiments were performed in triplicate (*n* = 3) to account for analytical and biological variability.

**Table 2:** Sample codes of oil samples used in this study

Sample Code	Oil type	Treatment
COOU	Corn oil	Oxidized, uninoculated
COOI	Corn oil	Oxidized, inoculated
COFU	Corn oil	Fresh, uninoculated
COFI	Corn oil	Fresh, inoculated
SOOU	Sesame oil	Oxidized, uninoculated
SOOI	Sesame oil	Oxidized, inoculated
SOFU	Sesame oil	Fresh, uninoculated
SOFI	Sesame oil	Fresh, inoculated
SFOU	Sunflower oil	Oxidized, uninoculated
SFOI	Sunflower oil	Oxidized, inoculated
SFFU	Sunflower oil	Fresh, uninoculated
SFFI	Sunflower oil	Fresh, inoculated
COO	Corn oil	Oxidized
COF	Corn oil	Fresh
SOO	Sesame oil	Oxidized
SOF	Sesame oil	Fresh
SFO	Sunflower oil	Oxidized
SFF	Sunflower oil	Fresh

### Oils’ rancidity determination *via* peroxide value PV

Three independent sets were prepared for corn, sesame, and sunflower oils. Accelerated oxidation of edible oils was induced by storing samples in the dark at 60 °C to simulate extended oxidative changes under controlled conditions, and peroxide value was monitored periodically as an indicator of primary oxidation. Accelerated storage or Schaal oven tests at moderately elevated temperatures (60–65 °C) are commonly used to assess oxidative stability and track PV changes over time^45,46^. All measurements were performed in triplicate and expressed as mean ± standard deviation (SD).

PV was determined according to ref. ^19^. Briefly, oil samples (3 g) were dissolved in a mixture of glacial acetic acid (30 mL) and chloroform (20 mL) (3:2, *v/v*), followed by the addition of saturated potassium iodide solution (1 mL). The mixture was shaken for 30 s and kept in the dark for 1 min. Distilled water (50 mL) was then added, and the liberated iodine was titrated against 0.01 N sodium thiosulfate. Peroxide value (meq/kg) was calculated using Eq. 1:

1 $${\rm{PV}}=1000\times ({\rm{S}}\times {\rm{N}})/{\rm{W}}$$

where S is the volume of sodium thiosulfate solution (blank-corrected, mL), N is the normality of sodium thiosulfate, and W is the sample weight (g)

### Ex vivo culture of a human gut microbiome sample and incubation with oil samples

Ex vivo cultivation of the human gut microbiota was performed according to the protocol described by ref. ^47^ using a single healthy adult donor fecal sample (pilot donor). Fecal microbiota used for ex vivo fermentation experiments were obtained from a healthy adult female donor recruited as part of a previously established human microbiome cohort^47^. The donor was ≥18 years old, in good health at the time of sampling, with no history of diabetes, gastrointestinal, oral, or skin infections, malignancies, or other chronic diseases, and no antibiotic use within three months prior to sample collection. Samples were provided in anonymized form; therefore, individual metadata such as BMI and dietary background were not available. These eligibility criteria ensured the suitability of the microbiota for controlled ex vivo metabolism studies. The fecal material had been previously processed and stored as a glycerol stock at −80 °C. All cultivation and handling steps were carried out under strictly anaerobic conditions to preserve microbial viability and community structure.

For culture initiation, approximately 100 µL of the donor glycerol stock was inoculated into 2 mL of modified Gifu anaerobic medium (mGAM) broth and incubated anaerobically at 37 °C. From this primary culture, 20 µL aliquots were transferred into individual culture tubes containing fresh mGAM medium and pre-incubated for 24 h at 37 °C to allow microbial adaptation and stabilization prior to treatment.

Fresh and thermally oxidized corn, sesame, and sunflower oils were prepared as 200 mg/mL stock suspensions in DMSO and sonicated for 10 min to ensure homogeneity. These stocks were added to pre-incubated microbial cultures to achieve a final concentration of 1 mg/mL, with parallel DMSO-only controls. This concentration was chosen based on ex vivo system performance: higher levels impaired microbial growth due to phase separation and toxicity, while lower levels yielded insufficient metabolite detection. The selected dose balances microbial viability and robust metabolomic profiling, following precedents in gut microbiota batch-culture studies optimized for functional assessment rather than direct dietary replication^33,34,47^.

Fecal fermentation experiments were performed using anaerobic batch ex vivo cultures incubated at 37 °C for 24 h. Samples were collected at 0 h (immediately after oil addition) and after 24 h incubation for metabolomic analysis from three independent cultures representing biological replicates. The 24 h incubation period was selected based on established ex vivo gut microbiota metabolomics workflows demonstrating that this duration allows sufficient microbial growth and substrate conversion while maintaining community stability and metabolic activity. Similar endpoint-based experimental designs have been widely employed to investigate microbial metabolism of dietary constituents and xenobiotics using human fecal consortia^33,34,47,48^.

All incubations were performed anaerobically at 37 °C for the specified incubation period prior to metabolite extraction and chemical analysis. Each treatment group consisted of three independent biological replicates (*n* = 3) prepared from separate fermentation cultures initiated under identical experimental conditions. These replicates represent biological variability arising from ex vivo microbiota incubation rather than repeated analytical injections. Such triplicate culture designs are commonly employed in gut microbiota batch fermentation metabolomics studies to ensure experimental reproducibility while minimizing donor-related variability^17,34,47^.

### Extraction, GC–MS analysis, and identification of primary metabolites

Primary metabolite analysis was performed following previously established GC–MS metabolomics workflows^17,49^ with minor modifications. In brief, 100 µL of each fresh and oxidized oil sample was extracted with 1 mL of 100% methanol, sonicated for 30 min, and centrifuged at 12,000 rpm for 10 min to remove particulates. An aliquot (100 µL) of the supernatant was evaporated to dryness under a nitrogen stream. For derivatization, the dried residue was treated with 150 µL of *N*-methyl-*N*-(trimethylsilyl) trifluoroacetamide (MSTFA) and incubated at 60 °C for 45 min prior to GC–MS analysis, following standard silylation-based metabolomics protocols.

For gut microbiota–incubated samples, 2 mL of culture medium was extracted with 4 mL of ethyl acetate with vigorous shaking. The organic phase was separated, and a 100 µL aliquot was evaporated to dryness using a speed vacuum concentrator, derivatized with 150 µL MSTFA as described above, and subjected to GC–MS analysis.

GC–MS analysis was performed using a Shimadzu GC-17A gas chromatograph coupled to a Shimadzu QP5050A mass spectrometer equipped with an Rtx-5MS column (30 m × 0.25 mm i.d., 0.25 µm film thickness). Samples were injected in split mode (1:15) under the following conditions: injector temperature 280 °C; column oven initially set at 80 °C for 2 min, then increased at 5 °C/min to 315 °C and held for 12 min; helium carrier gas at 1 mL/min. The transfer line and ion-source temperatures were set at 280 °C and 180 °C, respectively. The mass spectrometer operated under electron ionization (EI) at 70 eV with a scan range of 50–650 *m/z*. These conditions follow previously validated GC–MS metabolomics protocols applied for profiling primary metabolites in complex food and plant matrices^17,49^.

Chromatographic peaks were deconvoluted using AMDIS software (www.amdis.net). Metabolite identification was achieved through a combined strategy including spectral matching against NIST and WILEY mass spectral libraries, calculation of retention indices (RI) relative to a homologous *n*-alkane series (C8–C40), and comparison with literature data and authentic reference standards when available^17,49,50^.

### Extraction and analysis using UPLC–HRMS/MS

Gut culture samples from the different treatment groups were extracted with ethyl acetate. Briefly, 2 mL of each culture was spiked with umbelliferone as an internal standard (final concentration 10 µg/mL) and extracted with 4 mL of 100% ethyl acetate with vigorous shaking. The organic phase was separated, and aliquots of 3 mL were evaporated to dryness using a speed vacuum concentrator. The residue for UPLC–HRMS/MS analysis was reconstituted in 300 µL methanol and subjected to LC–MS analysis.

UPLC–HRMS/MS analysis was performed using an Agilent quadrupole time-of-flight (QTOF) mass spectrometer coupled to a Poroshell 120 EC-C18 column (2.7 µm, 2.1 × 100 mm). The mobile phase consisted of solvent A (0.1% formic acid in water) and solvent B (0.1% formic acid in acetonitrile). The flow rate was set to 0.25 mL/min. The gradient elution program was as follows: 0.5% B for 1 min, followed by a linear increase to 100% B over 20 min and a 5 min hold at 100% B.

Mass spectrometric detection was performed in positive electrospray ionization (ESI+) mode under the following conditions: capillary voltage 3.5 kV, nebulizer gas pressure 35 psi, drying gas temperature 325 °C, and drying gas flow rate 10 L/min. Data were acquired over a mass range of m/z 100–1700 in full-scan mode. Internal mass calibration was applied using reference ions to ensure mass accuracy below 5 ppm.

Tandem mass spectra (MS/MS) were acquired using data-dependent acquisition (DDA), in which the five most intense precursor ions per scan cycle were automatically selected for fragmentation. Collision-induced dissociation (CID) was performed using stepped collision energies of 10, 20, and 40 eV to generate diagnostic fragmentation patterns for metabolite annotation. Dynamic exclusion (0.3 min) was enabled to minimize repeated fragmentation of the same ions. Raw data files were subsequently converted and processed prior to FBMN analysis following established metabolomics workflows for QTOF-based untargeted profiling^49,51^.

### Feature-based molecular networking and compound dereplication

Raw UPLC–HRMS/MS data were processed using MZmine 2 to generate MS/MS-resolved feature lists compatible with the Global Natural Product Social Molecular Networking (GNPS) -FBMN workflow, following established metabolomics and GNPS guidelines^52,53^. Feature detection was conducted using centroid mass detection with a noise level of 1.0E3, followed by chromatogram building with a minimum time span of 0.1 min and minimum peak height of 1.0E4. Chromatographic deconvolution was performed using the local minimum search algorithm, and peak alignment across samples employed a mass tolerance of 0.01 Da and retention time tolerance of 0.2 min. After blank subtraction and data filtering, a total of 803 features were retained and exported as MGF files together with feature quantification tables for GNPS analysis. MS/MS spectra were further filtered by removing fragment ions within ±17 Da of the precursor *m/z* and retaining only the six most intense fragment ions within a ± 50 Da window across the spectrum. Molecular networks were generated using a cosine similarity score threshold of 0.7 with a minimum of four matched fragment ions. Network visualization and exploration were performed using Cytoscape version 3.10.2, while putative metabolite annotation was supported using SIRIUS + CSI:FingerID (version 5.8.6) based on molecular formula prediction and structure candidate searches against PubChem with a mass error tolerance of 10 ppm^53^. The positive-mode FBMN job is publicly accessible at: https://gnps.ucsd.edu/ProteoSAFe/status.jsp?task=f5af098ce93143c98ff0c9f3d55e2f53.

### Multivariate data analyses of GC-MS and UPLC-MS/MS datasets

For chemometric analysis, peak intensity matrices were generated separately for GC–MS and UPLC–HRMS/MS datasets to ensure optimal preprocessing according to platform-specific requirements. GC–MS raw data were processed using MS-DIAL software, which enabled peak detection, spectral deconvolution, retention time alignment, and gap filling using default parameters, yielding quantitative feature tables suitable for metabolomics analysis as previously described^54^.

In contrast, UPLC–HRMS/MS data were processed using MZmine 2, allowing feature detection, chromatographic alignment, and construction of MS/MS-resolved feature tables compatible with downstream molecular networking workflows^55^. The resulting peak intensity matrices were exported for further statistical analysis.

The processed datasets were subjected to unsupervised HCA and PCA, followed by OPLS-DA using SIMCA-P version 14.1 (Umetrics, Umeå, Sweden). Prior to modeling, variables were mean-centered and Pareto-scaled. PCA was initially applied to visualize overall clustering patterns and intrinsic sample variability, whereas OPLS-DA was employed to enhance class discrimination and identify metabolites contributing to group separation.

Model performance and robustness were evaluated using cumulative R^2^ (goodness of fit) and Q^2^ (predictive ability) values, with validation performed through 200 permutation tests. Potential outliers were assessed using DModX (distance to the model) and Hotelling’s T^2^ statistics. Although the number of biological replicates per group was limited (*n* = 3), model reliability was supported by combined unsupervised PCA inspection and supervised OPLS-DA validation, an approach commonly applied in metabolomics studies of gut microbiota batch cultures with comparable experimental designs^33,34^.

### Declaration of generative AI and AI-assisted technologies in the writing process

During the preparation of this work, ChatGPT (OpenAI) was used in order to improve the readability and language of the manuscript. After using this tool/service, the authors reviewed and edited the content as needed and took full responsibility for the content of the published article.

## Supplementary information

Supplementary Materials

## Data Availability

No data were generated or analysed during the current study.
