# Supplementary material for: Gut microbiota-driven remodeling of fresh and oxidized edible oils revealed by integrated GC-MS and UPLC-HRMS/MS metabolomics
Source: NPJ Sci Food. 2026 May 5;10:144. doi: 10.1038/s41538-026-00861-0 (PMC13144732; doi:10.1038/s41538-026-00861-0)
Supplement: Supplementary file 1 — Supplementary Materials [file 41538_2026_861_MOESM1_ESM.docx]

**Supplementary Materials**

# **List of Supplementary Tables**

**Suppl. Table S1:** The peroxide value of (Corn, sesame, and Sunflower) oil samples.

| Sample code | Oil type | Treatment | Peroxide value |
| --- | --- | --- | --- |
| COF | Corn oil | Fresh | 2 meq/Kg |
| COO | Corn oil | Oxidized | 39 meq/Kg |
| SOF | Sesame oil | Fresh | 4 meq/Kg |
| SOO | Sesame oil | Oxidized | 43 meq/Kg |
| SFF | Sunflower oil | Fresh | 3 meq/Kg |
| SFO | Sunflower oil | Oxidized | 52 meq/Kg |

**Suppl. Table S2:**  Levels of silylated primary metabolites in COOU, COOI, COFU, COFI, SOOU, SOOI, SOFU, SOFI, SFOU, SFOI, SFFU, and SFFI. Oils were analyzed *via* GC-MS. Results are expressed as a relative percentile of the total peak areas and n=3. For codes explanation, refer to **Table 1**.

| Peak no. | Average  Rt (min) | Name | Class | SOOU | SOOI | SOFU | SOFI | SFOU | SFOI | SFFU | SFFI | COOU | COOI | COFU | COFI |
| --- | --- | --- | --- | --- | --- | --- | --- | --- | --- | --- | --- | --- | --- | --- | --- |
| 2 | 4.749 | 4-Methylvaleric acid | Acids | 0.50  ± 0.26 | 11.76  ± 0.11 | 1.27  ± 0.44 | 12.26  ± 0.82 | 1.16  ± 0.32 | 14.07  ± 2.82 | 1.01  ± 0.47 | 14.39  ± 3.20 | 0.90  ± 0.0002 | 12.90  ± 0.32 | 1.38  ± 0.13 | 13.74  ± 0.55 |
| 6 | 5.128 | Lactic acid |  | 8.57  ± 2.24 | 0.60  ± 0.19 | 10.63  ± 1.98 | 0.39  ± 0.11 | 7.01  ± 1.34 | 0.47  ± 0.16 | 7.15  ± 1.27 | 0.42  ± 0.18 | 6.10  ± 0.33 | 0.31  ± 0.04 | 9.45  ± 2.70 | 0.39  ± 0.23 |
| 7 | 5.143 | *α*-Ketovaleric acid |  | 1.28  ± 0.09 | 0.32  ± 0.003 | 1.35  ± 0.07 | 0.23  ± 0.08 | 1.30  ± 0.24 | 0.15  ± 0.01 | 1.31  ± 0.09 | 0.22  ± 0.07 | 1.26  ± 0.15 | 0.19  ± 0.01 | 1.38  ± 0.22 | 0.17  ± 0.04 |
| 8 | 5.248 | Caproic acid |  | 0.16  ± 0.03 | 0.13  ± 0.004 | 0.27  ± 0.05 | 0.12  ± 0.02 | 0.25  ± 0.04 | 0.09  ± 0.03 | 0.31  ± 0.04 | 0.12  ± 0.02 | 0.24  ± 0.02 | 0.13  ± 0.003 | 0.29  ± 0.01 | 0.11  ± 0.01 |
| 9 | 5.335 | Glycolic acid |  | 1.02  ± 0.005 | 0.33  ± 0.01 | 0.98  ± 0.01 | 0.27  ± 0.06 | 1.16  ± 0.20 | 0.25  ± 0.12 | 1.11  ± 0.13 | 0.27  ± 0.07 | 1.09  ± 0.17 | 0.23  ± 0.01 | 1.05  ± 0.09 | 0.20  ± 0.04 |
| 10 | 5.477 | 2-Ketoisocaproic acid |  | 0.05  ± 0.07 | 0.57  ± 0.003 | 0.15  ± 0.08 | 0.48  ± 0.16 | 0.09  ± 0.12 | 0.54  ± 0.09 | 0.36  ± 0.27 | 0.49  ± 0.16 | 0.07  ± 0.04 | 0.58  ± 0.05 | 0.12  ± 0.08 | 0.42  ± 0.19 |
| 11 | 5.523 | Pyruvic acid, enol |  | 0.62  ± 0.81 | 0.12  ± 0.02 | 0.14  ± 0.10 | 0.11  ± 0.05 | 0.08  ± 0.11 | 0.11  ± 0.04 | 0.68  ± 0.19 | 0.10  ± 0.04 | 0.17  ± 0.07 | 0.13  ± 0.02 | 0.13  ± 0.06 | 0.10  ± 0.04 |
| 12 | 5.644 | 2-Ethyl hexanoic acid |  | 1.35  ± 0.13 | 0.43  ± 0.02 | 1.38  ± 0.10 | 0.31  ± 0.10 | 1.45  ± 0.31 | 0.43  ± 0.41 | 1.54  ± 0.17 | 0.30  ± 0.11 | 1.39  ± 0.17 | 0.26  ± 0.01 | 1.37  ± 0.23 | 0.23  ± 0.06 |
| 13 | 5.757 | Oxalic acid |  | 5.41  ± 0.34 | 1.86  ± 0.04 | 5.99  ± 0.74 | 1.47  ± 0.30 | 6.25  ± 1.00 | 1.20  ± 0.39 | 6.80  ± 1.69 | 1.40  ± 0.36 | 6.16  ± 1.30 | 1.22  ± 0.08 | 6.30  ± 0.89 | 1.27  ± 0.53 |
| 14 | 6.164 | Heptanoic acid |  | 0.08  ± 0.04 | 0.03  ± 0.004 | 0.06  ± 0.03 | 0.02  ± 0.004 | 0.06  ± 0.03 | 0.09  ± 0.10 | 0.08  ± 0.02 | 0.03  ± 0.02 | 0.12  ± 0.002 | 0.02  ± 0.02 | 0.13  ± 0.09 | 0.06  ± 0.06 |
| 16 | 6.349 | Beta-Lactic acid |  | 0.17  ± 0.03 | 0.01  ± 0.001 | 0.19  ± 0.07 | 0.04  ± 0.01 | 0.16  ± 0.03 | 0.02  ± 0.02 | 0.08  ± 0.03 | 0.04  ± 0.002 | 0.13  ± 0.03 | 0.04  ± 0.004 | 0.15  ± 0.03 | 0.02  ± 0.01 |
| 17 | 6.364 | Beta-Lactic acid |  | 0.50  ± 0.10 | 0.17  ± 0.004 | 0.57  ± 0.04 | 0.13  ± 0.02 | 0.57  ± 0.03 | 0.18  ± 0.15 | 0.56  ± 0.08 | 0.14  ± 0.04 | 0.51  ± 0.09 | 0.11  ± 0.002 | 0.62  ± 0.05 | 0.10  ± 0.03 |
| 18 | 6.45 | Beta-amino isobutyric acid |  | 0.17  ± 0.26 | 0.07  ± 0.002 | 0.42  ± 0.07 | 0.09  ± 0.06 | 0.17  ± 0.25 | 0.05  ± 0.04 | 0.00  ± 0.00 | 0.07  ± 0.02 | 0.05  ± 0.01 | 0.07  ± 0.01 | 0.45  ± 0.11 | 0.04  ± 0.03 |
| 21 | 7.074 | 3-Hydroxyisovaleric acid |  | 0.03  ± 0.03 | 0.39  ± 0.001 | 0.04  ± 0.01 | 0.05  ± 0.05 | 0.21  ± 0.34 | 0.07  ± 0.05 | 0.04  ± 0.02 | 0.09  ± 0.07 | 0.03  ± 0.02 | 0.13  ± 0.06 | 0.26  ± 0.36 | 0.12  ± 0.01 |
| 25 | 7.744 | 2-Hydroxyisocaproic acid |  | 0.03  ± 0.001 | 0.02  ± 0.0004 | 0.02  ± 0.005 | 0.04  ± 0.01 | 0.03  ± 0.02 | 0.02  ± 0.02 | 0.01  ± 0.01 | 0.04  ± 0.01 | 0.02  ± 0.02 | 0.03  ± 0.003 | 0.03  ± 0.02 | 0.03  ± 0.004 |
| 26 | 7.812 | 2-Hydroxy-3-methylvaleric acid |  | 0.07  ± 0.11 | 0.04  ± 0.001 | 0.22  ± 0.03 | 0.04  ± 0.02 | 0.09  ± 0.16 | 0.03  ± 0.03 | 0.00  ± 0.00 | 0.04  ± 0.01 | 0.01  ± 0.003 | 0.04  ± 0.01 | 0.26  ± 0.09 | 0.03  ± 0.02 |
| 28 | 7.859 | Benzoic acid |  | 0.01  ± 0.01 | 0.004  ± 0.002 | 0.01  ± 0.01 | 0.01  ± 0.01 | 0.01  ± 0.01 | 0.004  ± 0.005 | 0.02  ± 0.01 | 0.01  ± 0.002 | 2.69  ± 2.19 | 0.01  ± 0.004 | 0.05  ± 0.03 | 0.01  ± 0.01 |
| 29 | 8.057 | Octanoic acid |  | 1.23  ± 0.06 | 0.35  ± 0.005 | 1.19  ± 0.29 | 0.25  ± 0.05 | 1.29  ± 0.16 | 0.55  ± 0.59 | 1.24  ± 0.24 | 0.31  ± 0.12 | 1.32  ± 0.17 | 0.23  ± 0.02 | 1.30  ± 0.11 | 0.22  ± 0.05 |
| 33 | 8.362 | Phosphoric acid |  | 0.31  ± 0.09 | 0.06  ± 0.003 | 0.21  ± 0.01 | 0.05  ± 0.02 | 0.28  ± 0.09 | 0.03  ± 0.01 | 0.30  ± 0.03 | 0.05  ± 0.02 | 0.17  ± 0.02 | 0.04  ± 0.004 | 0.26  ± 0.01 | 0.04  ± 0.01 |
| 37 | 8.573 | Phenylacetic acid |  | 1.97  ± 0.44 | 1.20  ± 0.02 | 1.48  ± 0.17 | 1.79  ± 0.59 | 1.90  ± 0.47 | 1.51  ± 0.20 | 2.39  ± 0.36 | 1.88  ± 0.69 | 1.67  ± 0.69 | 1.48  ± 0.18 | 1.63  ± 0.13 | 1.42  ± 0.13 |
| 39 | 8.806 | Succinic acid |  | 2.31  ± 0.04 | 0.85  ± 0.01 | 2.24  ± 0.16 | 0.57  ± 0.16 | 2.53  ± 0.45 | 0.48  ± 0.16 | 2.78  ± 0.38 | 0.53  ± 0.18 | 2.63  ± 0.35 | 0.48  ± 0.02 | 2.48  ± 0.38 | 0.46  ± 0.10 |
| 43 | 9.13 | Glyceric acid |  | 2.70  ± 0.50 | 0.86  ± 0.07 | 2.45  ± 0.05 | 0.59  ± 0.17 | 2.65  ± 0.46 | 0.56  ± 0.28 | 3.34  ± 0.67 | 0.56  ± 0.16 | 2.85  ± 0.53 | 0.49  ± 0.02 | 2.51  ± 0.31 | 0.47  ± 0.07 |
| 45 | 9.388 | Nonanoic acid |  | 14.29  ± 1.31 | 4.68  ± 0.001 | 13.59  ± 0.29 | 3.39  ± 1.12 | 15.60  ± 3.24 | 2.48  ± 0.38 | 16.27  ± 2.02 | 3.28  ± 0.97 | 14.77  ± 1.59 | 2.77  ± 0.13 | 14.30  ± 2.34 | 2.56  ± 0.65 |
| 48 | 10.038 | Glutaric acid |  | 0.05  ± 0.06 | 0.001  ± 0.001 | 0.002  ± 0.004 | 0.002  ± 0.0004 | 0.01  ± 0.003 | 0.001  ± 0.002 | 0.01  ± 0.001 | 0.001  ± 0.0002 | 0.20  ± 0.18 | 0.002  ± 0.002 | 0.004  ± 0.004 | 0.002  ± 0.00 |
| 50 | 10.183 | Hydrocinnamic acid |  | 0.09  ± 0.07 | 0.19  ± 0.004 | 0.07  ± 0.02 | 0.31  ± 0.06 | 0.09  ± 0.03 | 0.23  ± 0.03 | 0.06  ± 0.02 | 0.30  ± 0.06 | 0.07  ± 0.004 | 0.29  ± 0.01 | 0.10  ± 0.04 | 0.25  ± 0.02 |
| 52 | 10.67 | Decanoic acid |  | 0.23  ± 0.10 | 0.03  ± 0.01 | 0.25  ± 0.14 | 0.02  ± 0.01 | 0.10  ± 0.04 | 0.02  ± 0.02 | 0.28  ± 0.19 | 0.02  ± 0.001 | 0.03  ± 0.03 | 0.02  ± 0.01 | 0.22  ± 0.18 | 0.01  ± 0.01 |
| 61 | 12.985 | 3-Hydroxyphenylacetic acid |  | 0.48  ± 0.10 | 0.08  ± 0.002 | 0.34  ± 0.03 | 0.06  ± 0.01 | 0.34  ± 0.03 | 0.18  ± 0.22 | 0.39  ± 0.02 | 0.08  ± 0.05 | 0.56  ± 0.23 | 0.06  ± 0.01 | 0.33  ± 0.07 | 0.06  ± 0.01 |
| 64 | 14.279 | 4-Hydroxyphenyl propionic acid |  | 0.24  ± 0.13 | 0.13  ± 0.01 | 0.15  ± 0.01 | 0.23  ± 0.04 | 0.19  ± 0.08 | 0.20  ± 0.03 | 0.13  ± 0.01 | 0.23  ± 0.05 | 0.13  ± 0.07 | 0.20  ± 0.01 | 0.16  ± 0.07 | 0.20  ± 0.03 |
| 65 | 14.574 | Vanillic acid |  | 0.41  ± 0.04 | 0.06  ± 0.01 | 0.39  ± 0.08 | 0.12  ± 0.03 | 0.41  ± 0.13 | 0.08  ± 0.03 | 0.33  ± 0.07 | 0.12  ± 0.03 | 0.29  ± 0.15 | 0.11  ± 0.01 | 0.38  ± 0.14 | 0.08  ± 0.02 |
| 66 | 14.636 | Azelaic acid/ Nonanedioic acid |  | 0.36  ± 0.06 | 0.08  ± 0.01 | 0.31  ± 0.06 | 0.24  ± 0.26 | 0.24  ± 0.06 | 0.19  ± 0.20 | 0.26  ± 0.11 | 0.09  ± 0.04 | 0.28  ± 0.07 | 0.09  ± 0.01 | 0.23  ± 0.02 | 0.06  ± 0.03 |
| Sum of Acids | | | | **44.67** | **25.43** | **46.36** | **23.68** | **45.68** | **24.28** | **48.85** | **25.63** | **45.92** | **22.66** | **47.31** | **22.87** |
| 1 | 4.247 | Propylene glycol | Alcohols | 0.58  ± 0.29 | 0.15  ± 0.01 | 1.20  ± 0.16 | 0.10  ± 0.02 | 0.82  ± 0.39 | 0.09  ± 0.02 | 1.09  ± 0.53 | 0.10  ± 0.02 | 0.53  ± 0.02 | 0.10  ± 0.01 | 1.21  ± 0.07 | 0.08  ± 0.02 |
| 3 | 4.855 | Propylene glycol |  | 0.19  ± 0.22 | 0.14  ± 0.00 | 0.80  ± 0.18 | 0.09  ± 0.02 | 0.39  ± 0.35 | 0.08  ± 0.01 | 0.63  ± 0.50 | 0.08  ± 0.02 | 0.17  ± 0.06 | 0.09  ± 0.005 | 0.81  ± 0.06 | 0.08  ± 0.02 |
| 5 | 5.043 | 1,3-Propanediol |  | 1.41  ± 0.08 | 2.30  ± 0.01 | 1.55  ± 0.04 | 1.51  ± 0.21 | 1.62  ± 0.35 | 1.41  ± 0.07 | 1.72  ± 0.15 | 1.42  ± 0.14 | 1.59  ± 0.17 | 1.59  ± 0.22 | 1.53  ± 0.19 | 1.17  ± 0.42 |
| 15 | 6.218 | Nonanol |  | 1.98  ± 0.20 | 0.68  ± 0.01 | 2.18  ± 0.05 | 0.51  ± 0.11 | 2.15  ± 0.23 | 0.32  ± 0.11 | 1.96  ± 0.27 | 0.50  ± 0.11 | 1.92  ± 0.24 | 0.45  ± 0.01 | 2.35  ± 0.12 | 0.39  ± 0.08 |
| 19 | 6.491 | Benzyl alcohol |  | 0.04  ± 0.04 | 0.07  ± 0.01 | 0.13  ± 0.02 | 0.07  ± 0.03 | 0.07  ± 0.10 | 0.06  ± 0.05 | 0.01  ± 0.01 | 0.06  ± 0.03 | 0.02  ± 0.01 | 0.07  ± 0.03 | 0.15  ± 0.06 | 0.06  ± 0.02 |
| 20 | 6.632 | 1,4-Butanediol |  | 0.32  ± 0.03 | 0.09  ± 0.004 | 0.33  ± 0.05 | 0.07  ± 0.02 | 0.33  ± 0.06 | 0.05  ± 0.005 | 0.37  ± 0.05 | 0.08  ± 0.02 | 0.40  ± 0.12 | 0.06  ± 0.003 | 0.34  ± 0.04 | 0.06  ± 0.01 |
| 23 | 7.565 | 2-Phenylethanol |  | 0.04  ± 0.04 | 0.07  ± 0.01 | 0.04  ± 0.01 | 0.08  ± 0.01 | 0.04  ± 0.004 | 0.06  ± 0.03 | 0.04  ± 0.01 | 0.08  ± 0.01 | 0.04  ± 0.004 | 0.08  ± 0.002 | 0.04  ± 0.02 | 0.09  ± 0.01 |
| 27 | 7.835 | Diethylene glycol |  | 1.20  ± 0.09 | 0.40  ± 0.01 | 1.15  ± 0.04 | 0.30  ± 0.10 | 1.29  ± 0.22 | 0.21  ± 0.02 | 1.34  ± 0.20 | 0.29  ± 0.09 | 1.25  ± 0.19 | 0.25  ± 0.01 | 1.29  ± 0.12 | 0.22  ± 0.04 |
| 32 | 8.338 | Glycerol |  | 1.42  ± 0.32 | 0.10  ± 0.001 | 1.34  ± 0.12 | 0.09  ± 0.03 | 1.18  ± 0.17 | 0.11  ± 0.09 | 1.03  ± 0.23 | 0.09  ± 0.03 | 1.13  ± 0.03 | 0.07  ± 0.01 | 1.35  ± 0.09 | 0.07  ± 0.01 |
| 60 | 12.951 | 3-Hydroxy-4-methoxybenzyl alcohol |  | 0.01  ± 0.005 | 0.07  ± 0.01 | 0.01  ± 0.01 | 0.15  ± 0.03 | 0.01  ± 0.01 | 0.11  ± 0.04 | 0.003  ± 0.004 | 0.14  ± 0.04 | 0.01  ± 0.01 | 0.13  ± 0.004 | 0.01  ± 0.01 | 0.13  ± 0.02 |
| 74 | 16.584 | 1-Hexadecanol |  | 0.16  ± 0.02 | 0.02  ± 0.0001 | 0.16  ± 0.04 | 0.04  ± 0.01 | 0.16  ± 0.05 | 0.02  ± 0.02 | 0.11  ± 0.01 | 0.04  ± 0.01 | 0.12  ± 0.07 | 0.03  ± 0.001 | 0.14  ± 0.03 | 0.03  ± 0.005 |
| 78 | 18.106 | 1-Octadecanol |  | 0.23  ± 0.32 | 0.13  ± 0.01 | 0.87  ± 0.14 | 0.15  ± 0.07 | 0.30  ± 0.45 | 0.09  ± 0.05 | 0.03  ± 0.01 | 0.12  ± 0.04 | 0.03  ± 0.02 | 0.12  ± 0.03 | 0.87  ± 0.27 | 0.07  ± 0.03 |
| Sum of Alcohols | | | | **7.59** | **4.23** | **9.76** | **3.13** | **8.37** | **2.61** | **8.34** | **2.99** | **7.22** | **3.06** | **10.09** | **2.45** |
| 35 | 8.493 | 2,4-Nonadienal | Aldehydes | 0.09  ± 0.02 | 0.04  ± 0.01 | 0.13  ± 0.08 | 0.02  ± 0.01 | 0.08  ± 0.02 | 0.25  ± 0.40 | 0.14  ± 0.10 | 0.01  ± 0.01 | 0.12  ± 0.08 | 0.01  ± 0.01 | 0.12  ± 0.07 | 0.01  ± 0.01 |
| 40 | 8.812 | 2,4-Decadienal |  | 0.17  ± 0.15 | 0.08  ± 0.01 | 0.42  ± 0.03 | 0.06  ± 0.01 | 0.24  ± 0.16 | 0.11  ± 0.07 | 0.25  ± 0.12 | 0.06  ± 0.02 | 0.12  ± 0.03 | 0.07  ± 0.01 | 0.49  ± 0.05 | 0.05  ± 0.03 |
| 57 | 11.705 | 3-Hydroxy-4-methoxybenzaldehyde |  | 0.50  ± 0.41 | 0.001  ± 0.001 | 0.08  ± 0.02 | 0.00  ± 0.00 | 0.26  ± 0.14 | 0.00  ± 0.00 | 1.23  ± 0.25 | 0.00  ± 0.00 | 0.21  ± 0.03 | 0.001  ± 0.001 | 0.08  ± 0.05 | 0.001  ± 0.001 |
| 69 | 15.607 | 7,10,-Hexadecadienal |  | 0.07  ± 0.05 | 0.03  ± 0.02 | 0.07  ± 0.02 | 0.03  ± 0.02 | 0.09  ± 0.07 | 0.01  ± 0.01 | 0.19  ± 0.16 | 0.03  ± 0.02 | 0.07  ± 0.03 | 0.01  ± 0.01 | 0.09  ± 0.11 | 0.01  ± 0.01 |
| 70 | 15.663 | Octadecenal |  | 0.09  ± 0.06 | 0.03  ± 0.01 | 0.06  ± 0.03 | 0.02  ± 0.01 | 0.10  ± 0.07 | 0.02  ± 0.001 | 0.19  ± 0.16 | 0.03  ± 0.02 | 0.08  ± 0.002 | 0.03  ± 0.03 | 0.10  ± 0.11 | 0.01  ± 0.001 |
| Sum of Aldehydes | | | | **0.91** | **0.17** | **0.76** | **0.14** | **0.77** | **0.39** | **1.99** | **0.12** | **0.60** | **0.12** | **0.88** | **0.09** |
| 34 | 8.47 | Butyric acid, benzyl ester | Esters | 0.03  ± 0.02 | 0.03  ± 0.01 | 0.03  ± 0.01 | 0.004  ± 0.001 | 0.05  ± 0.01 | 0.01  ± 0.01 | 0.18  ± 0.15 | 0.004  ± 0.003 | 0.07  ± 0.01 | 0.01  ± 0.003 | 0.05  ± 0.04 | 0.005  ± 0.001 |
| 46 | 9.483 | 3-Methyl benzoate |  | 0.07  ± 0.07 | 0.04  ± 0.0004 | 0.09  ± 0.03 | 0.10  ± 0.03 | 0.41  ± 0.22 | 0.05  ± 0.04 | 0.05  ± 0.03 | 0.08  ± 0.02 | 0.30  ± 0.06 | 0.08  ± 0.01 | 0.26  ± 0.37 | 0.08  ± 0.01 |
| 55 | 11.497 | 4- Methoxybenzoate |  | 0.23  ± 0.02 | 0.07  ± 0.002 | 0.24  ± 0.02 | 0.06  ± 0.02 | 0.26  ± 0.05 | 0.07  ± 0.05 | 0.29  ± 0.04 | 0.08  ± 0.05 | 0.30  ± 0.05 | 0.06  ± 0.01 | 0.26  ± 0.03 | 0.05  ± 0.01 |
| Sum of Esters | | | | **0.33** | **0.13** | **0.36** | **0.163** | **0.73** | **0.13** | **0.52** | **0.164** | **0.67** | **0.15** | **0.57** | **0.133** |
| 62 | 13.004 | Lauric acid | Fatty acids/ Esters | 0.21  ± 0.06 | 0.05  ± 0.01 | 0.14  ± 0.02 | 0.05  ± 0.01 | 0.17  ± 0.03 | 0.04  ± 0.001 | 0.20  ± 0.05 | 0.05  ± 0.03 | 0.25  ± 0.06 | 0.05  ± 0.01 | 0.21  ± 0.05 | 0.05  ± 0.001 |
| 67 | 15.13 | Myristic acid |  | 0.38  ± 0.01 | 0.13  ± 0.01 | 0.35  ± 0.02 | 0.11  ± 0.03 | 0.38  ± 0.06 | 0.09  ± 0.01 | 0.47  ± 0.07 | 0.10  ± 0.03 | 0.43  ± 0.05 | 0.10  ± 0.01 | 0.38  ± 0.04 | 0.08  ± 0.005 |
| 72 | 15.841 | n-Pentadecanoic acid |  | 0.01  ± 0.01 | 0.05  ± 0.01 | 0.00  ± 0.00 | 0.10  ± 0.01 | 0.004  ± 0.003 | 0.07  ± 0.06 | 0.00  ± 0.00 | 0.12  ± 0.02 | 0.003  ± 0.002 | 0.09  ± 0.02 | 0.003  ± 0.01 | 0.06  ± 0.03 |
| 75 | 17.065 | Palmitic acid |  | 7.00  ± 0.51 | 1.63  ± 0.26 | 4.89  ± 0.52 | 1.41  ± 0.36 | 4.79  ± 0.50 | 1.36  ± 0.64 | 6.06  ± 0.20 | 1.11  ± 0.34 | 7.23  ± 0.20 | 1.47  ± 0.25 | 5.21  ± 0.06 | 1.01  ± 0.003 |
| 77 | 17.973 | Margaric acid |  | 0.33  ± 0.03 | 0.09  ± 0.01 | 0.25  ± 0.04 | 0.07  ± 0.02 | 0.36  ± 0.05 | 0.06  ± 0.01 | 0.29  ± 0.11 | 0.08  ± 0.02 | 0.45  ± 0.07 | 0.08  ± 0.01 | 0.30  ± 0.07 | 0.06  ± 0.01 |
| 79 | 18.603 | Linoleic acid |  | 5.79  ± 2.02 | 0.68  ± 0.06 | 5.80  ± 1.29 | 1.04  ± 0.35 | 6.51  ± 0.97 | 0.84  ± 0.62 | 3.87  ± 1.68 | 0.45  ± 0.17 | 7.08  ± 2.00 | 0.52  ± 0.40 | 4.07  ± 2.15 | 0.44  ± 0.18 |
| 80 | 18.633 | Oleic acid |  | 5.89  ± 0.83 | 0.58  ± 0.12 | 4.85  ± 1.03 | 0.78  ± 0.37 | 6.08  ± 1.73 | 0.81  ± 0.61 | 6.64  ± 1.68 | 0.34  ± 0.09 | 6.39  ± 2.73 | 0.67  ± 0.14 | 4.78  ± 1.90 | 0.33  ± 0.03 |
| 81 | 18.844 | Stearic acid |  | 9.27  ± 0.79 | 2.38  ± 0.28 | 7.64  ± 0.18 | 1.86  ± 0.46 | 8.14  ± 0.57 | 1.46  ± 0.34 | 9.36  ± 0.70 | 1.63  ± 0.48 | 8.80  ± 0.34 | 1.66  ± 0.20 | 7.64  ± 0.63 | 1.37  ± 0.14 |
| 82 | 19.371 | Linoleic acid |  | 0.23  ± 0.07 | 0.06  ± 0.0004 | 0.30  ± 0.20 | 0.12  ± 0.05 | 0.33  ± 0.08 | 0.21  ± 0.29 | 0.41  ± 0.16 | 0.06  ± 0.02 | 0.34  ± 0.02 | 0.05  ± 0.02 | 0.27  ± 0.06 | 0.04  ± 0.02 |
| 83 | 20.362 | Glyceryl linoleate |  | 0.24  ± 0.03 | 0.06  ± 0.03 | 0.13  ± 0.04 | 0.08  ± 0.02 | 0.30  ± 0.07 | 0.10  ± 0.07 | 0.49  ± 0.09 | 0.03  ± 0.01 | 0.32  ± 0.05 | 0.08  ± 0.03 | 0.17  ± 0.10 | 0.03  ± 0.01 |
| 84 | 20.492 | Arachidic acid |  | 0.25  ± 0.15 | 0.11  ± 0.05 | 0.32  ± 0.26 | 0.07  ± 0.03 | 0.45  ± 0.19 | 0.28  ± 0.33 | 0.35  ± 0.15 | 0.09  ± 0.01 | 0.22  ± 0.06 | 0.11  ± 0.02 | 0.26  ± 0.16 | 0.09  ± 0.01 |
| 85 | 20.752 | 9, 12-Octadecadienoic acid, methyl ester/ Linoleic acid |  | 1.12  ± 1.69 | 0.08  ± 0.01 | 0.12  ± 0.08 | 0.11  ± 0.05 | 0.31  ± 0.07 | 0.21  ± 0.23 | 0.52  ± 0.12 | 0.05  ± 0.03 | 0.52  ± 0.20 | 0.12  ± 0.04 | 0.29  ± 0.27 | 0.05  ± 0.02 |
| 86 | 21.713 | 1-Monopalmitin/ Glyceryl-1-palmitate |  | 0.48  ± 0.16 | 0.21  ± 0.09 | 0.51  ± 0.08 | 0.14  ± 0.10 | 0.51  ± 0.02 | 0.12  ± 0.06 | 0.51  ± 0.07 | 0.09  ± 0.04 | 0.50  ± 0.01 | 0.12  ± 0.01 | 0.44  ± 0.08 | 0.14  ± 0.03 |
| 87 | 22.014 | Docosanoic acid |  | 0.03  ± 0.02 | 0.003  ± 0.003 | 0.02  ± 0.02 | 0.01  ± 0.01 | 0.03  ± 0.03 | 0.005  ± 0.004 | 0.05  ± 0.03 | 0.00  ± 0.00 | 0.01  ± 0.01 | 0.002  ± 0.001 | 0.01  ± 0.01 | 0.002  ± 0.002 |
| 89 | 23.235 | Sebacic acid, di-(2-ethylhexyl) ester/ Decanedioic acid, di-(2-ethylhexyl) ester |  | 0.86  ± 0.08 | 0.29  ± 0.03 | 0.77  ± 0.03 | 0.19  ± 0.06 | 0.84  ± 0.15 | 0.17  ± 0.07 | 0.91  ± 0.10 | 0.17  ± 0.05 | 0.92  ± 0.08 | 0.16  ± 0.002 | 0.81  ± 0.10 | 0.17  ± 0.03 |
| Sum of Fatty acids/ Esters | | | | **32.08** | **6.39** | **26.10** | **6.16** | **29.20** | **5.82** | **30.13** | **4.37** | **33.48** | **5.28** | **24.82** | **3.93** |
| 22 | 7.463 | Valine | Nitrogenous/ Amino acids | 0.66  ± 0.21 | 0.02  ± 0.002 | 0.46  ± 0.22 | 0.13  ± 0.03 | 0.18  ± 0.12 | 0.03  ± 0.03 | 0.38  ± 0.10 | 0.16  ± 0.09 | 0.16  ± 0.01 | 0.07  ± 0.01 | 0.43  ± 0.34 | 0.04  ± 0.03 |
| 24 | 7.705 | Ethanolamine |  | 1.07  ± 0.09 | 0.34  ± 0.001 | 1.08  ± 0.03 | 0.21  ± 0.09 | 1.00  ± 0.14 | 0.15  ± 0.03 | 1.18  ± 0.07 | 0.21  ± 0.04 | 0.82  ± 0.01 | 0.19  ± 0.01 | 1.08  ± 0.19 | 0.16  ± 0.05 |
| 30 | 8.178 | Nicotinic acid/ Vitamin B3 |  | 1.48  ± 1.86 | 0.73  ± 0.01 | 3.55  ± 0.58 | 0.53  ± 0.24 | 1.94  ± 2.52 | 0.60  ± 0.10 | 0.38  ± 0.04 | 0.52  ± 0.20 | 0.52  ± 0.03 | 0.62  ± 0.11 | 4.29  ± 1.54 | 0.42  ± 0.35 |
| 31 | 8.274 | L-Leucine |  | 1.17  ± 0.36 | 0.02  ± 0.002 | 0.71  ± 0.27 | 0.16  ± 0.03 | 0.37  ± 0.23 | 0.05  ± 0.03 | 0.66  ± 0.13 | 0.19  ± 0.12 | 0.36  ± 0.02 | 0.08  ± 0.01 | 0.90  ± 0.69 | 0.06  ± 0.04 |
| 36 | 8.493 | Nicotinic acid/ Vitamin B3 |  | 0.22  ± 0.03 | 0.02  ± 0.0002 | 0.19  ± 0.02 | 0.02  ± 0.01 | 0.20  ± 0.02 | 0.01  ± 0.01 | 0.15  ± 0.02 | 0.03  ± 0.01 | 0.17  ± 0.03 | 0.02  ± 0.002 | 0.20  ± 0.05 | 0.02  ± 0.004 |
| 38 | 8.585 | Isoleucine |  | 0.54  ± 0.19 | 0.01  ± 0.001 | 0.34  ± 0.14 | 0.09  ± 0.02 | 0.19  ± 0.09 | 0.03  ± 0.01 | 0.30  ± 0.05 | 0.10  ± 0.06 | 0.17  ± 0.01 | 0.05  ± 0.01 | 0.40  ± 0.25 | 0.03  ± 0.02 |
| 42 | 8.917 | Nicotinic acid/ Vitamin B3 |  | 0.17  ± 0.09 | 0.05  ± 0.0005 | 0.17  ± 0.04 | 0.05  ± 0.01 | 0.13  ± 0.06 | 0.02  ± 0.02 | 0.11  ± 0.03 | 0.04  ± 0.01 | 0.12  ± 0.001 | 0.04  ± 0.005 | 0.15  ± 0.03 | 0.04  ± 0.01 |
| 44 | 9.197 | Uracil |  | 1.06  ± 0.12 | 1.37  ± 0.03 | 1.00  ± 0.07 | 2.31  ± 0.40 | 1.23  ± 0.28 | 1.60  ± 0.38 | 0.84  ± 0.15 | 2.24  ± 0.56 | 0.87  ± 0.48 | 2.07  ± 0.19 | 1.03  ± 0.37 | 1.45  ± 0.49 |
| 49 | 10.055 | Thymine |  | 0.34  ± 0.04 | 0.22  ± 0.01 | 0.28  ± 0.02 | 0.36  ± 0.08 | 0.34  ± 0.10 | 0.22  ± 0.09 | 0.28  ± 0.06 | 0.34  ± 0.09 | 0.26  ± 0.13 | 0.31  ± 0.02 | 0.30  ± 0.11 | 0.23  ± 0.06 |
| 51 | 10.519 | Indole |  | 0.003  ± 0.01 | 18.22  ± 0.07 | 0.04  ± 0.01 | 22.62  ± 3.96 | 0.33  ± 0.19 | 18.79  ± 1.82 | 0.01  ± 0.01 | 22.53  ± 4.90 | 1.21  ± 0.48 | 20.99  ± 2.66 | 0.02  ± 0.01 | 20.90  ± 1.56 |
| 53 | 10.793 | N-Nitroso-diethanolamine |  | 0.16  ± 0.04 | 0.03  ± 0.003 | 0.17  ± 0.04 | 0.05  ± 0.01 | 0.17  ± 0.09 | 0.03  ± 0.03 | 0.08  ± 0.03 | 0.05  ± 0.01 | 0.08  ± 0.05 | 0.05  ± 0.01 | 0.19  ± 0.09 | 0.03  ± 0.02 |
| 54 | 11.011 | L-Aspartic acid |  | 0.14  ± 0.08 | 0.001  ± 0.001 | 0.24  ± 0.09 | 0.03  ± 0.01 | 0.03  ± 0.02 | 0.01  ± 0.01 | 0.08  ± 0.03 | 0.03  ± 0.02 | 0.02  ± 0.003 | 0.02  ± 0.01 | 0.14  ± 0.11 | 0.01  ± 0.01 |
| 56 | 11.58 | Pyroglutamic acid |  | 0.38  ± 0.21 | 0.03  ± 0.01 | 0.59  ± 0.21 | 0.03  ± 0.01 | 0.20  ± 0.07 | 0.02  ± 0.01 | 0.33  ± 0.03 | 0.03  ± 0.02 | 0.26  ± 0.02 | 0.02  ± 0.01 | 0.46  ± 0.26 | 0.02  ± 0.003 |
| 58 | 12.762 | Glutamic acid |  | 0.14  ± 0.16 | 0.00  ± 0.00 | 0.35  ± 0.16 | 0.00  ± 0.00 | 0.07  ± 0.04 | 0.00  ± 0.00 | 0.15  ± 0.02 | 0.002  ± 0.004 | 0.07  ± 0.01 | 0.00  ± 0.00 | 0.25  ± 0.21 | 0.00  ± 0.00 |
| 59 | 12.842 | Phenylalanine |  | 0.37  ± 0.05 | 0.001  ± 0.001 | 0.26  ± 0.06 | 0.06  ± 0.02 | 0.17  ± 0.08 | 0.02  ± 0.01 | 0.21  ± 0.05 | 0.06  ± 0.03 | 0.09  ± 0.02 | 0.03  ± 0.01 | 0.27  ± 0.17 | 0.02  ± 0.01 |
| 63 | 13.778 | Indole, 5-hydroxy |  | 0.00  ± 0.00 | 0.04  ± 0.01 | 0.00  ± 0.00 | 0.05  ± 0.01 | 0.00  ± 0.00 | 0.03  ± 0.03 | 0.00  ± 0.00 | 0.05  ± 0.01 | 0.00  ± 0.00 | 0.05  ± 0.002 | 0.00  ± 0.00 | 0.05  ± 0.01 |
| 68 | 15.445 | Adenine |  | 3.00  ± 0.90 | 0.02  ± 0.004 | 3.69  ± 0.47 | 0.06  ± 0.004 | 3.95  ± 1.78 | 0.04  ± 0.02 | 2.13  ± 0.45 | 0.06  ± 0.02 | 2.59  ± 2.02 | 0.07  ± 0.01 | 3.43  ± 1.26 | 0.05  ± 0.01 |
| 71 | 15.806 | Vitamin B6/ Pyridoxine |  | 1.36  ± 0.25 | 0.13  ± 0.005 | 1.31  ± 0.18 | 0.38  ± 0.04 | 1.79  ± 0.37 | 0.46  ± 0.28 | 0.69  ± 0.14 | 0.32  ± 0.07 | 1.38  ± 0.29 | 0.31  ± 0.03 | 1.10  ± 0.71 | 0.25  ± 0.06 |
| 73 | 16.424 | Indole-3-acetic acid |  | 0.30  ± 0.06 | 0.03  ± 0.0005 | 0.25  ± 0.01 | 0.04  ± 0.004 | 0.24  ± 0.09 | 0.03  ± 0.02 | 0.25  ± 0.01 | 0.04  ± 0.01 | 0.16  ± 0.11 | 0.04  ± 0.002 | 0.23  ± 0.03 | 0.03  ± 0.003 |
| 76 | 17.545 | Indole-3-propionic acid |  | 0.002  ± 0.004 | 0.35  ± 0.05 | 0.002  ± 0.004 | 0.64  ± 0.11 | 0.002  ± 0.003 | 0.51  ± 0.15 | 0.00  ± 0.00 | 0.61  ± 0.17 | 0.00  ± 0.00 | 0.61  ± 0.03 | 0.001  ± 0.003 | 0.61  ± 0.14 |
| 88 | 22.22 | Adenosine |  | 0.19  ± 0.05 | 0.00  ± 0.00 | 0.22  ± 0.03 | 0.00  ± 0.00 | 0.22  ± 0.02 | 0.00  ± 0.00 | 0.13  ± 0.04 | 0.001  ± 0.001 | 0.17  ± 0.07 | 0.0003  ± 0.001 | 0.16  ± 0.03 | 0.001  ± 0.002 |
| 92 | 26.065 | 3,5-Diethyl-2-methylpyrazine |  | 0.33  ± 0.10 | 0.12  ± 0.06 | 0.44  ± 0.09 | 0.11  ± 0.02 | 0.30  ± 0.23 | 0.24  ± 0.35 | 0.38  ± 0.005 | 0.07  ± 0.05 | 0.31  ± 0.11 | 0.02  ± 0.01 | 0.24  ± 0.06 | 0.04  ± 0.02 |
| Sum of Nitrogenous/ Amino acids | | | | **13.07** | **21.76** | **15.37** | **27.93** | **13.06** | **22.90** | **8.69** | **27.66** | **9.78** | **25.68** | **15.26** | **24.45** |
| 4 | 5.002 | Phenol | Phenols | 0.24  ± 0.01 | 41.63  ± 0.67 | 0.35  ± 0.02 | 38.58  ± 7.86 | 1.32  ± 0.18 | 43.07  ± 3.68 | 0.19  ± 0.04 | 38.81  ± 7.86 | 1.45  ± 0.60 | 42.78  ± 2.14 | 0.36  ± 0.26 | 45.87  ± 1.65 |
| 41 | 8.899 | Pyrocatechol |  | 0.08  ± 0.11 | 0.01  ± 0.001 | 0.02  ± 0.02 | 0.004  ± 0.001 | 0.03  ± 0.01 | 0.02  ± 0.03 | 0.05  ± 0.004 | 0.01  ± 0.004 | 0.02  ± 0.00 | 0.03  ± 0.04 | 0.03  ± 0.02 | 0.01  ± 0.01 |
| Sum of Phenols | | | | **0.33** | **41.64** | **0.37** | **38.58** | **1.35** | **43.10** | **0.24** | **38.81** | **1.46** | **42.81** | **0.39** | **45.88** |
| 47 | 10.005 | Hydroquinone | Quinones | 0.07  ± 0.02 | 0.02  ± 0.001 | 0.06  ± 0.02 | 0.04  ± 0.02 | 0.06  ± 0.03 | 0.03  ± 0.02 | 0.06  ± 0.01 | 0.05  ± 0.01 | 0.03  ± 0.03 | 0.04  ± 0.004 | 0.06  ± 0.01 | 0.04  ± 0.01 |
| Sum of Quinones | | | | **0.07** | **0.02** | **0.06** | **0.04** | **0.06** | **0.03** | **0.06** | **0.05** | **0.03** | **0.04** | **0.06** | **0.04** |
| 93 | 27.184 | Campesterol | Sterols | 0.15  ± 0.01 | 0.07  ± 0.01 | 0.19  ± 0.03 | 0.04  ± 0.02 | 0.13  ± 0.08 | 0.14  ± 0.21 | 0.19  ± 0.15 | 0.04  ± 0.02 | 0.17  ± 0.11 | 0.03  ± 0.01 | 0.15  ± 0.04 | 0.03  ± 0.01 |
| 94 | 28.206 | Beta-Sitosterol |  | 0.80  ± 0.13 | 0.16  ± 0.08 | 0.65  ± 0.46 | 0.14  ± 0.02 | 0.66  ± 0.30 | 0.61  ± 0.82 | 0.99  ± 0.28 | 0.16  ± 0.05 | 0.66  ± 0.06 | 0.18  ± 0.04 | 0.46  ± 0.04 | 0.12  ± 0.08 |
| Sum of Sterols | | | | **0.95** | **0.23** | **0.84** | **0.18** | **0.78** | **0.75** | **1.18** | **0.19** | **0.83** | **0.21** | **0.61** | **0.14** |
| 90 | 23.977 | Tocopherol-δ | Vitamins | 0.00  ± 0.00 | 0.00  ± 0.00 | 0.00  ± 0.00 | 0.00  ± 0.00 | 0.00  ± 0.00 | 0.00  ± 0.00 | 0.00  ± 0.00 | 0.00  ± 0.00 | 0.00  ± 0.00 | 0.00  ± 0.00 | 0.00  ± 0.00 | 0.00  ± 0.00 |
| 91 | 24.69 | Tocopherol |  | 0.00  ± 0.00 | 0.00  ± 0.00 | 0.01  ± 0.01 | 0.001  ± 0.001 | 0.00  ± 0.00 | 0.00  ± 0.00 | 0.00  ± 0.00 | 0.00  ± 0.00 | 0.00  ± 0.00 | 0.00  ± 0.00 | 0.01  ± 0.01 | 0.002  ± 0.003 |
| Sum of vitamins | | | | **0.00** | **0.00** | **0.01** | **0.00** | **0.00** | **0.00** | **0.00** | **0.00** | **0.00** | **0.00** | **0.01** | **0.002** |

**Suppl. Table S3:** Metabolites profiling in COFU, COOU, COFI, COOI, SFFU, SFOU, SFFI, SFOI, SOFU and SOFI oils using HPLC-MS/MS, for codes explanation, refer to **Table 1**

* immonium ion, MM: Microbial Metabolites

| NO | RT | Name | | M+H (error, ppm) | | Molecular formula | | Fragmentation | | COFU | | COOU | | COFI | | COOI | | SFFU | | SFOU | | SFFI | | SFOI | | SOFU | | SOFI | | Class | | Ref | |  |
| --- | --- | --- | --- | --- | --- | --- | --- | --- | --- | --- | --- | --- | --- | --- | --- | --- | --- | --- | --- | --- | --- | --- | --- | --- | --- | --- | --- | --- | --- | --- | --- | --- | --- | --- |
|  | 0.73 | | Valyl-valine (Val-Val) | | 217.1544 (-1.23) | | C_10_H_20_N_2_O_3_ | | 118, 114 | | - | | - | | - | | - | | + | | - | | - | | - | | - | | - | | Dipeptide | | ^1^ | |
|  | 0.76 | | Glycyl-tyrosine (Gly-Tyr) | | 239.1029 (1.11) | | C_11_H_14_N_2_O_4_ | | 193, 182, 165, 147, 136 (Tyr*), 123, 120, 119 | | - | | - | | - | | - | | + | | - | | - | | - | | - | | - | | Dipeptide | | ^2^ | |
|  | 0.89 | | Asparaginyl glutamine (Asn-Gln) | | 261.1189  (-1.71) | | C_9_H_16_N_4_O_5_ | | 243, 255, 223, 162, 144 | | - | | - | | + | | + | | - | | - | | + | | + | | - | | + | | Dipeptide (MM) | | ^3^ | |
|  | 0.9 | | Leucine-Glutamine (Leu-Gln) | | 260.1600 (-1.85) | | C_11_H_21_N_3_O_4_ | | 200, 157, 143, 136 | | - | | - | | - | | - | | + | | - | | - | | - | | - | | - | | Dipeptide | | ^3,4^ | |
|  | 0.95 | | Glutamyl-valyl-proline (Glu-Val-Pro) | | 344.1820 (1012) | | C_15_H_25_N_3_O_6_ | | 245, 244, 229, 227, 211, 209, 201, 182, 129, 121, 116, 102(Glu*) | | - | | - | | - | | - | | + | | - | | - | | - | | - | | - | | Tripeptide | |  | |
|  | 1 | | N2-(D-1-Carboxyethyl)-L-lysine;Lysopine | | 219.1336 (-2.0) | | C_9_H_18_N_2_O_4_ | | 201, 173, 154, 132, 130, 112, 110, 105 | | - | | - | | - | | - | | + | | + | | - | | - | | + | | - | | Alanine derivative | | ^5^ | |
|  | 1.07 | | Alanine-leucine  (Ala-Leu) | | 203.1388 (-1.07) | | C_9_H_18_N_2_O_3_ | | 132, 120, 113 | | - | | - | | - | | - | | + | | - | | - | | - | | - | | - | | Dipeptide | | ^3^ | |
|  | 1.15 | | Glutamine-leucine (Gln-Leu) | | 261.1447 (0.77) | | C_11_H_20_N_2_O_5_ | | 197, 152, 132, 130, 102 (Glu*) | | - | | - | | - | | - | | + | | - | | - | | - | | - | | - | | Dipeptide | | ^3^ | |
|  | 1.22 | | Prolyl-proline  (Pro-Pro) | | 213.1228 (-2.66) | | C_10_H_16_N_2_O_3_ | | 185, 172, 170, 157, 139, 125, 114, 110, 100 | | + | | + | | + | | - | | - | | + | | + | | + | | + | | - | | Dipeptide | | ^6^ | |
|  | 1.24 | | Glycyl-prolyl-leucyl-glycine (Gly-Pro-Leu-Gly) | | 343.1970 (-1.75) | | C_15_H_26_N_4_O_5_ | | 265, 261, 252, 240, 155, 127 | | - | | - | | - | | - | | + | | - | | - | | - | | - | | - | | Tripeptide | | ^6^ | |
|  | 1.32 | | Serinyl-Phenylalanine (Ser-Phe) | | 253.1180 (-1.12) | | C_12_H_16_N_2_O_4_ | | 207, 178, 166, 149, 120 (Phe*), 107 | | - | | - | | - | | - | | + | | - | | - | | - | | - | | - | | Dipeptide | | ^7^ | |
|  | 1.4 | | Glycyl-phenylalanine (Gly-Phe) | | 223.1070 (-2.77) | | C_11_H_14_N_2_O_3_ | | 177, 166, 149, 140, 131, 120 (Phe*), 103 | | - | | - | | - | | - | | + | | - | | - | | - | | - | | - | | Dipeptide | | ^8,9^ | |
|  | 1.43 | | Alanyl-phenylalanine (Ala-Phe) | | 237.1230 (-1.55) | | C_12_H_16_N_2_O_3_ | | 175, 166, 149, 120 (Phe*) | | - | | - | | - | | - | | + | | - | | - | | - | | - | | - | | Dipeptide | | ^9,10^ | |
|  | 1.45 | | Valyl leucine (Val-Leu) | | 231.1700 (-1.38) | | C_11_H_22_N_2_O_3_ | | 162, 144, 132, 106 | | - | | - | | - | | - | | + | | - | | - | | - | | - | | - | | Dipeptide | | ^7^ | |
|  | 1.54 | | Tyrosyl-valyl-proline (Tyr-Val-Pro) | | 378.2017 (-1.71) | | C_19_H_27_N_3_O_5_ | | 341, 329, 279, 263, 235, 164, 136 (Tyr*), 116 | | - | | - | | - | | - | | + | | - | | - | | - | | - | | - | | Tripeptide | |  | |
|  | 1.64 | | Valyl-leucyl-proline  (Val−Leu−Pro) | | 328.224 (-2.08) | | C_16_H_29_N_3_O_4_ | | 299, 294, 285, 238, 229, 213, 185, 142, 129, 116 | | - | | - | | - | | - | | + | | - | | - | | - | | - | | - | | Tripeptide | | ^3^ | |
|  | 1.82 | | Cyclo(tyr-tyr) | | 327.1346 (2.03) | | C_18_H_18_N_2_O_4_ | | 310, 299, 281, 254, 221, 203, 164, 136 (Tyr*), 119, 107 | | + | | - | | - | | - | | - | | - | | - | | - | | - | | - | | Cyclic peptide | | ^11^ | |
|  | 1.84 | | Valyl-leucyl-serine  (Val-Leu**-**Ser) | | 318.2022  (-0.46) | | C_14_H_27_N_3_O_5_ | | 187, 173, 159, 132, 114 | | - | | - | | - | | - | | + | | - | | - | | - | | - | | - | | Tripeptide | |  | |
|  | 1.97 | | leucyl-prolyl-valyl-prolyl-glutamine (leu-pro-val-pro-gln) | | 553.3341 (-0.58) | | C_26_H_44_N_6_O_7_ | | 536, 535, 440, 407, 379, 343, 310, 292, 244, 226, 211, 197, 183, 171, 169, 147 | | - | | - | | - | | - | | + | | - | | - | | - | | - | | - | | Polypeptide | |  | |
|  | 2.02 | | Leucyl-leucine (Leu−Lue) | | 245.1854 (-2.32) | | C_12_H_24_N_2_O_3_ | | 182, 132 | | - | | - | | - | | - | | + | | - | | - | | - | | - | | - | | Dipeptide | | ^8^ | |
|  | 2.05 | | Tetrahydroharman-3-carboxylic acid | | 231.1127 (-0.45) | | C_13_H_14_N_2_O_2_ | | 188, 168, 158, 154, 146, 130 | | - | | - | | - | | - | | + | | - | | - | | - | | - | | - | | Indole alkaloids | | ^12^ | |
|  | 2.08 | | Phenylalanyl-proline (Phe-Pro) | | 263.1395 (0.76) | | C_14_H_18_N_2_O_3_ | | 229, 217, 190, 187, 143, 136, 120 (Phe*), 116, 103 | | + | | + | | + | | + | | + | | + | | + | | + | | + | | + | | Dipeptide | | ^6^ | |
|  | 2.19 | | Tetrahydroharman-3-carboxylic acid | | 231.1127 (-0.45) | | C_13_H_14_N_2_O_2_ | | 188, 168, 158, 154, 146, 130 | | - | | - | | - | | - | | + | | - | | - | | - | | - | | - | | Indole alkaloids | | ^12^ | |
|  | 2.23 | | Cyclo-(L-Pro-L-Leu), Gancidin W, Maculosin 6 | | 211.1438 (0.67) | | C_11_H_18_N_2_O_2_ | | 183, 166, 155, 154, 153, 138, 127, 125, 114, 110, 109 | | + | | - | | + | | + | | + | | + | | + | | + | | + | | + | | Cyclic peptide | | ^13,14^ | |
|  | 2.26 | | Valyl-leucyl-prolyl-valyl-prolyl-glutamine (Val-leu-pro-val-pro-gln) | | 652.4017  (-1.74) | | C_31_H_53_N_7_O_8_ | | 630, 619, 584, 553, 506, 495, 440, 409, 381, 310, 244, 227, 213, 197, 169 | | - | | - | | - | | - | | + | | - | | - | | - | | - | | - | | Polypeptide | | GNPS | |
|  | 2.42 | | Phenylalanyl-leucine (Phe-Leu) | | 279.1699 (0.78) | | C_15_H_22_N_2_O_3_ | | 260, 226, 207, 170, 136, 132, 120 (Phe*), 103 | | - | | - | | - | | - | | + | | - | | - | | - | | - | | - | | Dipeptide | | ^8^ | |
|  | 2.44 | | isoleucyl-prolyl-isoleucine (Ile-Pro-Ile), diprotin A | | 342.2384 (-0.97) | | C_17_H_31_N_3_O_4_ | | 299, 287, 278, 229, 211, 183, 129, 126, 114 | | - | | - | | - | | - | | + | | - | | - | | - | | - | | - | | Tripeptide | | ^15^ | |
|  | 2.57 | | Valyl-isoleucyl-leucine  (Val-Ile-Leu) | | 344.2535 (-2.56) | | C_17_H_33_N_3_O_4_ | | 269,249, 245, 262, 185 | | - | | - | | - | | - | | + | | - | | - | | - | | - | | - | | Tripeptide | |  | |
|  | 2.53 | | Tyrosyl-isoleucyl-prolyl-valine | | 491.2858 (-1024) | | C_25_H_38_N_4_O_6_ | | 263, 235, 229, 183, 136 (Tyr*) | | - | | - | | - | | - | | + | | - | | - | | - | | - | | - | | Polypeptide | |  | |
|  | 2.78 | | leucyl-prolyl-phenylalanine (leu-Pro-Phe ) | | 376.2258 (7.2) | | [C_20_H_29_N_3_O_4_](https://pubchem.ncbi.nlm.nih.gov/#query=C20H29N3O4) | | 245, 229, 217, 183, 120 (Phe*) | | - | | - | | - | | - | | + | | - | | - | | - | | - | | - | | Tripeptide | |  | |
|  | 2.94 | | Cyclo-(Tyr-Phe) | | 311.1365 (-8.09) | | C_18_H_18_N_2_O_3_ | | 283,266, 238, 205, 177, 136 (Tyr*), 120 (Pro*), 107 | | + | | - | | + | | - | | + | | - | | - | | - | | + | | - | | Cyclic peptide | | ^16^ | |
|  | 3.1 | | Cyclo-(Phe-Pro), Maculosine 2 | | 245.1291 (2.62) | | C_14_H_16_N_2_O_2_ | | 228, 217, 189, 172, 154, 148, 131, 120 (Phe*) | | + | | + | | + | | + | | + | | + | | + | | + | | + | | + | | Cyclic peptide | | ^16^ | |
|  | 3.21 | | Glycyl-prolyl-phenylalanyl-prolyl-isoleucine (Gly-Pro-Phe-Pro-Ile) | | 530.2955 (-3.41) | | C_27_H_39_N_5_O_6_ | | 512, 473, 399, 376, 302, 274, 229, 155, 127, 120 (Phe*) | | - | | - | | - | | - | | + | | - | | - | | - | | - | | - | | Polypeptide | |  | |
|  | 3.64 | | Cyclo-(Leu-leu) | | 227.1755 (0.21) | | C_12_H_22_N_2_O_2_ | | 199, 182, 154, 114 | | + | | + | | + | | + | | + | | + | | + | | + | | + | | + | | Cyclic peptide | | ^16^ | |
|  | 3.80 | | Cyclo-(Leu-Phe) | | 261.1579 (-0.84) | | C_15_H_20_N_2_O_2_ | | 233, 216, 188, 120 (Phe*), 114 | | + | | + | | + | | - | | + | | + | | + | | - | | + | | + | | Cyclic peptide | | ^17^ | |
|  | 3.9 | | N(3)-Fumaramoyl-2,3-Diaminopropanoic Acid | | 202.0816 (-3.27) | | C_7_H_11_N_3_O_4_ | | 177, 161, 159, 131, 125, 117, 115, 105, 103 | | - | | - | | + | | + | | - | | - | | + | | + | | - | | - | | Monoacylglycerols (MM) | | ^18^ | |
|  | 4.03 | | Cyclo-(Phe-Trp) | | 334.1544 (-1.80) | | C_20_H_19_N_3_O_2_ | | 170, 159 (Trp*), 142, 132, 120 (Phe*), 132, 130, 117 | | + | | + | | - | | - | | + | | + | | - | | - | | + | | - | | Cyclic peptide | | ^17^ | |
|  | 5.38 | | Acetyl-*β-*carboline | | 211.0859 (-3.26) | | C_13_H_10_N_2_O | | 193, 169, 167, 149, 115 | | + | | - | | + | | - | | + | | - | | + | | - | | + | | - | | Indole alkaloids | | ^19^ | |
|  | 5.52 | | 3-phenyl-9H-pyrido[3,4-b] indole | | 245.1074 (0.38) | | C_17_H_12_N_2_ | | 244, 243, 218, 217,159 | | - | | - | | + | | + | | - | | - | | + | | + | | - | | + | | Indole alkaloids  (MM) | |  | |
|  | 5.58 | | 1-(4-methylphenyl)-9H-pyrido[3,4-b] indole | | 259.1226 (-2.6) | | C_18_H_14_N_2_ | | 244, 232, 217, 187, 142, 115 | | - | | - | | + | | + | | - | | - | | + | | + | | - | | + | | Indole alkaloids (MM) | |  | |
|  | 6.33 | | C17 Sphinganine | | 288.2891 (-2.10) | | C_17_H_37_NO_2_ | | 260, 252, 228 | | - | | - | | + | | + | | - | | - | | + | | - | | - | | + | | Sphingolipid | | ^1^ | |
|  | 6.89 | | 2-phenyl-1-(9H-pyrido[3,4-b] indol-1-yl) ethanone, Eudistomin T | | 287.1181 (0.73) | | C_19_H_14_N_2_O | | 286, 272, 269, 259, 244,243, 232, 217,155, 154, 142, 115 | | - | | - | | + | | + | | - | | - | | + | | + | | - | | + | | Indole alkaloids (MM) | | ^20^ | |
|  | 7.49 | | 2-phenyl-1-(9H-pyrido[3,4-b]indol-1-yl) ethanone, Eudistomin T | | 287.1179 (0.03) | | C_19_H_14_N_2_O | | 259, 244, 232, 217, 170, 142, 130, 115 | | - | | - | | + | | + | | - | | - | | + | | + | | - | | + | | Indole alkaloids (MM) | | ^20^ | |
|  | 7.62 | | 1-(4-methylphenyl)-9H-pyrido[3,4-b]indole | | 259.1226 (-2.2) | | C_18_H_14_N_2_ | | 244, 232, 217, 187, 142, 115 | | - | | - | | + | | + | | - | | - | | + | | + | | - | | + | | Indole alkaloids (MM) | |  | |
|  | 7.92 | | 1-(4-methylphenyl)-9H-pyrido[3,4-b] indole | | 259.1226 (-1.33) | | C_18_H_14_N_2_ | | 244, 232, 217, 187, 142, 115 | | - | | - | | + | | + | | - | | - | | + | | + | | - | | + | | Indole alkaloids (MM) | |  | |
|  | 9.42 | | Vernolic acid | | 297.2415 (-3.09) | | C_18_H_32_O_3_ | | 281, 179, 153, 117, 109, 105 | | - | | + | | - | | + | | - | | - | | - | | - | | - | | - | | Fatty acid | | ^21^ | |
|  | 10.81 | | Palmitamide | | 256.2644  (3.54) | | C_16_H_33_NO | | 215, 200,174, 170,137, 116, 111,102, 100 | | - | | + | | - | | + | | - | | - | | - | | - | | - | | - | | Fatty acid | | ^22^ | |
|  | 10.95 | | Glyceryl palmitate | | 331.2835 (-2.37) | | C_19_H_38_O_4_ | | 313, 272, 109 | | - | | - | | - | | - | | - | | - | | - | | + | | - | | - | | Fatty acid | | ^22^ | |
|  | 11.14 | | Octadecadienoic acid (18:2) (linoleic acid) | | 281.2467 | | C_18_H_32_O_2_ | | 183, 101, 151, 119, 109, 107, 105 | | - | | + | | + | | + | | - | | - | | - | | - | | - | | - | | Fatty acid | | ^23^ | |
|  | 11.95 | | Diacetylsphingosine | | 408.3085 (0.27) (M+Na) | | C_22_H_43_NO_4_ | | 362, 266, 250, 221, 206, 192, 178, 164, 150, 136, 122, 108 | | + | | + | | - | | - | | + | | + | | - | | - | | - | | - | | Sphingolipid | | ^24^ | |
|  | 11.96 | | Oleic acid | | 283.2626 (-1.96) | | C_18_H_34_O_2_ | | 200, 172,141, 135, 125,121, 109 | | + | | + | | - | | - | | + | | + | | - | | - | | + | | - | | Fatty acid | |  | |
|  | 12.22 | | Diacetylsphingosine | | 408.3085 (0.27) (M+Na) | | C_22_H_43_NO_4_ | | 362, 266, 250, 221, 206, 192, 178, 164, 150, 136, 122, 108 | | + | | + | | - | | - | | + | | + | | - | | - | | - | | - | | Sphingolipid | | ^24^ | |
|  | 12.26 | | Eicosenamide | | 310.3102 (-0.77) | | C_20_H_39_NO | | 275, 268,153, 135, 125, 113, 111, 109, 100 | | - | | - | | - | | - | | + | | - | | + | | - | | + | | + | | Fatty acid | | ^22^ | |
|  | 12.43 | | N-pentadecylcyclohexanecarboxamide,  Erucamide | | 338.3418 (0.17) | | C_22_H_43_NO | | 321, 303, 226, 212, 195, 184, 177, 163, 156, 142 | | - | | - | | - | | + | | - | | - | | - | | + | | - | | - | | Fatty acid | | ^25^ | |
|  | 14.01 | | 3,4-Dehydro-alpha-tocopherol | | 429.3721  (-1.41) | | C_29_H_48_O_2_ | | 414, 373,317, 215, 205, 191, 165, 123 | | - | | + | | - | | - | | - | | + | | - | | - | | - | | + | | Tocopherol | |  | |

# **List of Supplementary Figures**


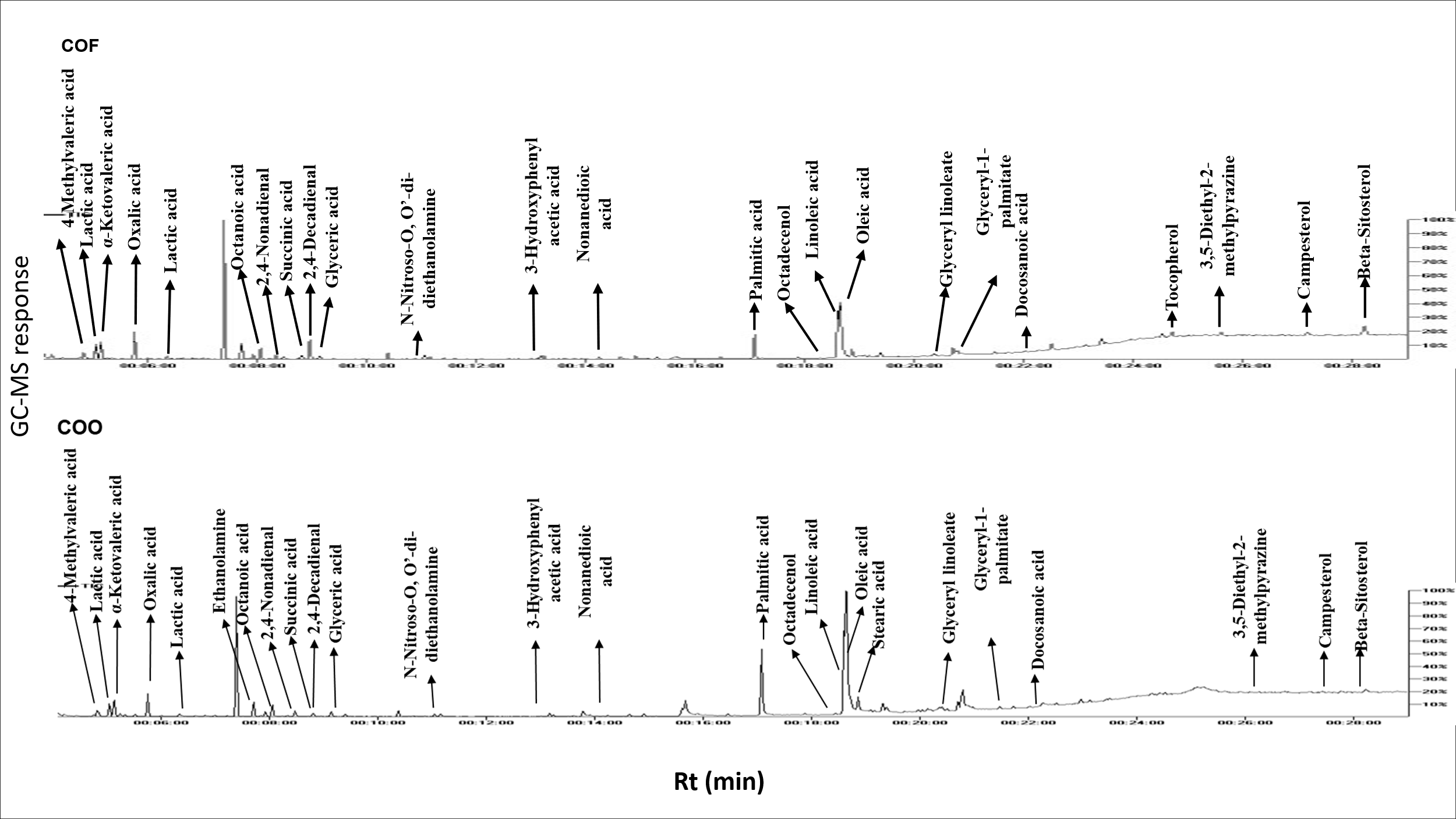


**Suppl. Fig. S1-A:** Representative GC-MS chromatograms of silylated metabolites in the fresh corn oil (COF) and oxidized corn oil (COO).

**
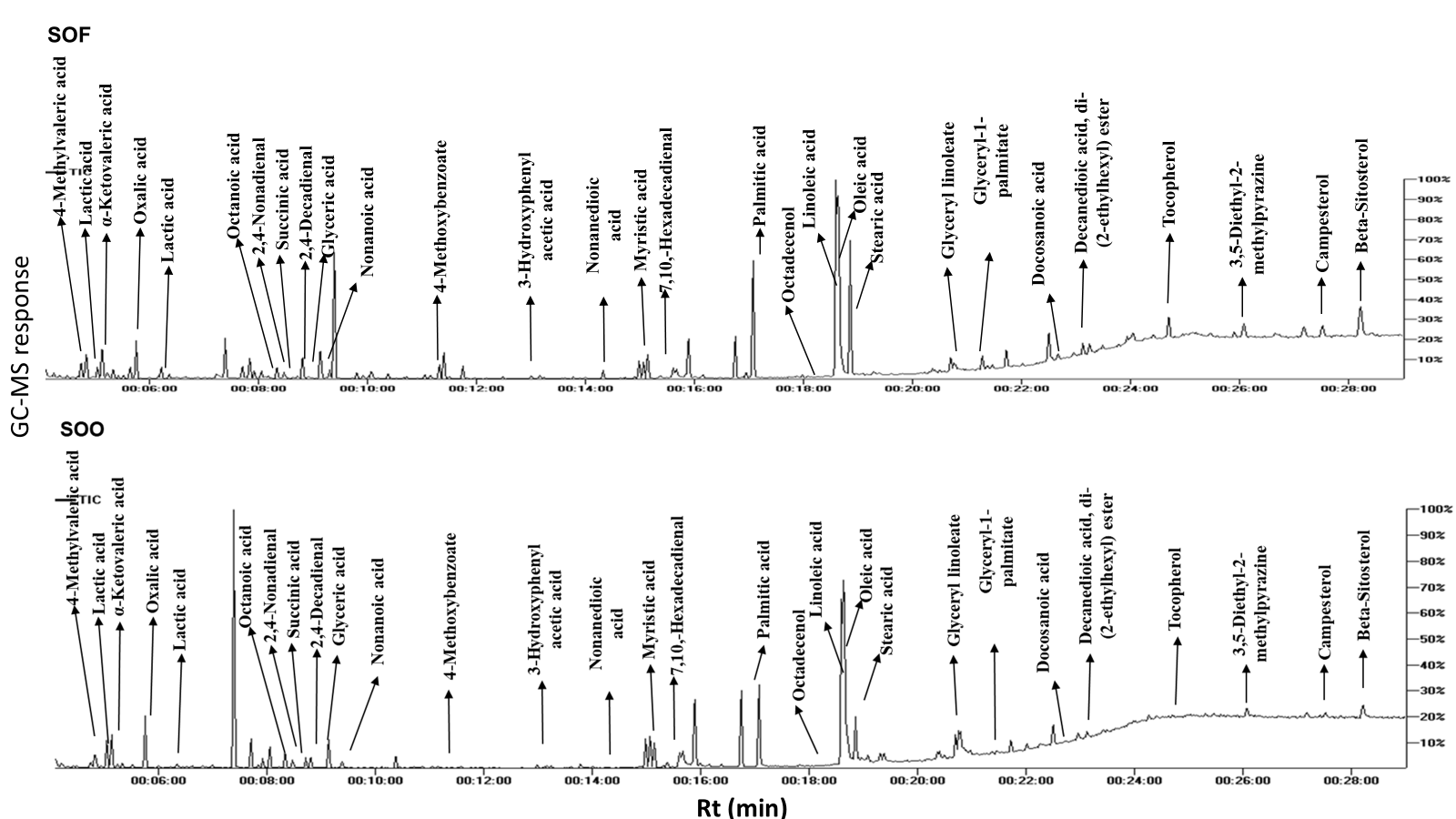
**

**Suppl. Fig. S1-B:** Representative GC-MS chromatograms of silylated metabolites in the fresh sesame oil (SOF), oxidized sesame oil (SOO) and fresh sunflower oil (SFF).

**
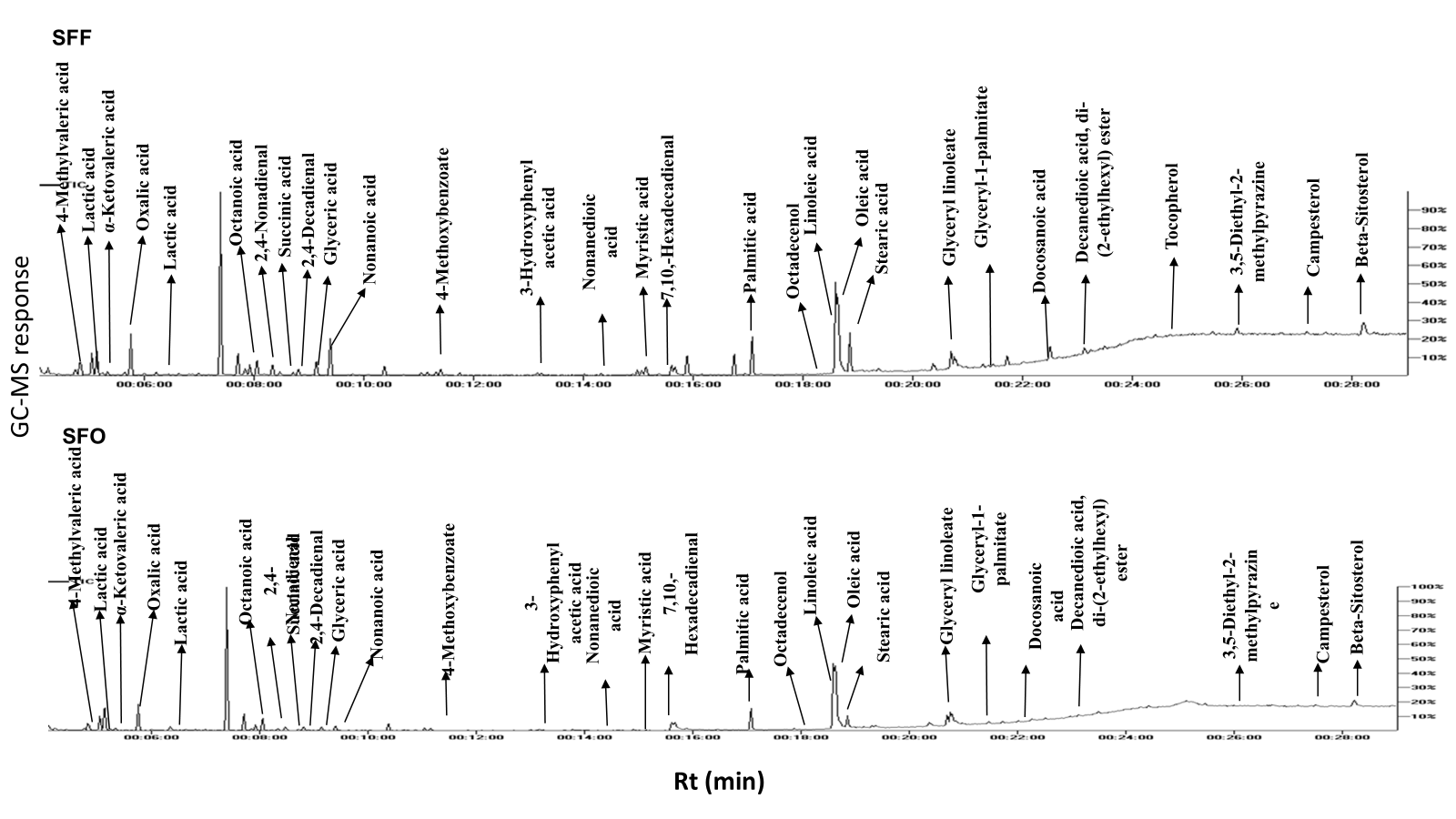
**

**Suppl. Fig. S1-C:** Representative GC-MS chromatograms of silylated metabolites in fresh sunflower oil (SFF) and oxidized sunflower oil (SFO) samples.


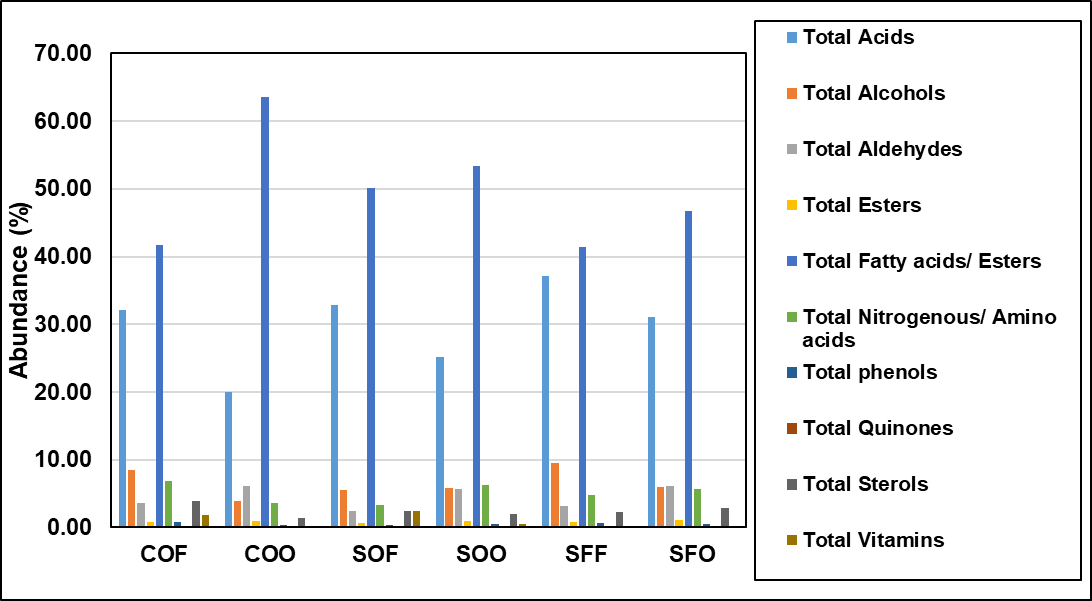


**Suppl. Fig. S2:** The relative percentile levels of all the detected metabolite classes in the fresh corn oil (COF), oxidized corn oil (COO), fresh sesame oil (SOF), oxidized sesame oil (SOO), fresh sunflower oil (SFF) and oxidized sunflower oil (SFO) samples. Data represent metabolite profiles obtained from a single exploratory experiment; therefore, statistical analysis, error bars, and significance testing were not applied.


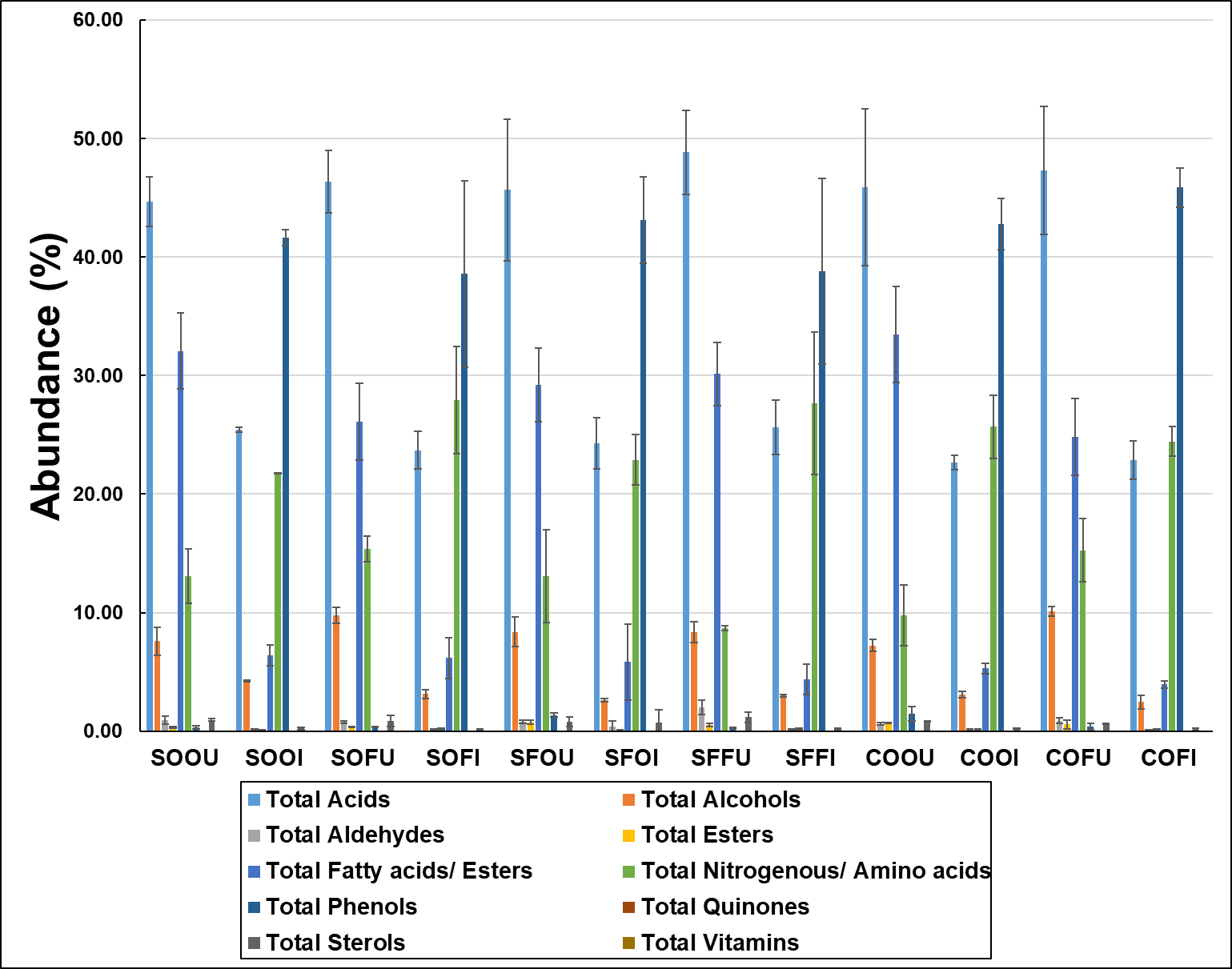


**Suppl. Fig. S3:** The relative percentile levels of all the detected metabolite classes in the oxidized uninoculated sesame oil (SOOU), oxidized inoculated sesame oil (SOOI), fresh uninoculated sesame oil (SOFU), fresh inoculated sesame oil (SOFI), oxidized uninoculated sunflower oil (SFOU), oxidized inoculated sunflower oil (SFOI), fresh uninoculated sunflower oil (SFFU), fresh inoculated sunflower oil (SFFI), oxidized uninoculated corn oil (COOU), oxidized inoculated corn oil (COOI), fresh uninoculated corn oil (COFU), and fresh inoculated corn oil (COFI) samples (n= 3), where the error bars represent the standard deviation (St. dev.). For codes explanation, refer to **Table 2**.


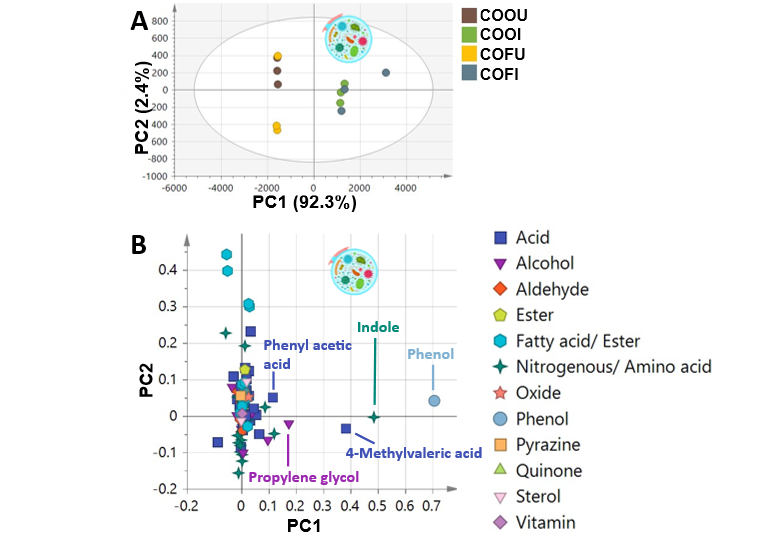


**Suppl. Fig. S4:** GC-MS based PCA of corn oils’ samples. (A) Score plot of PC1 *vs.* PC2 scores. (B) Loading plot for PC1 & PC2 contributing metabolites and their assignments. The metabolome clusters are located at distinct positions in two-dimensional space described by two vectors of principal component PC1 = 92.3% and PC2 = 2.4%.

(Oxidized uninoculated corn oil (COOU), oxidized inoculated corn oil (COOI), fresh uninoculated corn oil (COFU), and fresh inoculated corn oil (COFI) samples. For codes explanation, refer to **Table 2**).


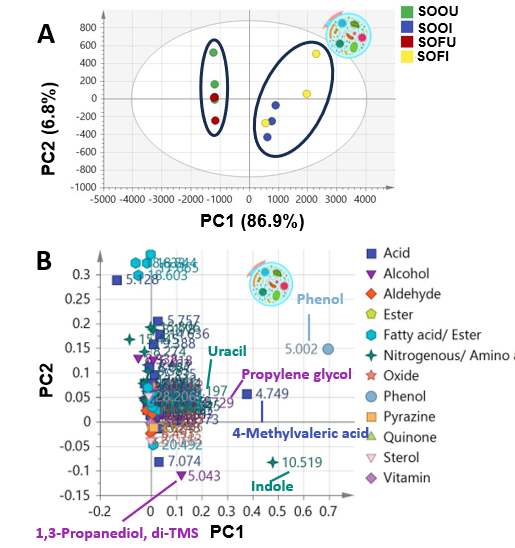


**Suppl. Fig. S5:** GC-MS based HCA and PCA of all sesame oils’ samples. (A) Score plot of PC1 *vs.* PC2 scores. (B) Loading plot for PC1 & PC2 contributing metabolites and their assignments. The metabolome clusters are located at distinct positions in two-dimensional space described by two vectors of principal component PC1 = 86.9% and PC2 = 6.8%. It should be noted that these ellipses are handmade and do not denote statistical significance.

(Oxidized uninoculated sesame oil (SOOU), oxidized inoculated sesame oil (SOOI), fresh uninoculated sesame oil (SOFU), and fresh inoculated sesame oil (SOFI). For codes explanation, refer to **Table 2**).


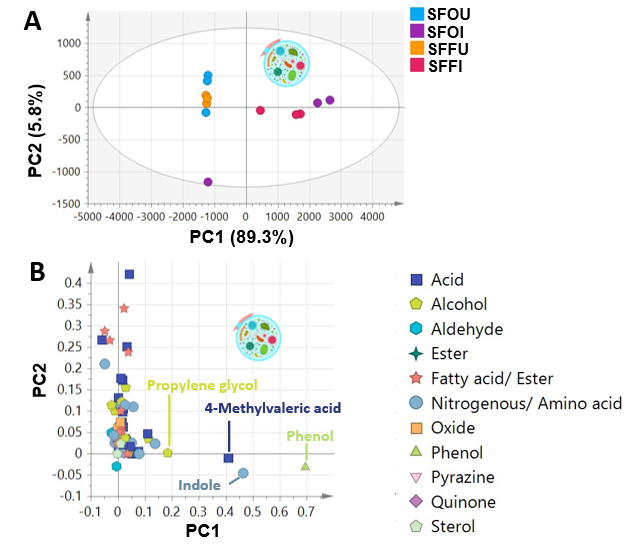


**Suppl. Fig. S6:** GC-MS based PCA of all sunflower oils’ samples. (A) Score plot of PC1 *vs.* PC2 scores. (B) Loading plot for PC1 & PC2 contributing metabolites and their assignments. The metabolome clusters are located at distinct positions in two-dimensional space described by two vectors of principal component PC1 = 89.3% and PC2 = 5.8%.

(Oxidized uninoculated sunflower oil (SFOU), oxidized inoculated sunflower oil (SFOI), fresh uninoculated sunflower oil (SFFU), and fresh inoculated sunflower oil (SFFI). For codes explanation, refer to **Table 2**).

**Suppl. Fig. S7:** GC-MS based OPLS-DA score plot derived from modelling of oxidized uninoculated corn oils’ (COOU) samples against fresh uninoculated corn oils’ (COFU) samples (A). The respective S-loading plot (B) shows the covariance p[1] against the correlation p(cor)[1] of the variables of the discriminating component of the OPLS-DA model. Cut-off values of P<0.05 were used; selected variables are highlighted in the S-plot with identifications.


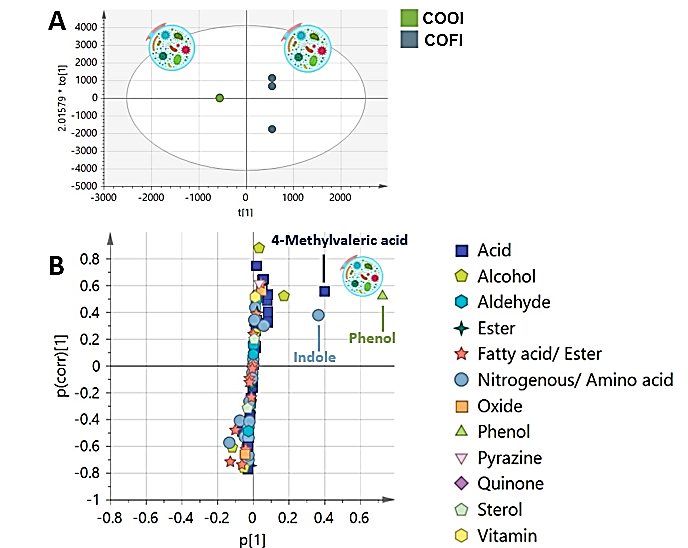


**Suppl. Fig. S8:** GC-MS based OPLS-DA score plot derived from modelling of oxidized inoculated corn oils’ (COOI) samples against fresh inoculated corn oils’ (COFI) samples (A). The respective S-plot (B) shows the covariance p[1] against the correlation p(cor)[1] of the variables of the discriminating component of the OPLS-DA model. Cut-off values of *p*<0.05 were used; selected variables are highlighted in the S-plot with identifications.

**Suppl. Fig. S9:** GC-MS based OPLS-DA score plot derived from modelling of oxidized uninoculated sesame oils’ (SOOU) samples against fresh uninoculated sesame oils’ (SOFU) samples (A). The respective S-plot (B) shows the covariance p[1] against the correlation p(cor)[1] of the variables of the discriminating component of the OPLS-DA model. Cut-off values of *p*<0.05 were used; selected variables are highlighted in the S-plot with identifications.


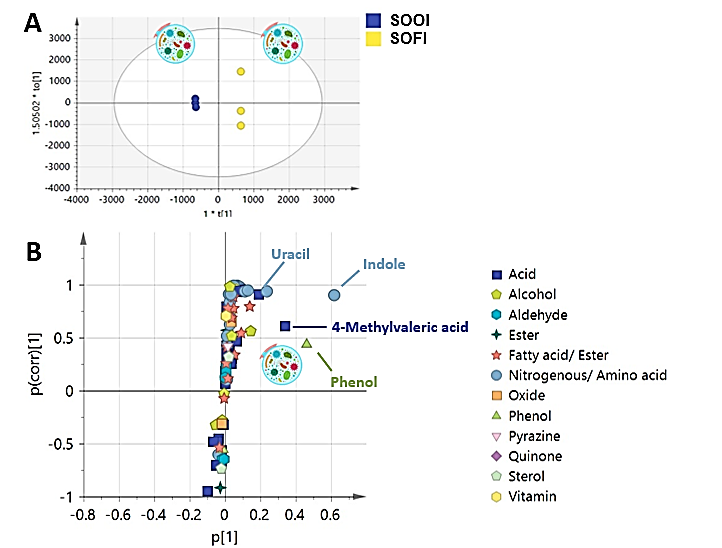


**Suppl. Fig. S10:** GC-MS based OPLS-DA score plot derived from modelling of oxidized inoculated sesame oils’ (SOOI) samples against fresh inoculated sesame oils’ (SOFI) samples (A). The respective S-plot (B) shows the covariance p[1] against the correlation p(cor)[1] of the variables of the discriminating component of the OPLS-DA model. Cut-off values of *p*<0.05 were used; selected variables are highlighted in the S-plot with identifications.


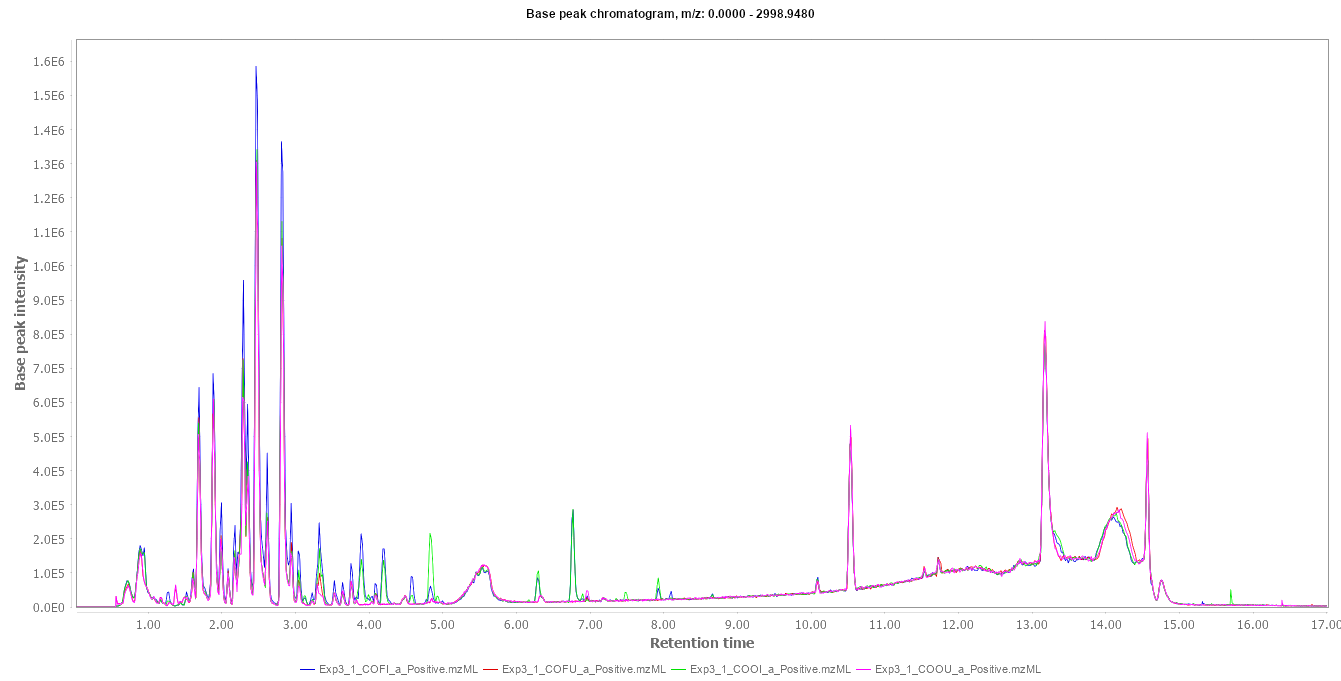

 **Suppl. Fig. S11A:** Base peak chromatogram (BPC) of fresh and oxidized corn seeds oils samples in absence and presence of *ex-vivo* gut microbiome culture

**
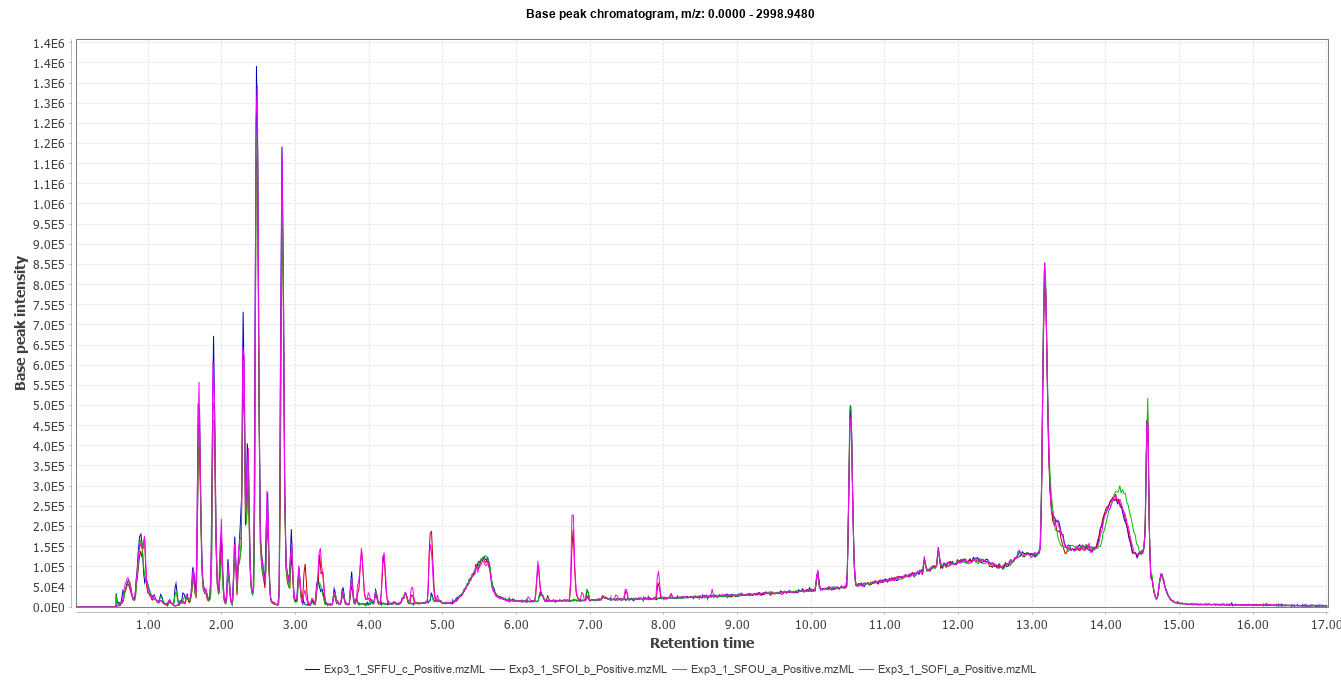
**

**Suppl. Fig. S11 B:** Base peak chromatogram (BPC) of fresh and oxidized sunflower seeds oils samples in absence and presence of *ex-vivo* gut microbiome culture.


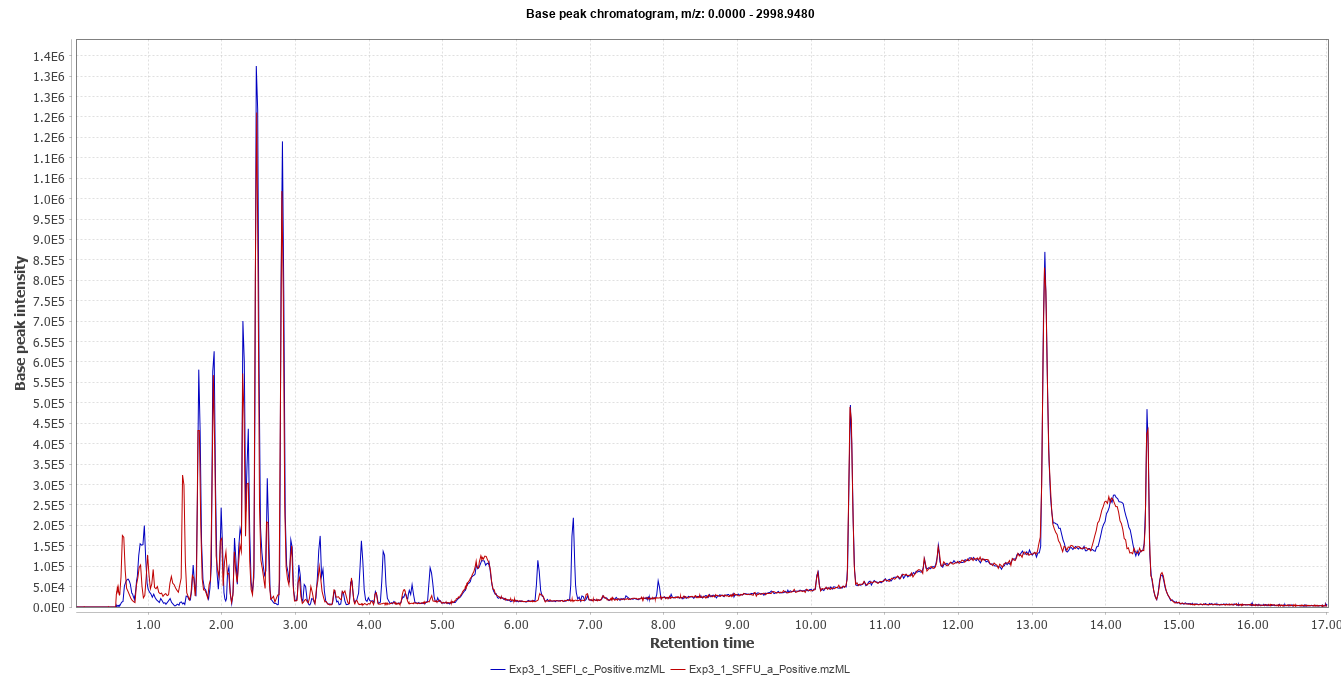

**Suppl. Fig. S11 C:** Base peak chromatogram (BPC) of fresh sesame seeds oil sample in absence and presence of *ex-vivo* gut microbiome culture.


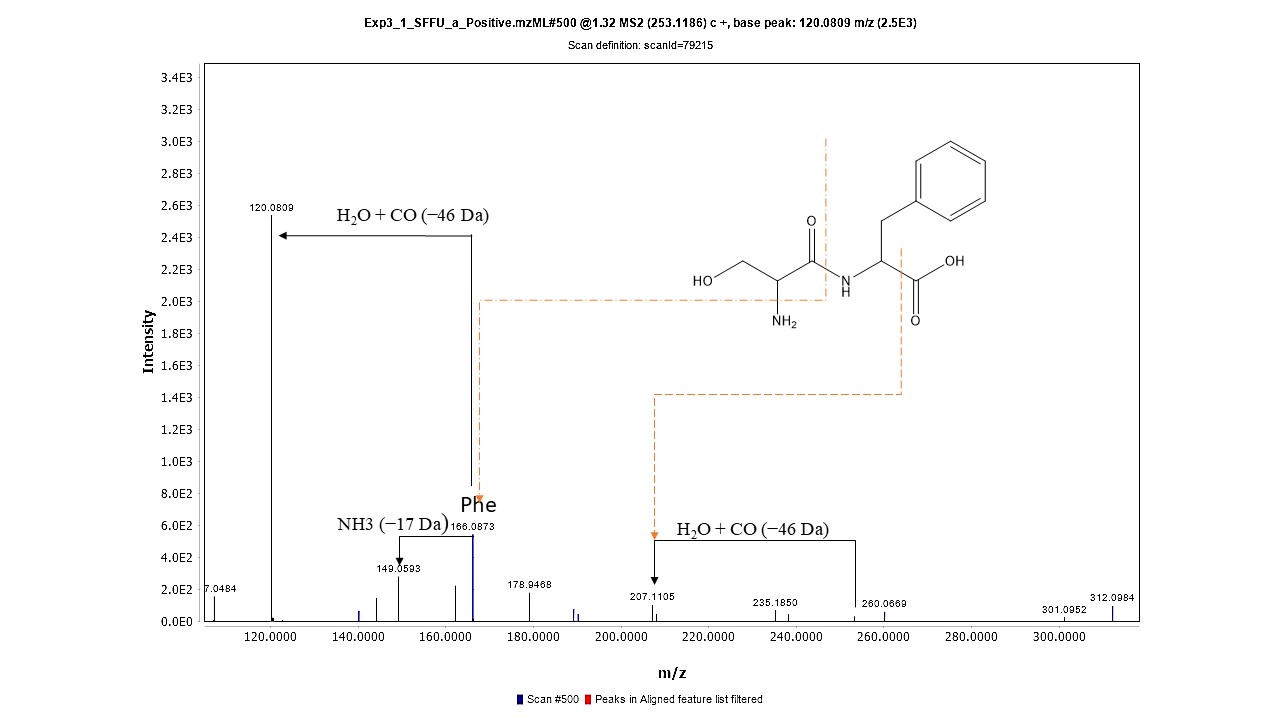


**Suppl. Fig. S12:** MS^2^ spectrum of 11, [M+H]^+^ at *m/z* 253.1180, Serinyl-Phenylalanine


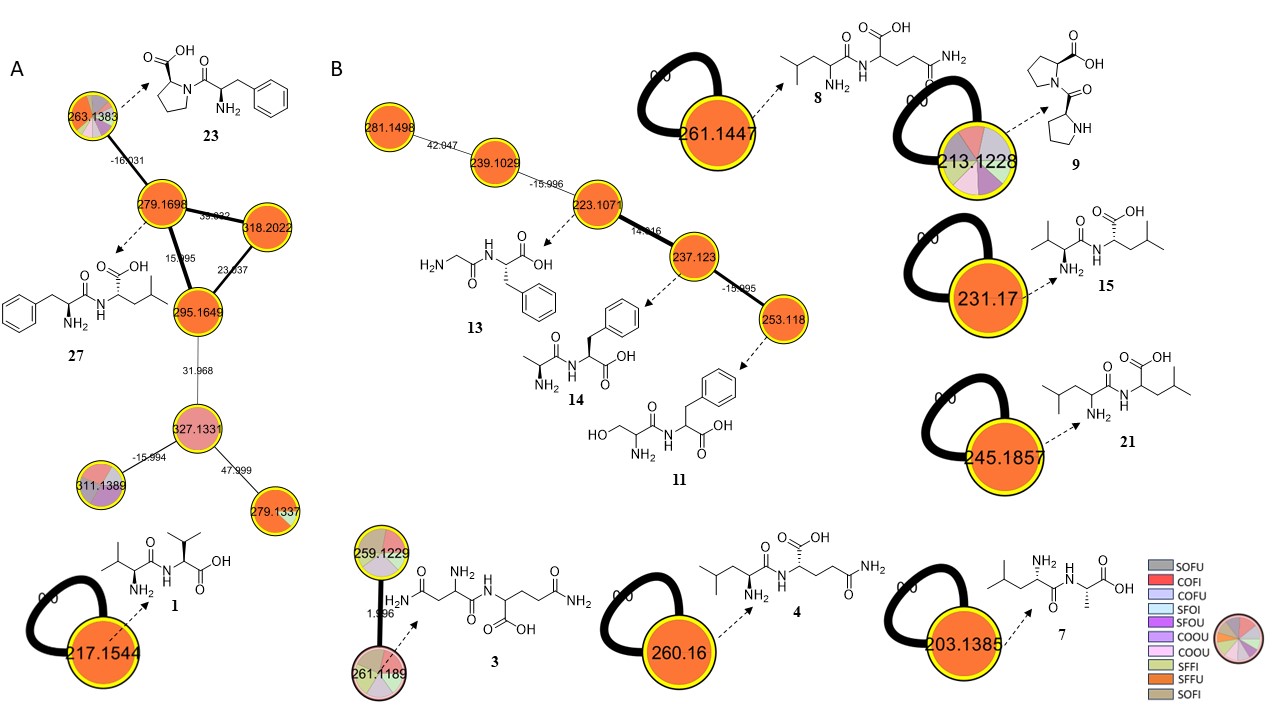


**Suppl. Fig. S13.** Annotated dipeptides and their distribution in the FBMN in all the tested samples


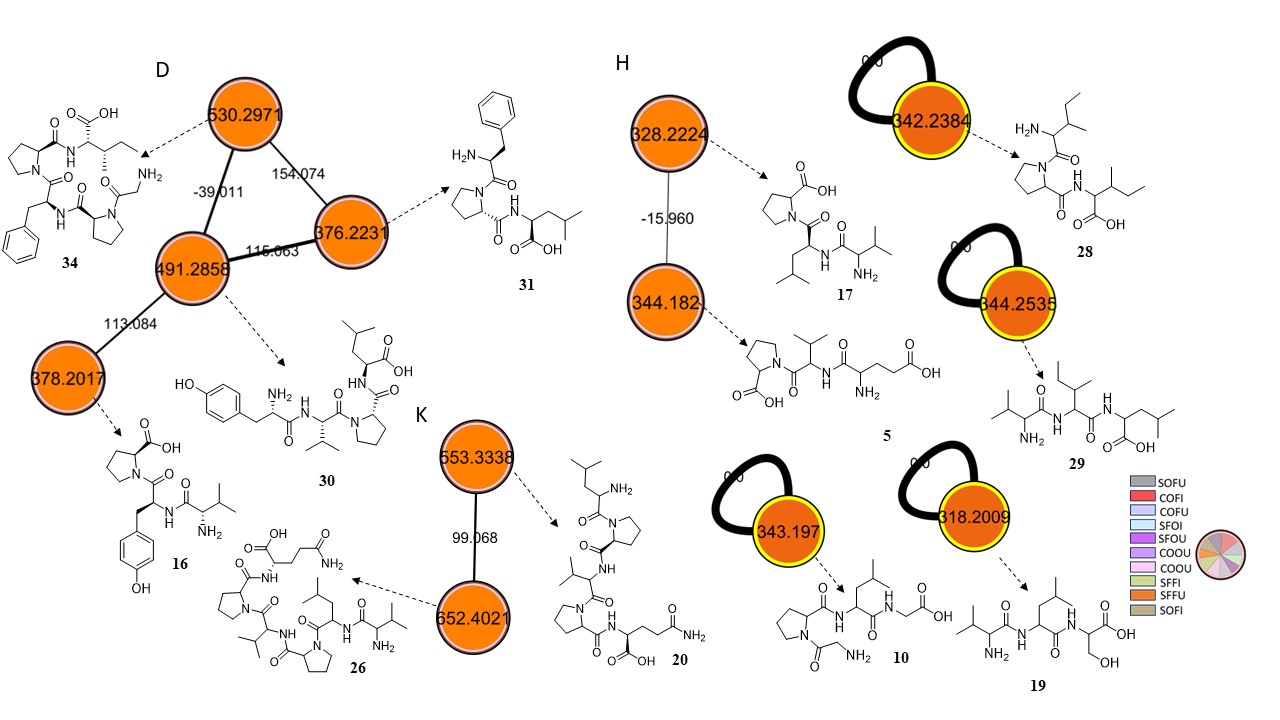


**Suppl. Fig. S14.** Annotated tripeptides and polypeptides and their distribution in the FBMN


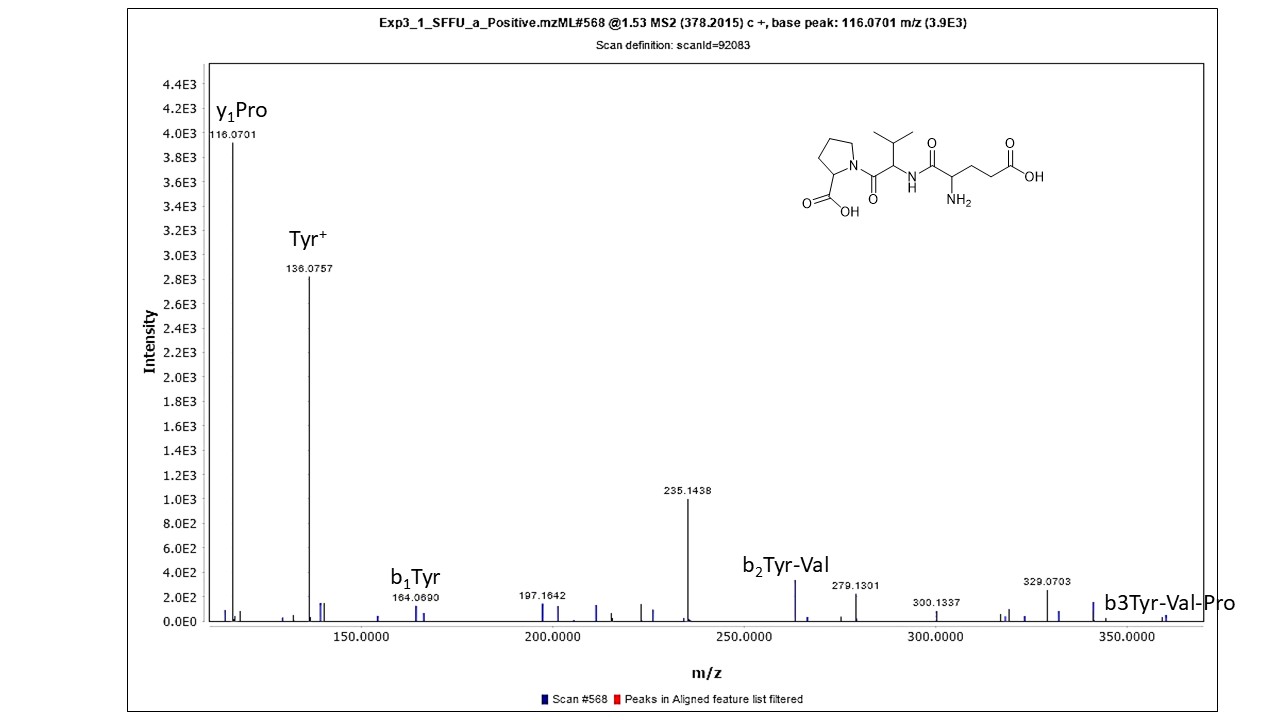


**Suppl. Fig. S15:** MS^2^ spectrum of 15, [M+H]^+^ at *m/z* 378.2017, Tyrosyl-valyl-proline


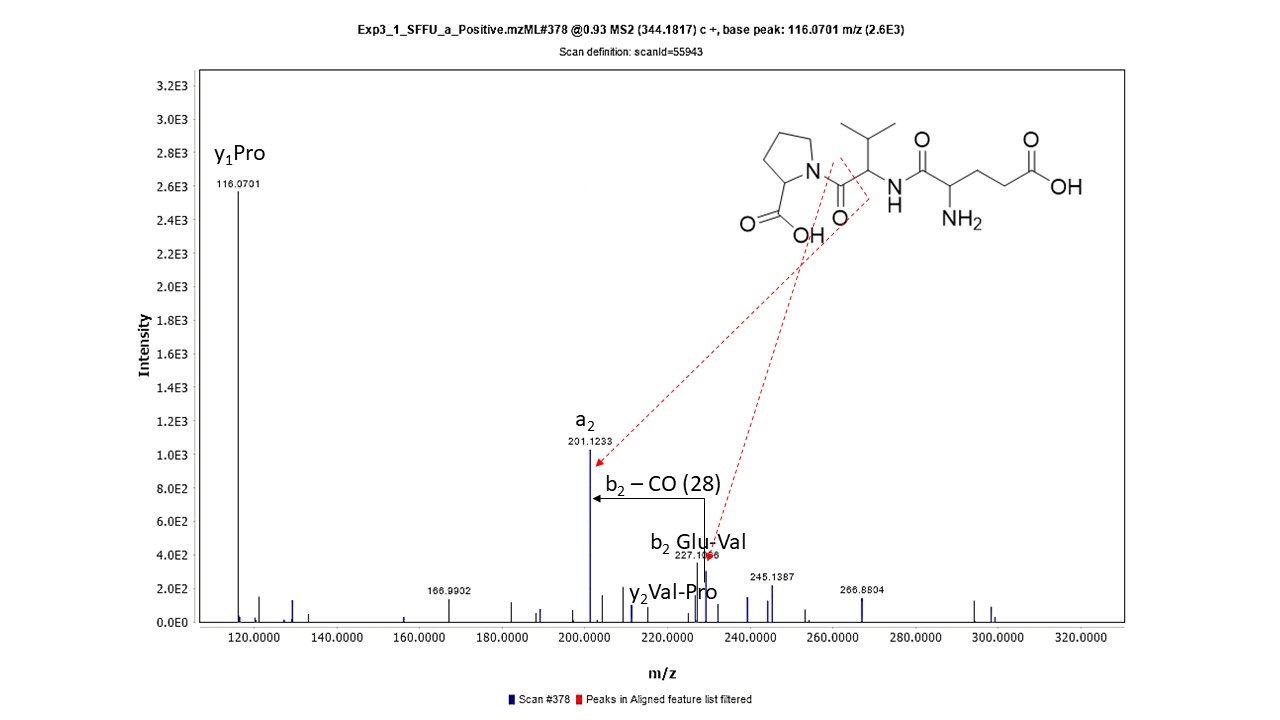


**Suppl. Fig. S16:** MS^2^ spectrum of 5, [M+H]^+^ at *m/z* 344.1820, Glutamyl-valyl-proline


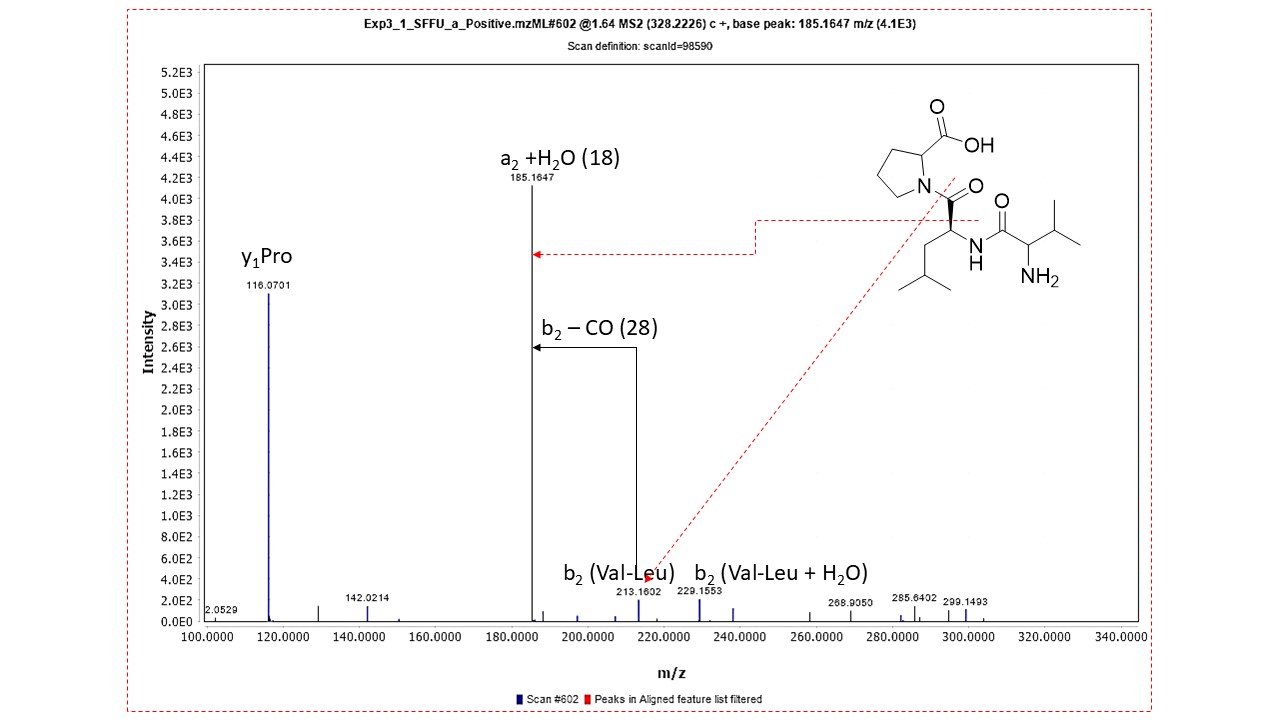


**Suppl. Fig. S17:** MS^2^ spectrum of 16, [M+H]^+^ at *m/z* 328.224, Valyl-leucyl-proline


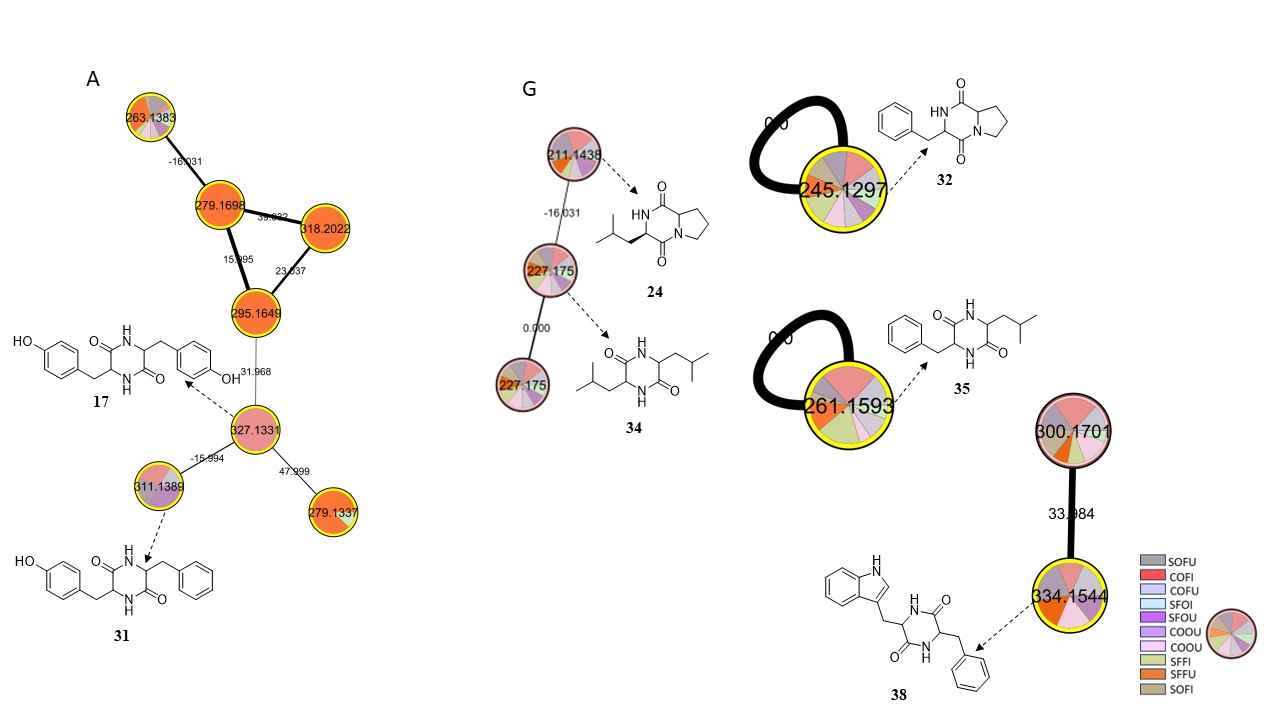


**Suppl. Fig. S18.** Annotated cyclopeptides and their distribution in the FBMN


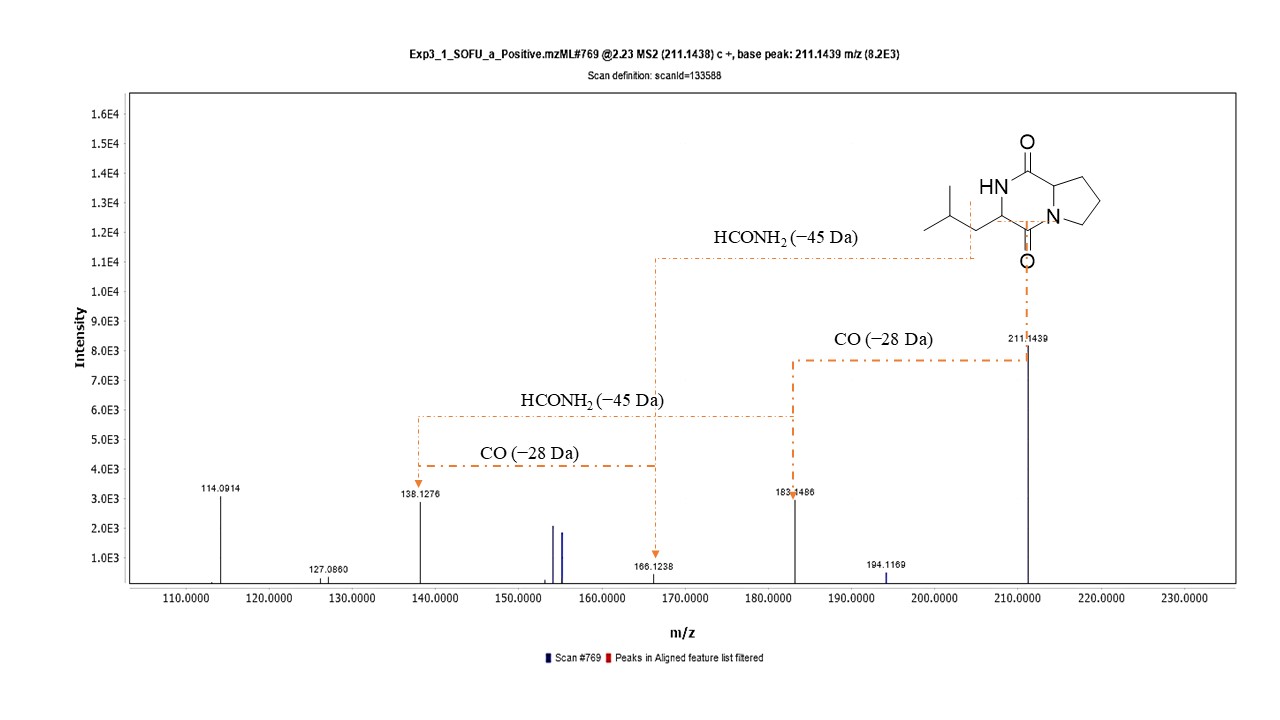


**Suppl. Fig. S19:** MS^2^ spectrum of 24, [M+H]^+^ at *m/z* 211.1438, Cyclo-(pro-leu)


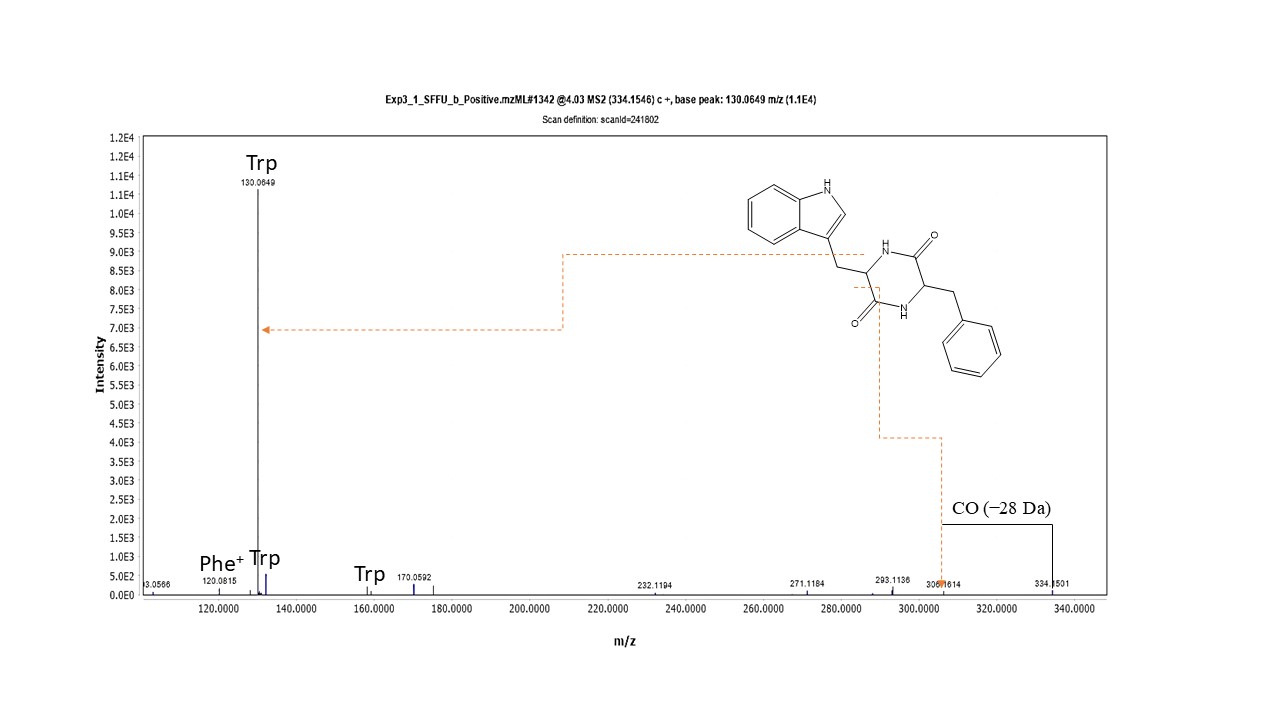


**Suppl. Fig. S20:** MS^2^ spectrum of 38, [M+H]^+^ at *m/z* 334.1544, Cyclo-(phe-trp)


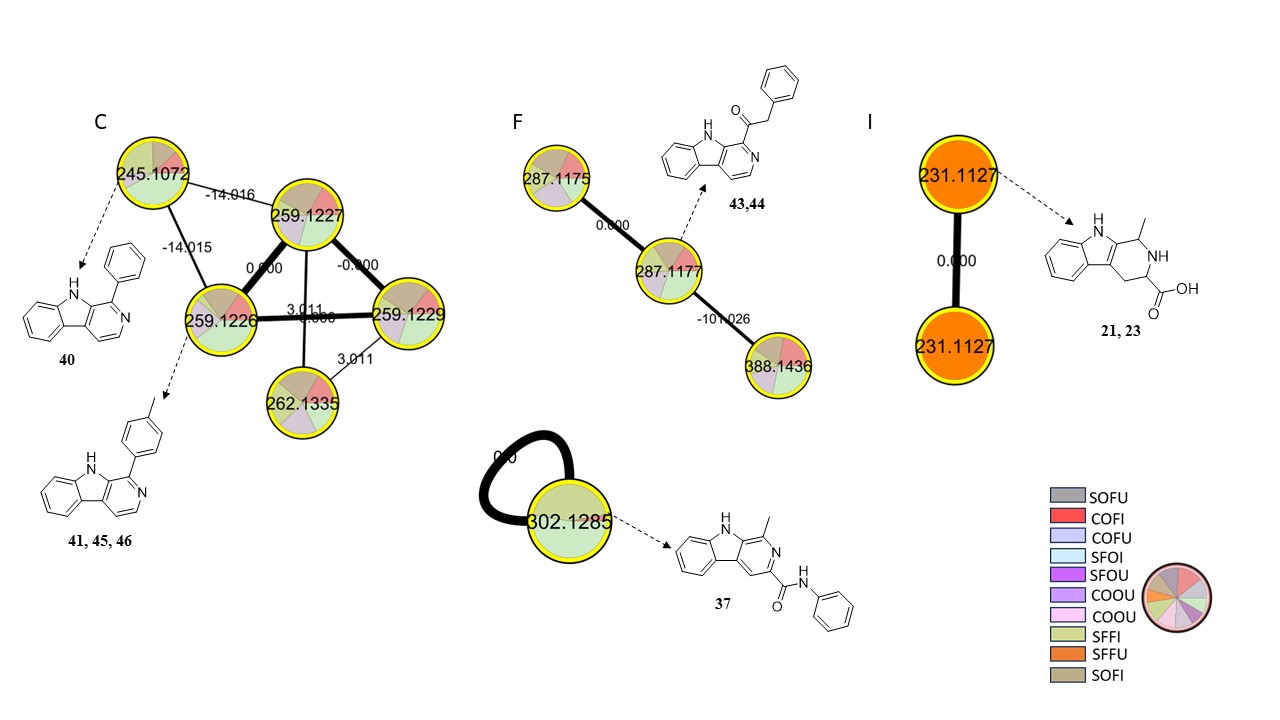


**Suppl. Fig. S21:** Annotated Indole alkaloids and their distribution in the FBMN


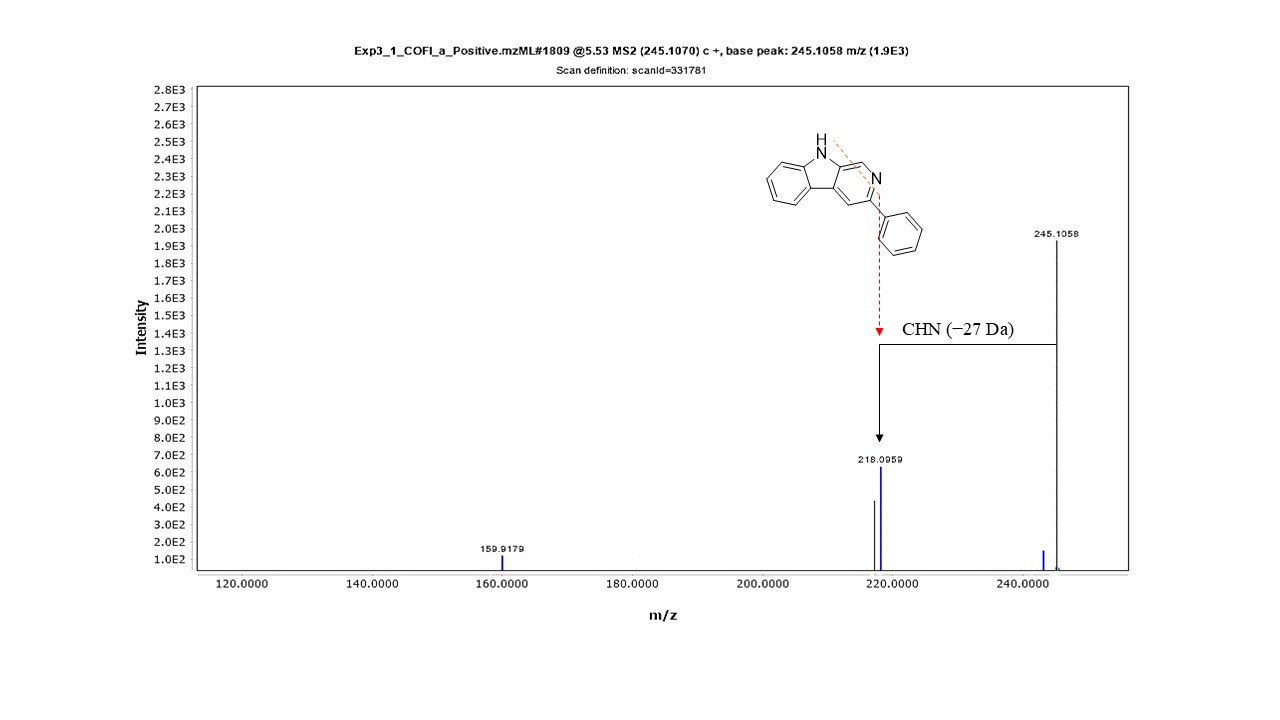


**Suppl. Fig. S22:** MS^2^ spectrum of 40, [M+H]^+^ at *m/z* 245.1072, 3-phenyl-9H-pyrido[3,4b] indole


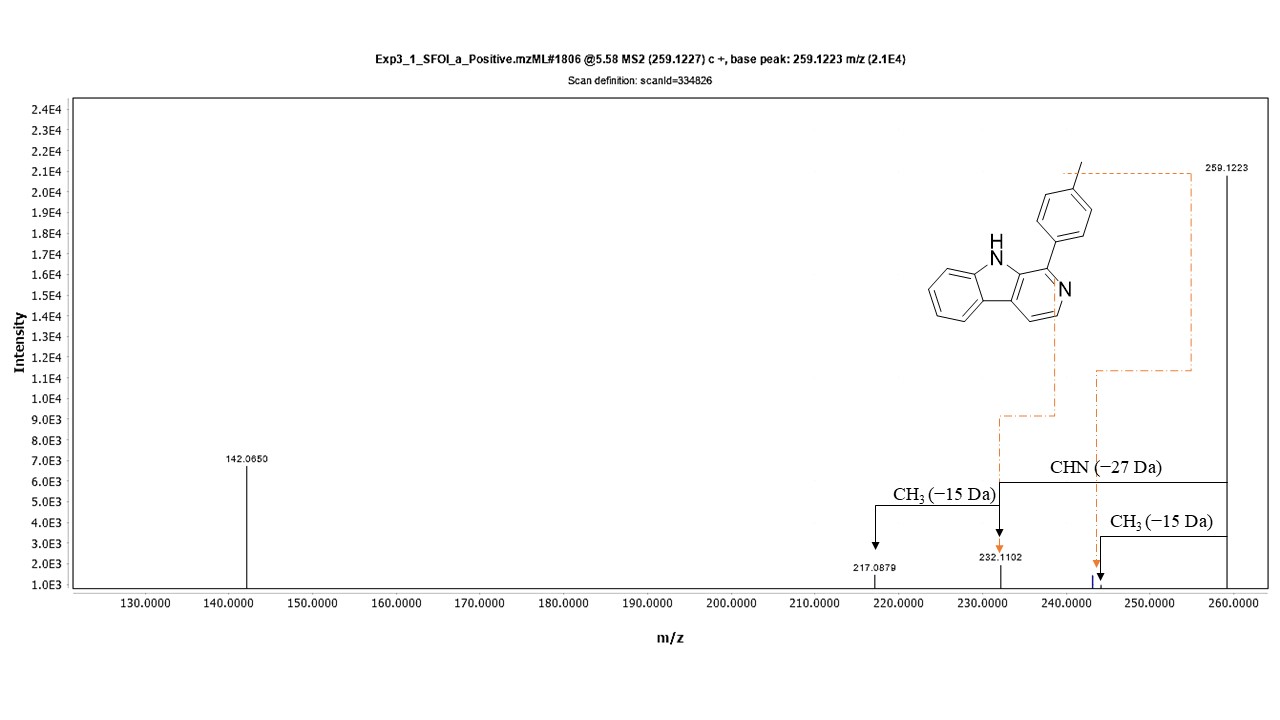


**Suppl. Fig. S23:** MS^2^ spectrum of 41, 45, 46 [M+H]^+^ at *m/z* 259.1127, 1-(4-methyl phenyl)-9*H*-pyrido[3,4-b]indole


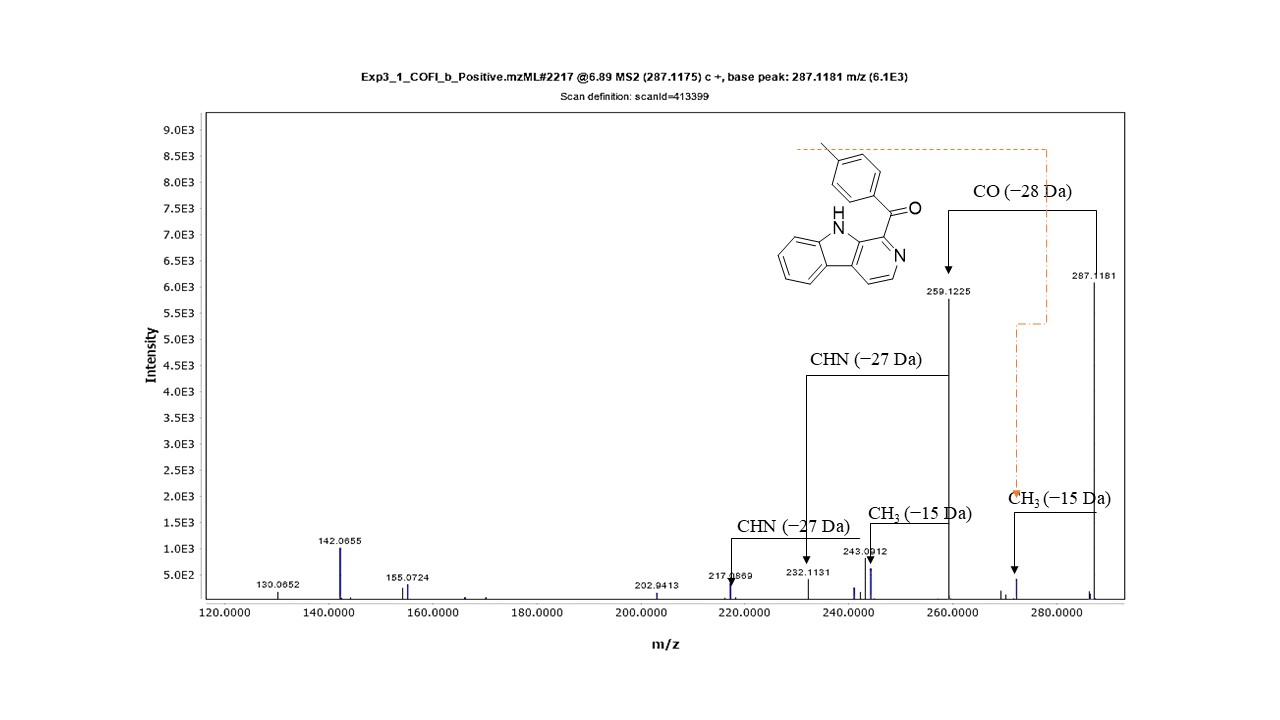


**Suppl. Fig. S24:** MS^2^ spectrum of 43 [M+H]^+^ at *m/z* 287.1181, 2-phenyl-1-(9H-pyrido[3,4-b] indol-1-yl) ethenone


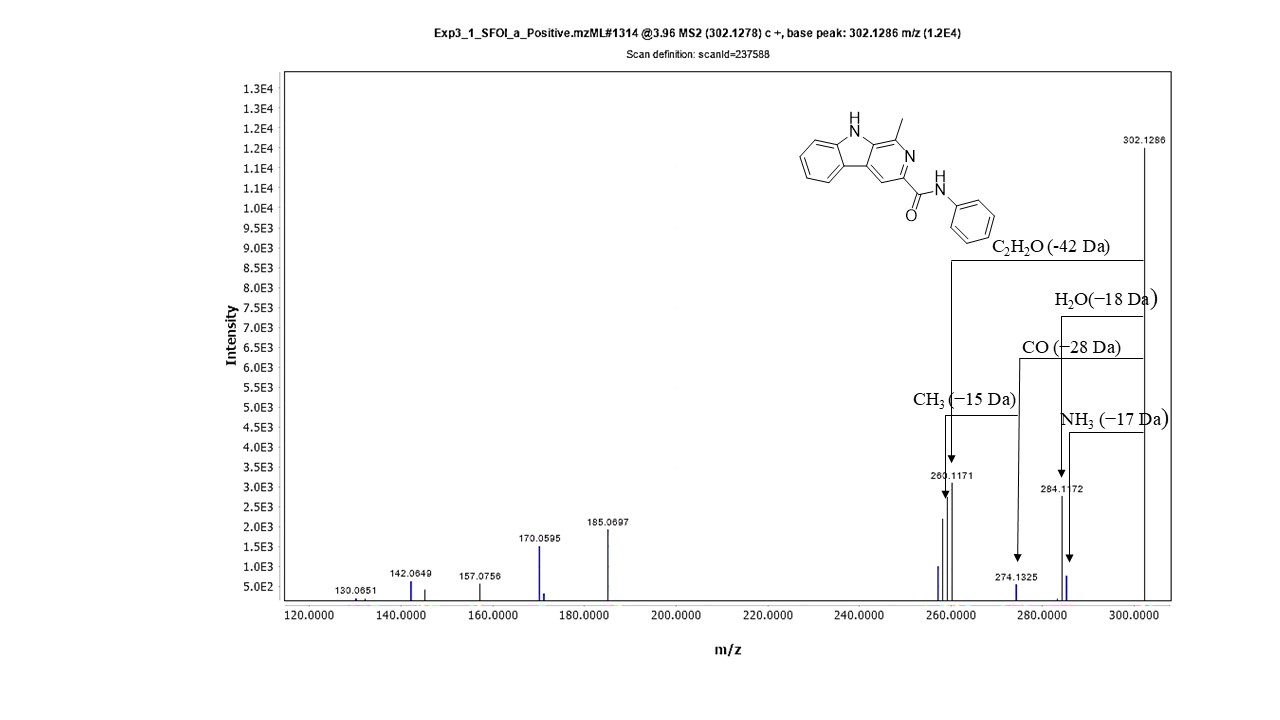


**Suppl. Fig. S25:** MS^2^ spectrum of 37 [M+H]^+^ at *m/z* 302.1286, 1-methyl-N-phenyl-9H-pyrido[3,4-b] indole-3-carboxamide


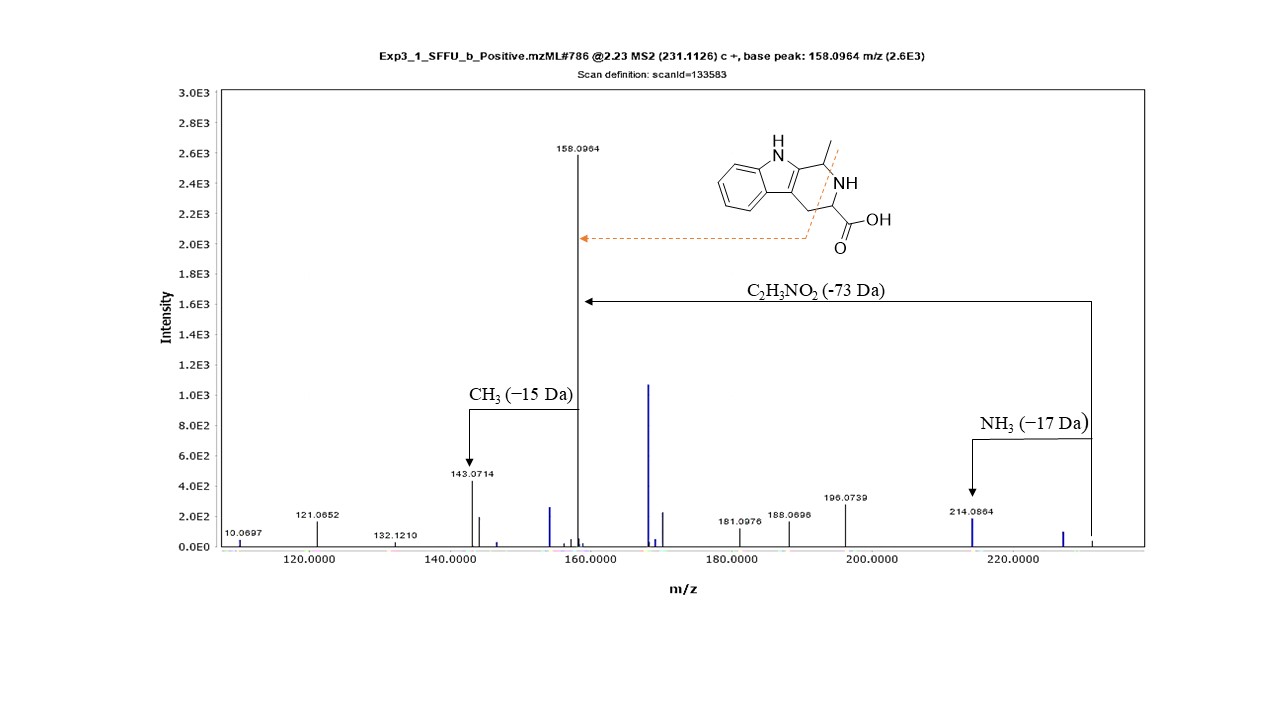


**Suppl. Fig. S26:** MS^2^ spectrum of 21, 23 [M+H]^+^ at *m/z* 231.1127, Tetrahydroharman-3-carboxylic acid


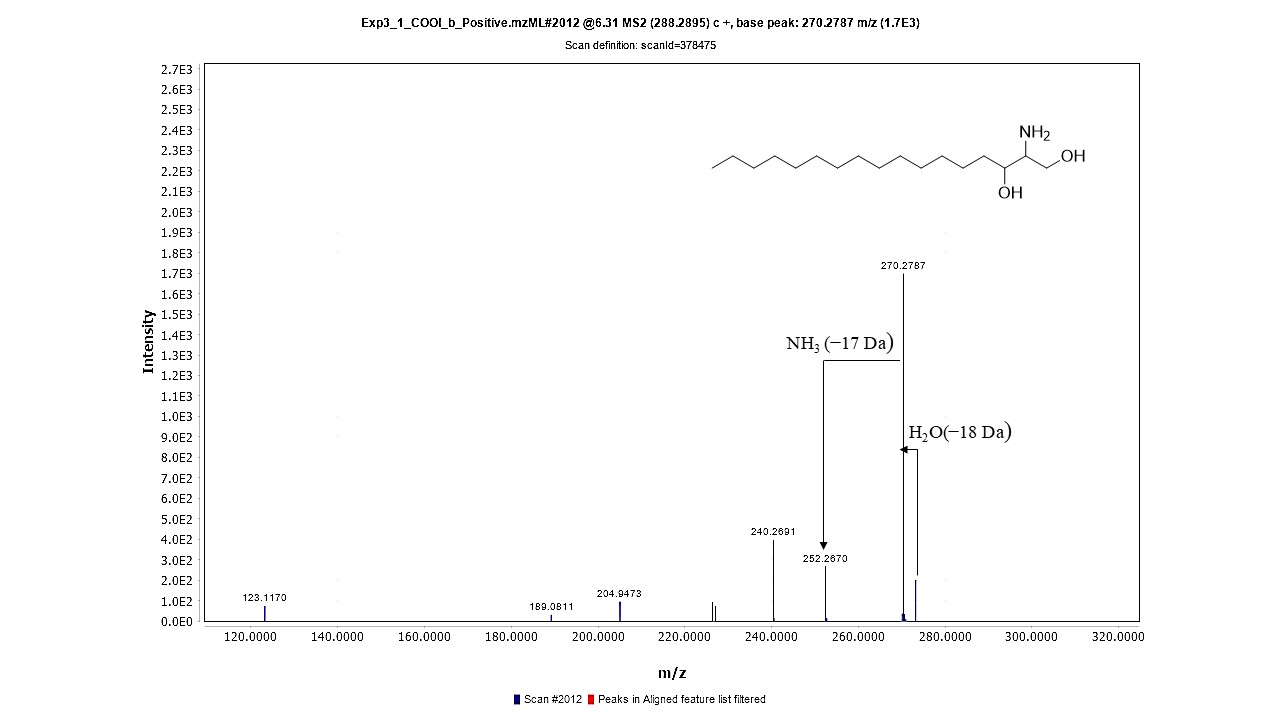


**Suppl. Fig. S27:** MS^2^ spectrum of 50, 52 [M+H]^+^ at *m/z* 408.3085, Diacetylsphingosine


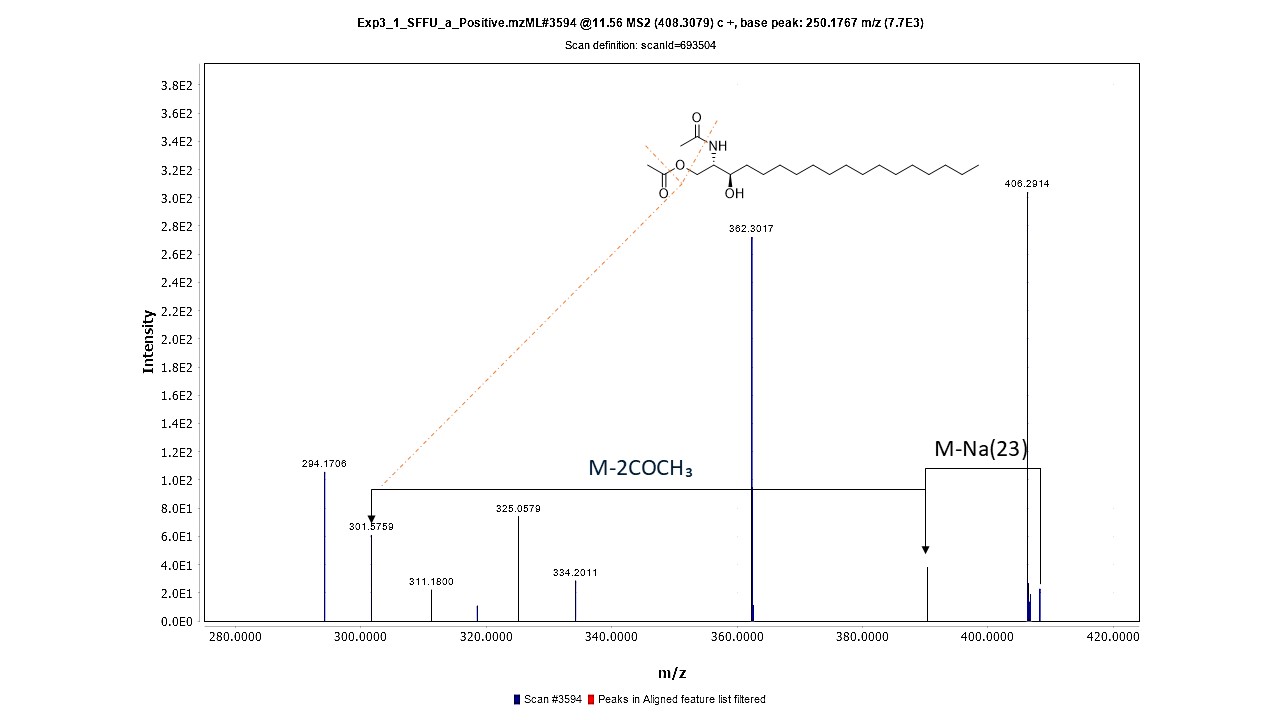


**Suppl. Fig. S28:** MS^2^ spectrum of 50, 52 [M+H]^+^ at *m/z* 408.3085, Diacetylsphingosine


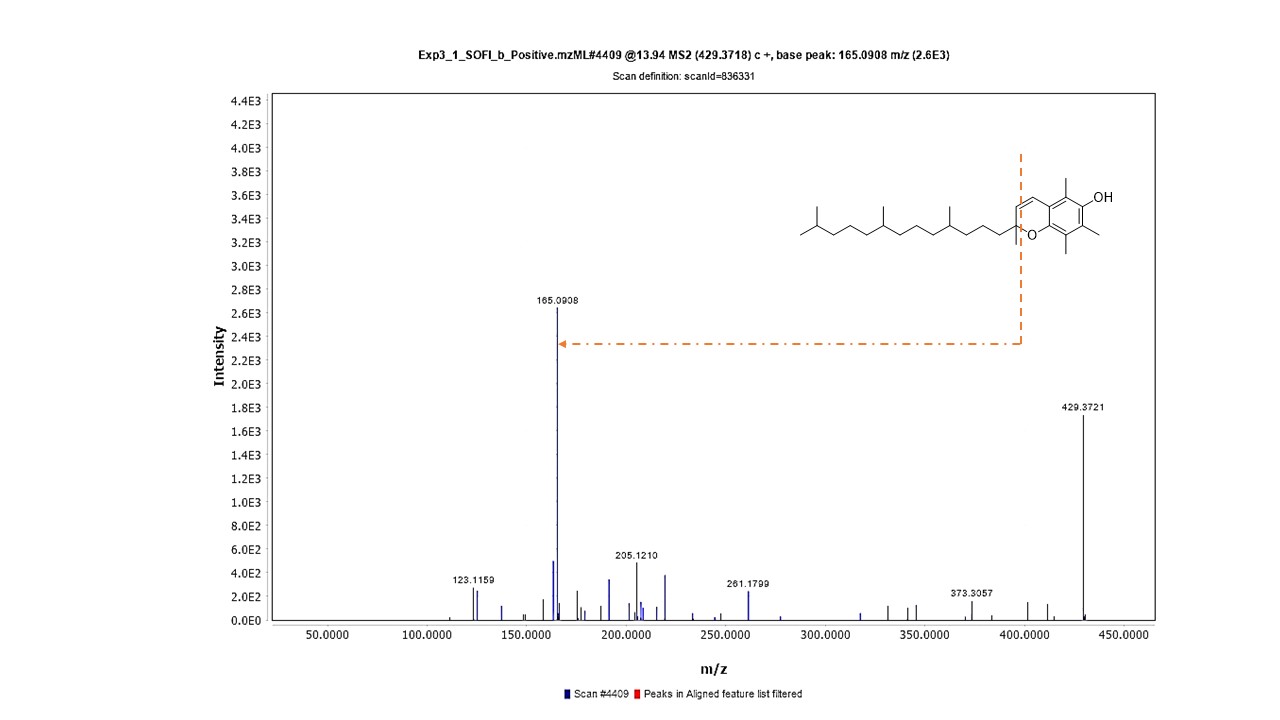


**Suppl. Fig. S29: MS2 spectrum of 55 [M+H]^+^ at *m/z* 429.3721, Dehydro-α-tocopherol**


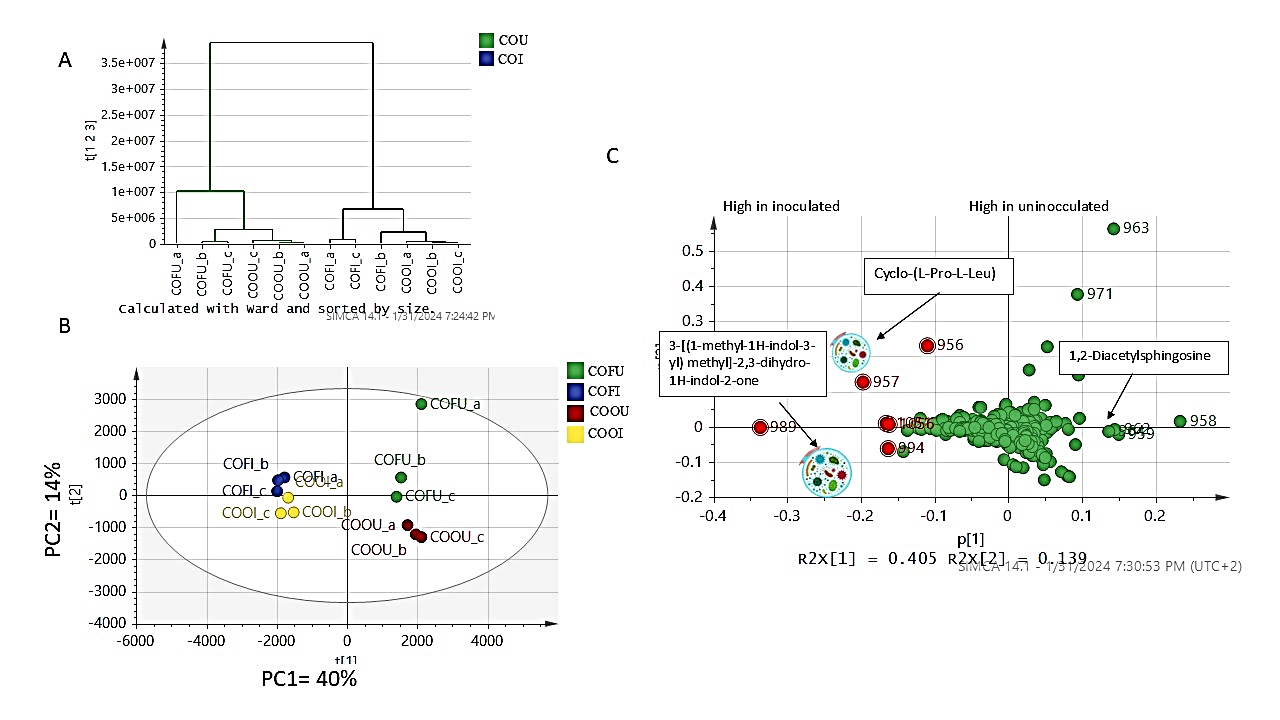


**Suppl. Fig. S30:** UPLC–HRMS/MS based PCA of all corn oils samples. (A) HCA plot. (B) Score plot of PC1 *vs* PC2 scores. (C) Loading plot for PC1 & PC2 contributing metabolites and their assignments.


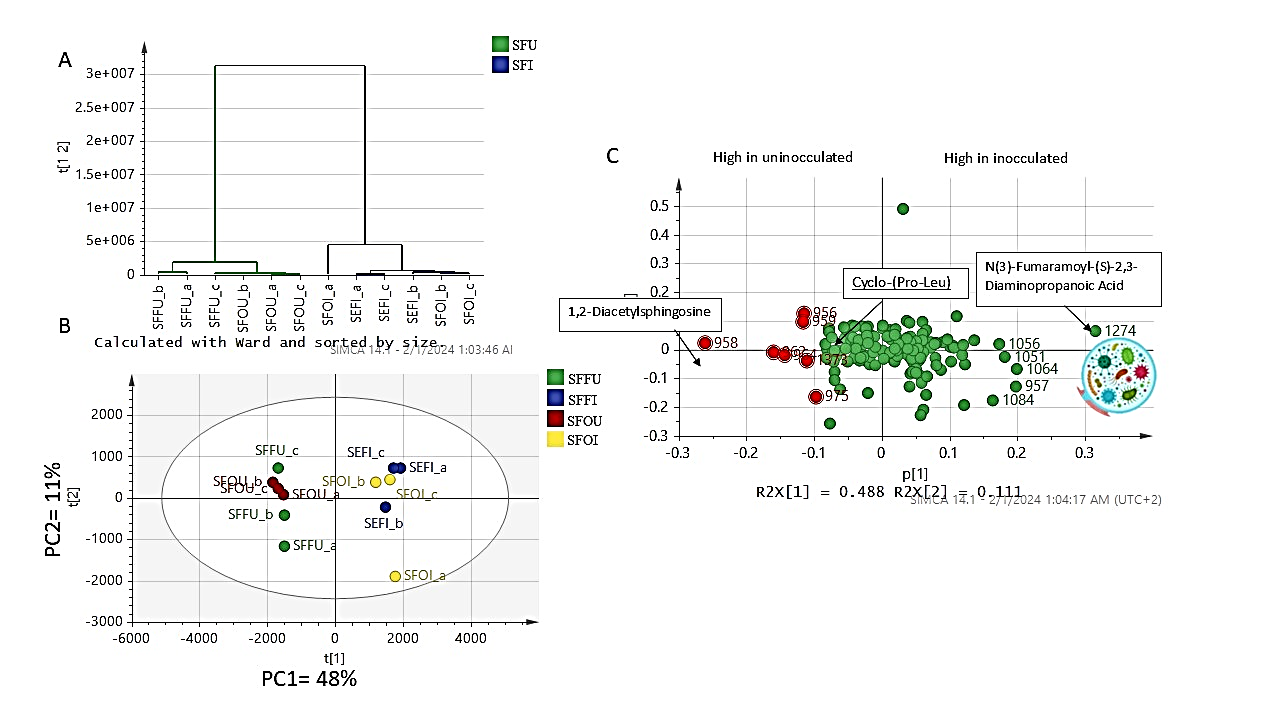


**Suppl. Fig. S31:** UPLC–HRMS/MS based PCA of all sunflower oil samples. (A) HCA plot. (B) Score plot of PC1 *vs.* PC2 scores. (C) Loading plot for PC1 & PC2 contributing metabolites and their assignments.

**Suppl. Fig. S32:** Proposed biotransformation reactions of diacetyl sphingosine under the action of gut microbiota as detected using UPLC-MS/MS in the positive mode

# **References**

1 Abib, B., Afifi, S. M., El-Din, M. G. S. & Farag, M. A. How do cultivar origin and stepwise industrial processing impact Sesamum indicum seeds’ metabolome and its paste and in relation to their antioxidant effects? A case study from the sesame industry. Food Chemistry 420, 136134 (2023).

2 Cao, Y. et al. Xinmaikang-mediated mitophagy attenuates atherosclerosis via the PINK1/Parkin signaling pathway. Phytomedicine 119, 154955 (2023).

3 Chen, L. et al. Presence of exopeptidase-resistant and susceptible peptides in a bacterial protease digest of corn gluten. Journal of Agricultural and Food Chemistry 67, 11948-11954 (2019).

4 He, H. et al. Revealing Medicinal Constituents of Bistorta vivipara Based on Non-Targeted Metabolomics and 16S rDNA Gene Sequencing Technology. Molecules 29, 860 (2024).

5 He JiaLiang, H. J., Zeng MaoMao, Z. M., Zheng ZongPin, Z. Z., He ZhiYong, H. Z. & Chen Jie, C. J. Simultaneous determination of N ε-(carboxymethyl) lysine and N ε-(carboxyethyl) lysine in cereal foods by LC-MS/MS. (2014).

6 Taowen, P. et al. Study on the action mechanism of the peptide compounds of Wuguchong on diabetic ulcers, based on UHPLC-Q-TOF-MS, network pharmacology and experimental validation. Journal of Ethnopharmacology 288, 114974 (2022).

7 Song, X. et al. Poria cocos Attenuated DSS-Induced Ulcerative Colitis via NF-κB Signaling Pathway and Regulating Gut Microbiota. Molecules 29, 2154 (2024).

8 Strehmel, N., Böttcher, C., Schmidt, S. & Scheel, D. Profiling of secondary metabolites in root exudates of Arabidopsis thaliana. Phytochemistry 108, 35-46 (2014).

9 Li, X., Fan, P., Zang, M. & Xing, J. Rapid determination of oligopeptides and amino acids in soybean protein hydrolysates using high‐resolution mass spectrometry. Phytochemical Analysis 26, 15-22 (2015).

10 Mróz, M., Parchem, K., Jóźwik, J., Domingues, M. R. & Kusznierewicz, B. The Impact of Different Drying Methods on the Metabolomic and Lipidomic Profiles of Arthrospira platensis. Molecules 29, 1747 (2024).

11 Wang, X. et al. Rapid characterizaiton of chemical constituents of the tubers of Gymnadenia conopsea by UPLC–Orbitrap–MS/MS Analysis. Molecules 25, 898 (2020).

12 Gutsche, B. & Herderich, M. High-performance liquid chromatography-electrospray ionisation-tandem mass spectrometry for the analysis of 1, 2, 3, 4-tetrahydro-β-carboline derivatives. Journal of Chromatography A 767, 101-106 (1997).

13 Ashigai, H. et al. Roasted barley extract affects blood flow in the rat tail and increases cutaneous blood flow and skin temperature in humans. Journal of agricultural and food chemistry 66, 1251-1257 (2018).

14 Thapa, B. B. et al. Metabolic Comparison and Molecular Networking of Antimicrobials in Streptomyces Species. International Journal of Molecular Sciences 25, 4193 (2024).

15 Ye, X. et al. Chemical characterization and DPP-IV inhibitory activity evaluation of tripeptides from Gynura divaricata (L.) DC. Journal of Ethnopharmacology 292, 115203 (2022).

16 Wei, J. et al. Curcumae Rhizoma-combined with Sparganii Rhizoma in the treatment of liver cancer: Chemical analysis using UPLC-LTQ-Orbitrap MSn, network analysis, and experimental assessment. Frontiers in Pharmacology 13, 1027687 (2022).

17 Canu, N. et al. Incorporation of Non‐canonical Amino Acids into 2, 5‐Diketopiperazines by Cyclodipeptide Synthases. Angewandte Chemie 130, 3172-3176 (2018).

18 Li, Y. et al. Integrated Microbiota and Metabolome Analysis to Assess the Effects of the Solid-State Fermentation of Corn–Soybean Meal Feed Using Compound Strains. Microorganisms 11, 1319 (2023).

19 Cai, Z., He, J., Jiang, J., Zhao, Z. & Shu, Y. Systematic investigation of the material basis, multiple mechanisms and quality control of Simiao Yong'an decoction combined with antibiotic in the treatment of sepsis. Phytomedicine 116, 154910 (2023).

20 Perruchon, O. et al. Combination of UHPLC-MS/MS-molecular networking approach and FTICR-MS for the metabolic profiling of Saccharomyces cerevisiae. Journal of Pharmaceutical and Biomedical Analysis 195, 113857 (2021).

21 Al-Nemi, R., Makki, A. A., Sawalha, K., Hajjar, D. & Jaremko, M. Untargeted metabolomic profiling and antioxidant capacities of different solvent crude extracts of Ephedra foeminea. Metabolites 12, 451 (2022).

22 Farag, M. A., Ragab, N. A. & Maamoun, M. A. I. Metabolites profiling of Sapota fruit pulp via a multiplex approach of gas and ultra performance liquid chromatography/mass spectroscopy in relation to its lipase inhibition effect. PeerJ 12, e17914 (2024).

23 Farag, M. A., Shakour, Z. T. A., Elmassry, M. M. & Donia, M. S. Metabolites profiling reveals gut microbiome-mediated biotransformation of green tea polyphenols in the presence of N-nitrosamine as pro-oxidant. Food Chemistry 371, 131147 (2022).

24 Choi, J.-H. et al. A novel sphingosine with osteoclast-forming suppressing activity, from the edible mushroom Grifola gargal. Tetrahedron 69, 8609-8611 (2013).

25 Liu, Z. et al. Exploring the possible mechanism (s) underlying the nephroprotective effect of Zhenwu Decoction in diabetic kidney disease: An integrated analysis. Phytomedicine 119, 154988 (2023).
